# Three-Dimensional Gene Map of Cancer Cell Types: Structural Entropy Minimisation Principle for Defining Tumour Subtypes

**DOI:** 10.1038/srep20412

**Published:** 2016-02-04

**Authors:** Angsheng Li, Xianchen Yin, Yicheng Pan

**Affiliations:** 1 State Key Laboratory of Computer Science, Institute of Software, Chinese Academy of Sciences, 4# South Fourth Street, Zhong Guan Cun, Beijing, 100190, P. R. China; 2 University of Chinese Academy of Sciences, Beijing, P.R. China

## Abstract

In this study, we propose a method for constructing cell sample networks from gene expression profiles, and a structural entropy minimisation principle for detecting natural structure of networks and for identifying cancer cell subtypes. Our method establishes a three-dimensional gene map of cancer cell types and subtypes. The identified subtypes are defined by a unique gene expression pattern, and a three-dimensional gene map is established by defining the unique gene expression pattern for each identified subtype for cancers, including acute leukaemia, lymphoma, multi-tissue, lung cancer and healthy tissue. Our three-dimensional gene map demonstrates that a true tumour type may be divided into subtypes, each defined by a unique gene expression pattern. Clinical data analyses demonstrate that most cell samples of an identified subtype share similar survival times, survival indicators and International Prognostic Index (IPI) scores and indicate that distinct subtypes identified by our algorithms exhibit different overall survival times, survival ratios and IPI scores. Our three-dimensional gene map establishes a high-definition, one-to-one map between the biologically and medically meaningful tumour subtypes and the gene expression patterns, and identifies remarkable cells that form singleton submodules.

One of the challenges of cancer treatment is targeting specific therapies to pathogenetically distinct tumour types to maximise treatment efficacy and minimise toxicity. Traditionally, cancer classification has been based on the morphological appearance of the tumour; however, this approach has serious limitations. Tumours with similar histopathological appearances can have different clinical courses and exhibit different responses to therapy. Molecular heterogeneity within individual cancer diagnostic categories is also evident in the variable presence of chromosomal translocations, tumour suppressor genes deletions and numerical chromosomal abnormalities. Cancer classification is difficult because the classification relies on specific biological insights, instead of on systematic, comprehensive, global and unbiased methods for identifying tumour subtypes.

Over the past decade, the increased availability of large-scale gene expression profiles have led researchers to propose a number of new approaches for classifying tumour types or subtypes based on gene expression analyses. Golub *et al*.^1^ have proposed a neighbour analysis to distinct known types, and a “class predictor” that assigns a new sample to a known class purely based on the gene expression profiles, and have verified their methods using an acute leukaemia dataset. Alizadeh *et al*.^2^ have proposed a method based on hierarchical clustering, which divides the type of diffuse large B-cell lymphomas into two subtypes. Ramaswamy *et al*.^3^ have proposed a “classifier” based on a support vector machine (SVM) and have analysed the accuracy of true type predictions for both the snap-frozen human tumour and normal tissue specimens. Yeoh *et al*.^4^ have analysed sets of genes that define certain subtypes. Bhattacharjee *et al*.^5^ have classified human lung carcinomas by using hierarchical clustering and have verified the classification by a selected set of gene expression profiles. Su *et al*.^6^ have analyzed differences in gene expression between human and mouse transcriptomes by using a selected set of gene expression profiles. Pomeroy *et al*.^7^ have proposed a classification method for tumour types of the central nervous system based on a learning algorithm and have verified their result by a selected set of gene profiles. Monti *et al*.^8^ have proposed a learning algorithm to classify the tumour types and have verified their results according to the similarity with the true types. Yang and Naiman^9^ have proposed a learning algorithm to select a small set of genes that are distinct to known tumour types. Haferlach *et al*.^10^ proposed a learning algorithm to classify the leukemia subtypes based on a large number of clinical samples.

Theoretical biologists have found that gene expression patterns are important for tumour classification. Ao *et al*.^11^ have shown that a 20-node network may generate 32 attractors, implying that gene expression patterns provide a better classification scheme than the dominating scheme based on DNA and mutations. Wang *et al*.^12^ have reported the identification and prediction of liver tumour subtypes from an endogenous network. Zhu *et al*.^13^ have discovered a hierarchical property of prostate cell types.

Community detection is to identify the natural communities of a naturally evolving network. It provides a systematic and global approach to understanding the natural structures of the real world networking systems and is one of the major topics in network theory^14^,^15^. According to Darwin’s theory^16^, animals from ants to people form social groups in which most individuals work for the common good. Similarly, we may have that individuals of a naturally evolving network may form natural communities. Li *et al*.^17^,^18^ have shown that the natural communities of a network maybe detected by an algorithm which follows the principle of the formation of the natural communities, and that (two-dimensional) structural entropy minimisation is the principle for detecting the natural communities in networks. This progress implies that tumour types and subtypes may be identified by the community detection algorithms on the basis of structural entropy minimisation, due to the fact that natural community detection is a classification by following the principle of the organisation of the network. In the present paper, we show that structural entropy minimisation is the principle for detecting natural structures of networks. Specifically, we show that structural entropy minimisation is the principle for identifying cancer cell types and subtypes.

We establish a novel method to define tumour types and subtypes. To establish our method, we have to resolve two challenges. The first is to detect the two- and three-dimensional natural structures of a naturally evolving network, and the second is to construct a network from the unstructured gene expression data of cancer cell samples such that the constructed network captures the nature and laws of the gene expression data of the cancers. We resolve the two challenges by using our new notion of high-dimensional structural entropy of graphs.

Given a network *G* and a natural number *K*, we define the *K*-dimensional structural entropy of *G*, denoted 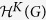, to be the least overall number of bits required to determine the *K*-dimensional code of the node that is accessible from random walk with stationary distribution in *G*. The *K*-dimensional structural entropy of a graph quantitatively measures the non-determinism or uncertainty of the natural *K*-dimensional structure of the graph. The *K*-dimensional structural entropy of network *G* explores that the community structure of *G* that realises the two-dimensional structural entropy of *G* is the natural community structure of *G*, and that the three-dimensional structure of *G* that realises the three-dimensional structural entropy of *G* is the natural three-dimensional structure of *G*. Therefore, *K*-dimensional structural entropy minimisation is the principle of the natural *K*-dimensional structure of networks for *K* > 1. This result demonstrates that although there are many reasons and causes for the formation of natural communities such as social groups in a society, interests of organisations and games in a competing system etc, the minimisation of non-determinism or equivalently, the minimisation of uncertainty is the unified measure for the divergent causes of the formation of natural structures of a networking system. Furthermore, the structural entropy of graphs implies that one-dimensional structural entropy minimisation is the principle of a natural network of large-scale unstructured data.

Our method consists of an algorithm, denoted 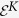, to detect the *K*-dimensional structure of a network to minimise the *K*-dimensional structural entropy of the network for each *K* > 1, and algorithms 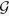 and 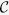 to construct a cell sample network from the gene expression profiles of a cancer by minimising the one-dimensional structural entropy of graphs. We implement the experiments of our algorithms to classify tumour types and subtypes for cancers and healthy tissues. Experiments show that our method defines meaningful types and subtypes for cancer cell samples.

We also compare the classifications given by our algorithms with the two frequently used community detecting algorithms: the first is the modularity maximisation algorithm proposed by Clauset, Newman and Moore^19^, denoted 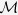, and the second is the minimisation of expression length algorithm proposed by Rosvall and Bergstrom^20^, denoted InforMap or 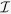.

To evaluate the classifications of the types and subtypes found by our algorithms 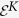 and the existing algorithms 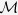 and 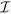, we analyse the similarity between the true tumour types and the modules identified by the algorithms, establish the gene map of the modules, and analyse the performance of the found types and subtypes in clinical data. The experimental results demonstrate that our algorithms establish a high-definition and one-to-one three-dimensional gene map of the submodules of the cancers identified by our new algorithms. For the diffuse large B-cell lymphoma (DLBCL), our results demonstrate that most of the cell samples within a module or submodule identified by our algorithms share similar survival times, survival indicators and International Prognostic Index (IPI) scores, and indicate that the distinct modules or submodules identified using our structural entropy minimisation algorithms noticeably differ in overall survival times, survival ratios and IPI scores, and that distinct modules identified by the modularity maximisation and description length minimisation algorithms exhibit undistinguishable survival times, survival ratios and IPI scores.

## High-Dimensional Graph Structure Entropy

In a real world network, at every time step, various interactions, communications and operations may occur in the network, and the actions in the network are often unpredictable. Thus, the non-determinism of the various actions in a network is the essence of the complexity of the network. It is certainly a dynamical complexity of the network.

Our notion of structure entropy of a graph is to measure the dynamical complexity of the graph. Intuitively, for a graph *G* and a natural number *K*, the *K*-dimensional structural entropy of *G* is the least overall number of bits required to determine the *K*-dimensional code of the node that is accessible from random walk with stationary distribution in *G*. We will gradually establish the notion.

### One-dimensional structural entropy

First, we recall Shannon’s entropy function. For a probability vector *p* = (*p*_1_,…, *p*_*q*_), with 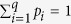, the Shannon entropy function of *p* is defined as follows:


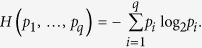


Considering the definition of *H*(*p*_1_, *p*_2_, ···, *p*_*n*_), for every *i*, *l* =  − log *p*_*i*_ is the length of the binary representation of the number 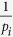, which indicates that 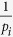 is one of the 2^*l*^ numbers. Therefore, we interpret −log *p*_*i*_ as the “self-information of *p*_*i*_”, which also indicates that −log *p*_*i*_ is the amount of information needed to determine the code of *i*. Therefore, 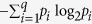 is the average amount of information required to determine the code of *i* that is picked according to the probability distribution *p* = (*p*_1_, *p*_2_, ···, *p*_*n*_).

For a connected graph *G* = (*V*, *E*) with *n* nodes and *m* edges, we define one-dimensional structural entropy or the positioning entropy of *G* by using the entropy function *H*. For each node *i* ∈ {1,2, ···, *n*}, let *d*_*i*_ be the degree of *i* in *G*, and let 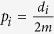. Then, the stationary distribution of random walk in *G* is described by the probability vector *p* = (*p*_1_, *p*_2_, ···, *p*_*n*_). We define the *one-dimensional structural entropy* or *positioning entropy of G* as follows:


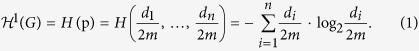


By definition, 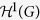 is the average number of bits required to determine the one-dimensional code of the node that is accessible from the random walk with stationary distribution in *G*.

(Remark: (i) If the degree *d*_*i*_ of node *i* is 0 for some *i*, then the definition of 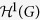 is invalid. (ii) The definition of 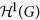 may be extended to disconnected graphs; in such cases, 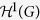 is the weighted average of 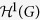 for all of the connected-component *G*_*i*_ of *G*. Of course, we assume that for the graph of a singleton with no edge, the one-dimensional structural entropy of the graph is 0 because, a random walk cannot occur in such a graph. (iii) The definition of 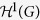 differs from the Shannon entropy for the randomly selection of a node in *G* as follows: 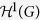 is a dynamical notion measuring the complexity of the random walk in the graph, whereas the Shannon entropy is a static notion for a probabilistic distribution).

The one-dimensional structural entropy (or positioning entropy) is interesting for the following reasons: (i) the notion is a dynamical version of the Shannon’s entropy in graphs, (ii) positioning is a basic operation for network applications, and (iii) the first step for a rigorous study on unstructured big data is perhaps to structure the data, for which one-dimensional structural entropy minimisation could be the fundamental principle. Item (iii) is extremely important, because it means that the minimisation of one-dimensional structural entropy could be the principle to identify the natural network from unstructured big data. In the present paper, we will propose such an algorithm to construct cell sample networks for cancers from the unstructured gene expression profiles.

### Two-dimensional structural entropy

For a naturally evolving network *G* = (*V*, *E*), individuals form social groups primarily through self-organising behaviours. Therefore, self-organisation behaviours lead to a natural community structure of the network. To detect the natural communities of a network, we must understand the principle of self-organisation of naturally evolving networks.

Suppose that 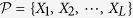 is a partition of *V*, in which each *X*_*j*_ is defined as a module or a community. Using 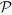, we encode a node *v* by a pair (*i*, *j*) such that *j* is the code of the community *X* containing *v* (referred to as the *global code*), and *i* is the code of node *v* within its own community *X* (referred to as the *local code*). In this case, suppose that *v* is the node that is accessible from the random walk with stationary distribution in *G*. We define the number of bits required to determine the pair (*i*, *j*) of the codes of *v*. The following two scenarios may occur:

- Case 1: *v* is accessible from node *u* in the community of *v*. In this case, only the local code *i* of *v* within its own community must be determined because the code of its community is already known before the random walk.
- Case 2: *v* is accessible from a node outside *v*’s own community. In this case, both the local and global codes of *v*, or the pair (*i*, *j*), must be determined.

If 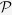 is a well-defined community structure of *G*, then the probability of Case 2 occurring is small. In this scenario, the number of bits required to determine the two-dimensional code (*i*, *j*) of the node that is accessible from the random walk is significantly smaller than the one-dimensional structural entropy or positioning entropy of *G*.

The ideas presented above motivated us to define the *two-dimensional structural entropy*, which is also referred to as the *module entropy* or *local positioning entropy* of a graph.

For a connected graph *G* = (*V*, *E*), suppose that 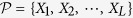 is a partition of *V*. We define the *structural entropy of G by* 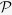 as follows:


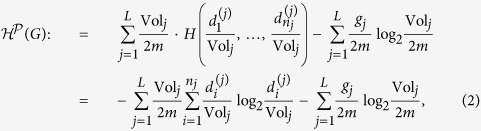


where *L* is the number of modules in partition 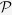, *n*_*j*_ is the number of nodes in module *X*_*j*_, 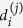 is the degree of the *i*-th node of *X*_*j*_, Vol_*j*_ is the volume of module *X*_*j*_ (the sum of the degrees of the nodes in module *X*_*j*_), and *g*_*j*_ is the number of edges with exactly one endpoint in module *X*_*j*_.

The two-dimensional structural entropy of a graph *G* is defined as follows:


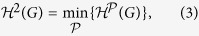


where 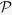 runs over all of the partitions of *G*.

For a network that naturally evolves in nature and society, we propose the following *self-organisation hypothesis*: the self-organisation of individuals in the network minimises the non-determinism of the structure of the network. Assuming the self-organisation hypothesis, the algorithm minimising the two-dimensional structural entropy of the graph produces the natural community structure of the network.

The definition of 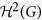 clearly reveals that minimising the non-determinism of the community structures is a principle of the natural community structures and network self-organisation in nature and society. As a matter of fact, Li, Li and Pan^17^ and Li *et al*.^18^ have shown that two-dimensional structural entropy minimisation is the principle for detecting the natural community structure of networks.

### High-dimensional structural entropy

Real world networks generally have a hierarchical structure such that a module of a network may consist of quite a few submodules, which leads to a natural extension of the two-dimensional structural entropy to high-dimensional cases.

To define high-dimensional structural entropy, we introduce a partitioning tree of graphs. First, we consider the two-dimensional case. For a graph *G* = (*V*, *E*), and a partition 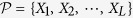 of *V*, we interpret the partition 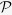 by a partitioning tree 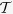 of hight 2 as follows: 1) first, we introduce the root node *λ*, and define a set of nodes *T*_*λ*_ = *V*, 2) we introduce *L* immediate successors for the root node denoted 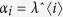, where *i* = 1, 2, ···, *L*, and associate the set *X*_*i*_ with node *α*_*i*_; thus, we define 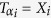, and 3) for each *α*_*i*_, we introduce |*X*_*i*_| immediate successors denoted 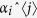 for all *j* ∈ {1, 2, ···,|*X*_*i*_|}, and each successor 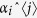 is associated with an element in *X*_*i*_; thus, we define 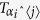 as the singleton of a node in 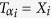.

Therefore, 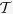 is a tree of height 2, and all of the leaves of 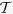 are associated with singletons. For every node 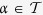, *T*_*α*_ is the union of *T*_*β*_ for all of *β* values (of the immediate successors) of *α*, and the union of *T*_*α*_ for all of the nodes *α* values at the same level of the tree 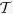 is a partition of *V*.

Thus, the partitioning tree of a graph *G* = (*V*, *E*) is a set of nodes such that each node is associated with a nonempty subset of vertices of graph *G*, and can be defined as follows:

Let *G* = (*V*, *E*) be a network. We define the *partitioning tree* 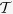 *of G* as a tree 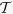 with the following properties:

1. For the root node denoted *λ*, we define the set *T*_*λ*_ = *V*.
2. For every node 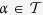, the immediate successors of *α* are 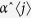 for *j* from 1 to a natural number *N* ordered from left to right as *j* increases. Therefore, 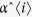 is to the left of 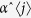 written as 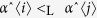, if and only if *i* < *j*.
3. For every 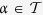, there is a subset 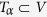 that is associated with *α*. For *α* and *β*, we use 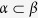 to denote that *α* is an initial segment of *β*. For every node 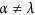, we use *α*^−^ to denote the longest initial segment of *α*, or the longest *β* such that 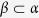.
4. For every *i*, {*T*_*α*_ | *h*(*α*) = *i*} is a partition of *V*, where *h*(*α*) is the height of *α* (note that the height of the root node *λ* is 0, and for every node 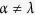, *h*(*α*) = *h*(*α*^−^) + 1).
5. For every *α*, *T*_*α*_ is the union of *T*_*β*_ for all *β*’s such that *β*^−^  = *α*; thus, 
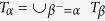
.
6. For every leaf node *α* of 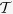, *T*_*α*_ is a singleton; thus, *T*_*α*_ contains a single node of *V*.

We define the entropy of *G* by a partitioning tree 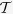 of *G*.

For a network *G* = (*V*, *E*), suppose that 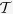 is a partitioning tree of *G*. We define the structural entropy of *G* by 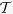 as follows:

- (1) For every 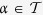, if 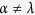, then define
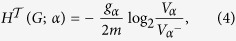
where *g*_*α*_ is the number of edges from nodes in *T*_*α*_ to nodes outside *T*_*α*_, *V*_*β*_ is the volume of set *T*_*β*_, namely, the sum of the degrees of all the nodes in *T*_*β*_. (Remark: For an edge-weighted graph *G* = (*V*, *E*), *g*_*α*_ is the sum of the weights of all the edges between *T*_*α*_ and nodes outside *T*_*α*_, and the degree of a node *v* ∈ *V* in *G* is the sum of the edge weights of all the edges incident to *v*. For a non-weighted graph, we regard the weight of an edge as 1.)
- (2) We define the structural entropy of *G* by the partitioning tree 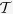 as follows:
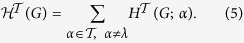
Let *G* = (*V*, *E*) be a network. We define the *K*-dimensional structural entropy of *G* as follows:
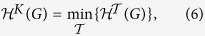

where 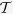 ranges over all of the partitioning trees of *G* of height *K*.

Our definition of the structural entropy explores the following *self-organisation principle*: minimising the non-determinism of a structure is the principle for the self-organisation of structures within naturally evolving networks.

In particular, the *K*-dimensional structural entropy of a graph *G* implies that the *K*-dimensional structure of *G* that minimises the *K*-dimensional structural entropy of *G* is the natural *K*-dimensional structure of *G*. Precisely, according to the definition of the *K*-dimensional structural entropy, we may define the natural structure of a graph as follows: Given a connected graph *G*, a natural number *K* > 1, a constant *δ* ≤ 1 and a *K*-level partitioning tree 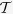 of *G*, we say that 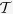 is a *δ*-natural *K*-dimensional structure of *G*, if 
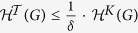
. This definition allows us to mathematically analyse the natural structures of networks. We leave it as an open question.

## Algorithm for Minimising the *K*-Dimensional Structural Entropy

In this section, we describe an algorithm for finding a partitioning tree 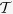 of a graph *G* of height *K* to minimise the *K*-dimensional structural entropy of *G*.

Two operators, the merging operator and the combining operator, are introduced, and a partitioning tree is developed by using the two operators.

First, we define the *merging operator*. Let 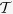 be a partitioning tree and let *α* and *β* be nodes of 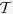 with *α* < _L_*β* (meaning that *α* is to the left of *β*) and *α*^−^  = *β*^−^  = *γ* for some *γ*. In addition, let 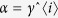, and 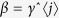 for *i* < *j*.

We define a partitioning tree below, which is obtained from 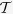 via the following merging operator: 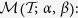


Let *T*_*α*_ = {*x*_1_, *x*_2_, ···, *x*_*M*_} and *T*_*β*_ = {*y*_1_, *y*_2_, ···, *y*_*N*_}, which are ordered as listed in the sets. Then,

1. Define *T*_*α*_ = {*x*_1_, *x*_2_, ···, *x*_*M*_, *y*_1_, *y*_2_, ···, *y*_*N*_}, which are ordered as they are listed.
2. Set *h*(*α*) ← *h*(*α*).
3. For each *s* ∈ {1, 2, ···, *M*}, define 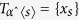 with 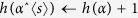.
4. For every *t* with *M* + 1 ≤ *t* ≤ *M* + *N*, define 
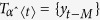
 with 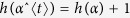.
5. Delete *β*.
6. For every *j*′ > *j*, if 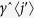 is defined, then set

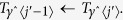


Here, we use 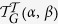 to denote the partitioning tree defined by 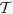 via the merging operator 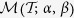 above.

We define the difference between the structural entropies of *G* obtained from a partitioning tree 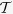 and a partitioning tree obtained from 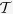 through a merging operator.

For a graph *G* = (*V*, *E*) and a partitioning tree 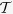 of *G*, let 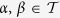 such that *α*^−^  = *β*^−^ and *h*(*α*) < *K*. Then, define 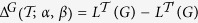, where 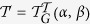. By definition, we have


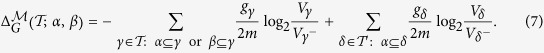


In this case, if *α* and *β* occur such that *α* < _L_*β*, *α*^−^  = *β*^−^ , *h*(*α*) < *K*, and if 
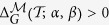
, then 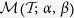 is defined and written as 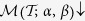.

According to equation (7), 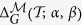 is locally computable.

Second, we define the *combining operator*. Let *G* = (*V*, *E*) be a graph, and let 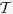 be a partitioning tree of *G*.

For any 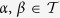, if:

1. *α*^−^  = *β*^−^  = *γ* for some *γ*, and
2. for any 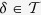, if either 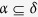 or 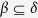, then *h*(*δ*) < *K*,

then define the combining operator 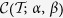 as follows:

-create a new node *ξ* with 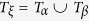 and *ξ*^−^  = *γ*,

-let the two branches with root *α* and *β* in 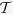 be two branches of *ξ*, while maintaining the same order as in 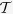.

By definition, the Δ-function with the combining operator 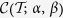 is as follows:


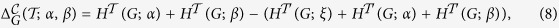


where 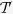 is the tree obtained from 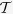 by the combing operator 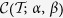.

If *α* < _L_*β* such that *α*^−^  = *β*^−^ , and if 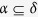 or 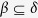 implies *h*(*δ*) < *K* for every *δ* and 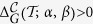. Thus, 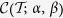 is defined and written as 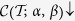.

In this case, it is clear that 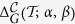 is locally computable.

Finally, we introduce our algorithm denoted 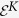 by using both the merging and combining operators. Let *G* = (*V*, *E*) be a graph. Suppose that {*v*_1_, *v*_2_, ···, *v*_*n*_} is the set of all vertices in *V* ordered as they are listed in the set. The *K*-dimensional structural entropy algorithm on *G* proceeds as follows:

1. Define the initial partitioning tree 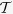 as follows:-Set *T*_*λ*_ = *V* with *h*(*λ*) = 0, and for every *i* ∈ {1, 2, ···, *n*}, define 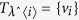 with 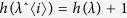.
2. If there are 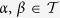 such that 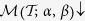, then
  1. choose *α* and *β* such that 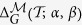 is maximised;
  2. let 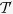 be the partitioning tree obtained from 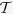 by the merging operation of 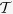 with *α* and *β*;
  3. set 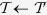; and
  4. go back to step (2).
3. If there are 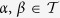 such that 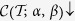, then
  - choose *α* and *β* such that 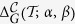 is maximised;
  - let 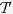 be the partitioning tree obtained from 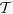 by the combining operation of 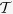 with *α* and *β*;
  - set 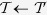; and
  - go back to step (2).
4. Otherwise, output the partitioning tree 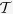, and terminate the program.

The algorithm 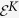 outputs a partitioning tree 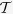 of *G*. Clearly algorithm 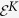 works naturally on weighted networks.

### Time complexity of algorithm 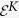


For *K* = 2, the time complexity of 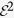 is *O*(*n*^2^) for all graphs, and is 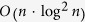 for sparse networks^17^, where *n* is the number of nodes in the graph. This algorithm is a nearly linear time algorithm for networks, which easily functions for networks that include millions of nodes. For *K* = 3, for every first level node *α* in the partitioning tree, the size of *T*_*α*_ does not decrease during the implementation of the algorithm. Therefore, |*T*_*α*_| = *M* for *M* with 1 ≤ *M* ≤ *n*. For a fixed *M* and a fixed *T*_*α*_ of size *M*, the number of operations associated with the children of *α* is the time complexity of an 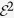 with *M* graphs of *M* nodes; thus, *O*(*M*^2^) for general graphs, and 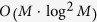 for networks. This analysis gives the time complexity with one first level node *α* of the partitioning tree *O*(*n*^3^) for all graphs and 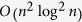 for sparse graphs. Because there are at most *n* first level nodes in the partitioning tree, the time complexity of 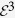 is bounded by *O*(*n*^4^) for all graphs, and 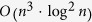 for sparse networks, which is significantly larger than that of 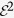.

The time complexity analysis above clearly indicates that our algorithm 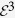 is not a hierarchical clustering algorithm with 3 levels. Because of the time complexity of 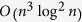 for sparse networks or *O*(*n*^4^) for all graphs, although 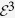 is a polynomial time algorithm, it can, in practice, only manage graphs that contain thousands of nodes. Therefore, it is difficult to detect the natural *K*-dimensional structure of a network of large sizes for *K* > 3 (the time complexity generally increases by a factor of *n*^2^ whenever the dimension increases by 1, for dimensions *K* ≥ 2), which poses a new issue regarding the design of better algorithms for minimising the *K*-dimensional structural entropy of networks for each *K* > 1, including for the case of *K* = 2. We remark that the time complexity 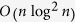 of 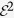 for networks is in fact impractical for *n* as large as hundreds of millions. For this reason, there is a need to design better algorithms to find the minimal two-dimensional structural entropy of networks.

Finally, we note that 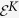 is a heuristic algorithm to compute the *K*-dimensional structural entropy of graphs and indicate that precisely computing the *K*-dimensional structural entropy of graphs is an extremely difficult problem that should be resolved in future computer science studies.

*Remark*: (i) the merging operator combines two sets *X* and *Y* into a set 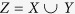 such that all of the nodes in *Z* are not distinguished and are allowed to re-group within *Z* in the future; (ii) the combining operator combines two sets *X* and *Y* into 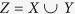 such that that the subtypes *X* and *Y* are kept within *Z*; (iii) the two operators are natural rules in real world clustering, which incorporates the idea of a mixture both bottom-up and top-down methods; (iv) our algorithm 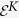 is a basic greedy strategy for minimising the *K*-dimensional structural entropy, and new rules are required to design new algorithms to minimise the *K*-dimensional structural entropy of graphs; (v) we determined the *K*-dimensional structural entropy for small values of *K* because, in real world networks, hierarchical structures occur; however, the number of levels of a community within a community is indeed small.

Clearly, our algorithm 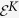 not only seeks to follow the principle of self-organisation of networks, but also the natural rules in the real world as the operators of the algorithm. The algorithms are designed to explore the natural two- and three-dimensional structures of networks rather than optimise an artificially defined object function. We use this strategy because natural objects can be identified by following natural rules, and algorithm 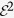 has been shown to successfully detect natural communities in social networks^17^. The algorithm 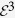 can be regarded as a deep detecting algorithm that seeks to explore the natural hierarchical structure of networks.

We use our algorithms 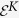 for *K* = 2, 3 to identify the modules and submodules of cancers and to compare our algorithms with the two most frequently used algorithms, namely, the modularity maximisation algorithm 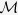
19 and the description length minimisation algorithm 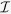
20.

## Constructing Cell Sample Networks Based on Gene Expression Profiles

Suppose that *v*_1_, *v*_2_, ···, *v*_*n*_ are *n* samples of cells and that *g*_1_, *g*_2_, ···, *g*_*N*_ are *N* genes. For every pair (*i*, *j*), let *a*(*i*, *j*) be the expression profile of gene *g*_*i*_ in sample *v*_*j*_. Then, for every *j* from 1 to *n*, a vector (*a*(1, *j*), *a*(2, *j*). ···, *a*(*N*, *j*)) occurs and represents the gene expression profiles of the sample *v*_*j*_, denoted *P*_*j*_. For every pair (*j*, *j*′), let *W*_*j*,*j*′_ be the Pearson correlation coefficient between *P*_*j*_ and *P*_*j*′_, the gene expression profiles of samples *v*_*j*_ and *v*_*j*′_, respectively.

A cell sample network *G* = (*V*, *E*) is constructed on the basis of the gene expression profiles by the following algorithm, denoted 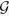.

Algorithm 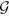 works with a fixed natural number *k*, and proceeds as follows:

1. The vertices of *G* are the cell samples *v*_1_, *v*_2_, ···, *v*_*n*_, that is, let *V* = {*v*_1_, *v*_2_, ···, *v*_*n*_}; and
2. For every *j*, suppose that *u*_1_, *u*_2_, ···, *u*_*k*_ are the cell samples such that *W*(*v*_*j*_, *u*_1_), *W*(*v*_*j*_, *u*_2_), ···, *W*(*v*_*j*_, *u*_*k*_) are the highest *k* weights among the weights *W*(*v*_*j*_, *u*) for all of the samples *u*, where *W*(*v*_*j*_, *u*) is the Pearson correlation coefficient between the gene expression profiles of samples *v*_*j*_ and *u*. For every *i* from 1 to *k*, create an edge (*v*_*j*_, *u*_*i*_) with weight *W*(*v*_*j*_, *u*_*i*_).

This constructs the weighted graph *G* = (*V*, *E*).

In the construction of *G*, *k* is a fixed number that depends on different cell samples and gene expression profiles.

The choice of *k* is a challenging problem. It requires that the choice of *k* ensures that the nontrivial weights are maintained in the generated graph, and the trivial or noisy weights are removed. Here, we realise the idea by the following algorithm, denoted 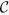.

Algorithm 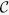 proceeds as follows:

1. (Noise amplifying) Fix a *noise amplifier σ*. Let *W* be the average wight among all the pairs of cell samples. Let *M* = *σ* ⋅ *W* be the modifier. Let *H* be the weighted graph of the cell samples such that for every pair (*i*, *j*) of cell samples, there is a weight *W*′(*i*, *j*) = *W*(*i*, *j*) + *M*. This step amplifies the noise for all the weights. The roles of this step are two-fold: if the weight *W*(*i*, *j*) between cell samples *i* and *j* is nontrivially high, then the modified weight *W*′(*i*, *j*) = *W*(*i*, *j*) + *M* is approximately the original weight *W*(*i*, *j*) since the modifier *M* is small, and if the weight *W*(*i*, *j*) is trivial or noisy, then the modified weight *W*′(*i*, *j*) = *W*(*i*, *j*) + *M* is significantly amplified, which allows our algorithm to better filter the noise or trivial weights from the highly nontrivial weights. In our experiments, we choose the noise amplifier 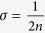 when *n* < 1000, and 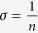 when *n* > 1000, where *n* is the number of the cell samples. This choice of *σ* is approximately equivalent to the following operation: Every cell sample increases a unit weight and uniformly assigns the extra unit weight to all the other cell samples. (The crucial new idea in algorithm 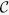 is the introduction of the noise amplifier *σ* and the modifier *M*. The motivation is to amplify noise for an algorithm to easily identify the noises. However, it may have more implications and have some theoretical backgrounds. The exact form of the *σ* may vary a bit for different networks.)
2. For every *k*, let *H*_*k*_ be the weighted graph obtained from *H* as follows:
  - The modifier *M* is kept for every edge.
  - For every cell sample *i*, keep the weighted edges of the top *k* weights, and delete all the other weights.
3. For each *k*, let *H*(*k*) be the one-dimensional structural entropy of the weighted graph *H*_*k*_. We say that *k* is a *stable point*, if both *H*(*k* − 1) > *H*(*k*) and *H*(*k* + 1) > *H*(*k*) hold.
4. (Minimisation of non-determinism or uncertainty) Define *k* to be the *k*′ that achieves the least one-dimensional structural entropy among all the stable points. That is, *k* is a stable point, and *H*(*k*) is the least among the *H*(*k*′) for all the stable points *k*′.

This step ensures that the chosen *k* generates a network structure with minimum uncertainty or non-determinism.

In this study, we consider a cell sample network for 4 cancers (additional cancer types and healthy tissue are discussed in the supplementary information) using the following data.

1. Acute leukaemia from Golub *et al*.^1^, which were obtained from acute leukaemia patients at the time of diagnosis. The data contain the expression of 7,129 genes for 38 samples that constitute 3 cell types. The three tumour types obtained from acute leukaemia patients at the time of diagnosis are as follows: 11 acute myeloid leukaemia (AML) samples; 8 T-lineage acute lymphoblastic leukaemia (ALL) samples; and 19 B-lineage ALL samples.
2. Lymphoma data from Alizadeh *et al*.^2^, which contain the expression of 4,026 genes for 96 samples, which constitute 9 cell types. The 9 types consist of three different types of tumours, diffuse large B cell lymphoma (DLBCL), chronic lymphocytic leukaemia (CLL), and follicular lymphoma (FL), as well as normal B and T cells at different stages of cell differentiation, including germinal centre B, NL.lymph node/tonsil, activated blood B, resting/activated T, transformed cell lines, and resting blood B. On the basis of gene expression profiling, Alizadeh *et al*.^2^ have suggested dividing the DLBCL type into two subtypes, GC B-like DLBCL and activated B-like DLBCL.
3. Multi-tissues from the multi-tissue dataset from Ramaswamy *et al*.^3^, which contain the expression of 5,565 genes for 103 samples constituting 4 cell types. The tissue samples are from four distinct cancer types: 26 breast, 26 prostate, 28 lung, and 23 colon samples.
4. The test data of leukemia from Haferlach *et al*.^10^, which contains 1152 samples and 1480 gene expression profiles. The samples consist of 18 subtypes.

**Figure 1:**
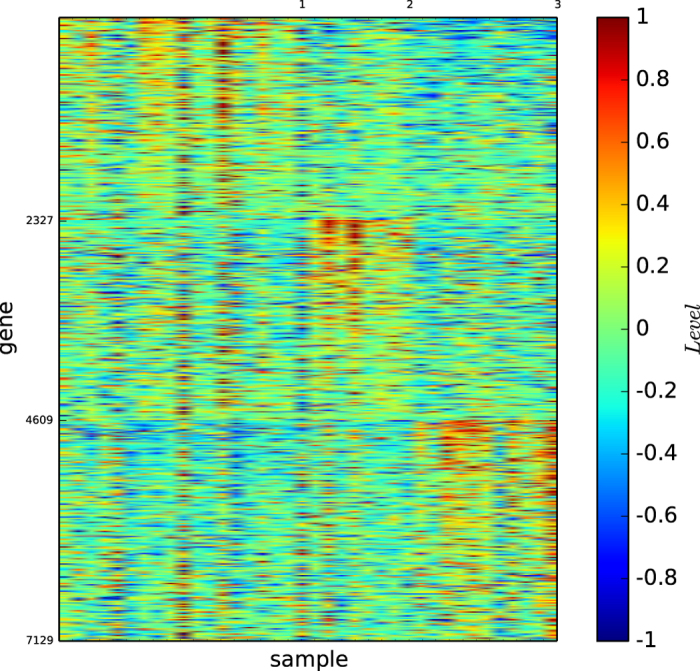
Gene map of the true types of acute leukaemia. The three types are the ALL-B, ALL-T and AML, respectively.

**Figure 2:**
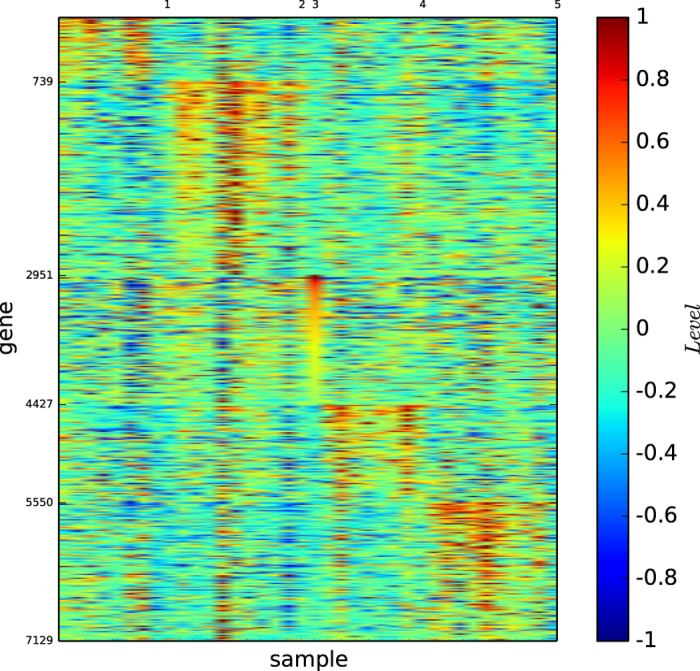
Gene map of the modules of acute leukaemia identified by 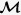. The 5 modules and their modules are provided in the supplementary information.

**Figure 3:**
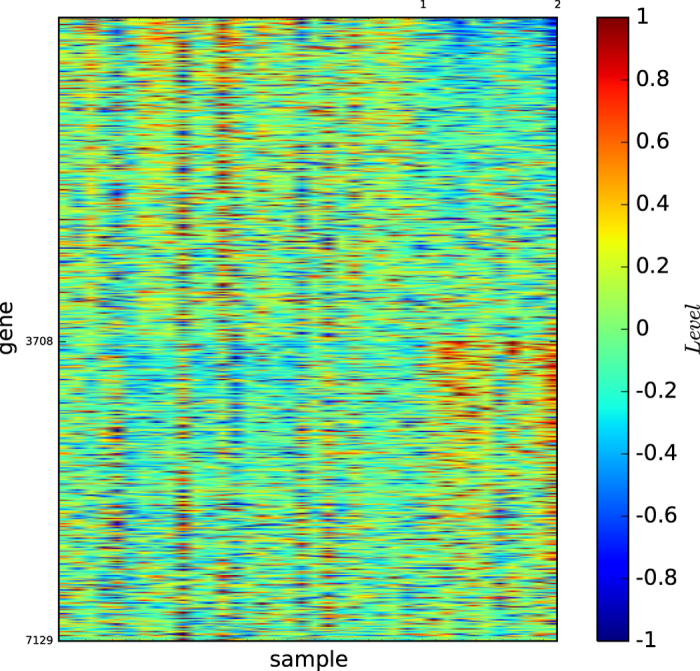
Gene map of the modules of acute leukaemia identified by 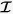. The 2 modules and their modules are provided in the supplementary information.

**Figure 4:**
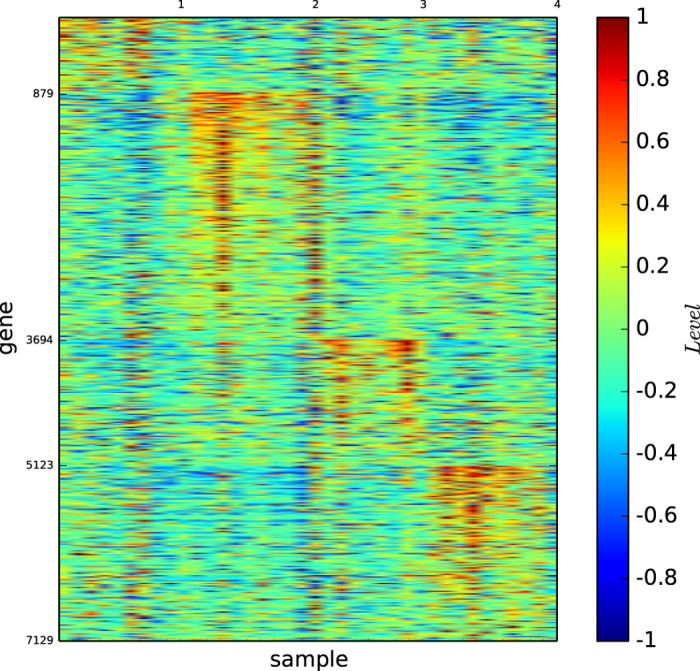
Gene map of the modules of acute leukaemia identified by 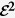. The 4 modules and their modules are provided in the supplementary information.

In Figures 1, 2, 3 and 4 of the supplementary information, denoted S-Figures 1, 2, 3 and 4, we depict the curves of the one-dimensional structural entropy function *H*(*k*) for the acute leukaemia, lymphoma, the multi-tissues and the new test leukemia data, respectively.

By observing S-Figures 1, 2, 3 and 4, we have that the parameter *k* chosen by the algorithm 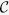 for the acute leukaemia, the lymphoma, the multi-tissues and the new test leukemia data are *k* = 7, *k* = 6, *k* = 9, and 11, respectively. The algorithm 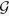 with the chosen *k* = 7, 6, 9 and 11, constructs the cell sample networks of the acute leukaemia, the lymphoma, the multi-tissues and the test data leukemia, respectively.

## Gene Classification Map

For a cancer with cell samples *V* = {*v*_1_, *v*_2_, ···, *v*_*n*_}, suppose that *g*_1_, *g*_2_, ···, *g*_*N*_ are all of the genes. For a given gene *g* = *g*_*i*_, we use *g*(*v*) to denote the gene expression profile of *g* for cell sample *v* ∈ *V*. By normalising, we ensure that the average *g*(*v*) for all values of *v* is 0, and the values *g*(*v*) for all samples *v*, are real numbers in [−1, 1] (details of the normalisation is provided in the Methods section).

Let *G* = (*V*, *E*) be the cell sample graph of a cancer based on the gene expression profiles. Suppose that {*X*_1_, *X*_2_, ···, *X*_*L*_} is a partition of *V* identified by a community-detecting algorithm from the graph *G*. For a set *X*_*i*_, we define *g*(*X*_*i*_) to be the average of *g*(*x*) for all *x* ∈ *X*_*i*_. For a gene *g*, we use *X*_*g*_ to denote the set *X*_*i*_ such that *g*(*X*_*i*_) = max_*j*_{*g*(*X*_*j*_)}.

For every *j*, we define *B*_*j*_ to be the set of all genes *g* such that *X*_*g*_ = *X*_*j*_, which are listed in decreasing order of the gene expression value.

A gene map of *G* according to the partition {*X*_1_, *X*_2_, ···, *X*_*L*_} is a matrix of colour codes of *L* × *L* blocks. In the matrix, the *j*-th row is the set *B*_*j*_ ordered as defined above, and the *j*-th column is the set *X*_*j*_ listed in a fixed order. All of the genes are listed in the ordering *B*_1_, *B*_2_, ···, *B*_*L*_, in which each *B*_*j*_ has its own order by the gene expression profiles.

The gene map explores the cell types and the subtypes of the cancer tumours and the set of genes that determines the corresponding cell types and subtypes.

Next, we prepare to construct our gene map method and define the cancer types and subtypes.

Before implementing the experiments, we summarise the steps of our approach. Our approach for constructing the gene map consists of the following steps:

1. Construct a weighted network of cell samples on the basis of the gene expression profiles of the cell samples;
2. Identify the modules and/or submodules by community identification algorithms for networks;
3. Identify the set of genes that defines the modules and submodules of the cell sample network identified by the algorithms;
4. Compare the true tumours types with the modules and submodules identified by the community identification algorithms; and
5. Analyse the survival times, survival indicators and IPI scores of the modules and submodules of the cancers identified by all of the algorithms.

Our approach differs from the hierarchical clustering methods, and various learning algorithms. Our method directly defines types and subtypes of cancers by network algorithms, and is completely different from the learning algorithms that find a classifier based on some training samples and assign a new sample to a known type using the classifier.

A cell sample network is constructed on the basis of the minimisation of one-dimensional structural entropy as realised in algorithms 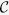 and 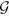.

We define the types and subtypes of cell samples from all of the graphs above by our algorithms based on the *K*-dimensional structural entropy of graphs, which is denoted 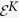, for *K* = 2 and 3, and by the 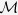 and 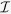 algorithms, respectively.

The details on the modules identified by the algorithms are reported in the supplementary information. All of the experiments and analyses performed for the additional cancer, the lung cancer and healthy tissue are given in the supplementary information.

## Similarity of the Modules Identified by an Algorithm to the True Tumour Types

For a network *G* = (*V*, *E*), let *X* and *Y* be two subsets of *V*. We define the *similarity of Y to X* as follows:


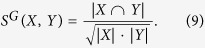


Suppose that 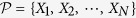 and 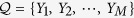 are two partitions of *G*.

Then, *similarity of* 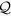 *to* 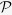 is defined as the function 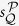 such that for all *j* ∈ {1, 2, ···, *N*},


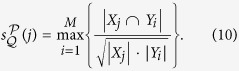


## Acute Leukaemia

### Similarity

Table 1 describes the similarity of the modules of acute leukaemia identified by the algorithms 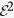, 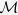, 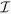 and 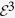.

According to Table 1, the similarity of the modules identified by 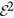 is the same as that identified by 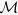, the weighted average similarity of the modules found by 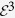 is less than that by the algorithm 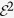 and larger than that by 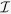, and the average weighted similarity found by 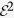 is the same as that found by 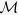, whereas it is higher that that found by 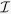. According to the similarities listed in Table 1, 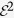, 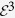 and 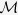 all perform better than 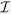 in this case.

According to S-Tables 1, 2, 3, 4 and 6, the tables in the supplementary information, we observe the following results: (1) There are 3 true types. (2) Algorithm 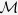 found 5 models. (3) Algorithm 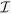 found 2 modules. (4) Algorithm 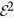 found 4 modules. (5) Algorithm 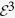 found 6 modules in which ALL_21302B-cell and ALL_7092_B-cell are singletons.

### Gene map of true types

Figure 1 shows the colour codes of the gene map of the three acute leukaemia true types: ALL-B, ALL-T and AML. Figure 1 reveals that the type ALL-B may not be a precise or refined tumour type of acute leukaemia, although the three types ALL-B, ALL-T, and AML are distinguishable by three different gene expression patterns.

### Gene map of the modules based on modularity maximisation

Figure 2 shows the gene map of the modules of acute leukaemia identified by the modularity maximisation algorithm 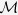. Figure 2 and S-Table 2 reveal that ALL-B is principally divided by modules 1 and 2, and modules 3 and 4 are basically the ALL-T and AML, and each of the modules is defined by a set of genes.

### Gene map by InforMap

Figure 3 shows the gene map of the modules of acute leukaemia identified by the algorithm InforMap 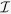. Figure 3 and S-Table 3 show that algorithm 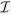 identified only two modules and none of the identified modules is clearly defined by a unique set of genes or is highly similar to a true type.

### Gene map of the modules by structural entropy minimisation 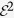


Figure 4 depicts the gene map of the modules of the cell sample network of acute leukaemia identified by our algorithm 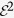.

Figure 4 and S-Table 4 show the following results: (1) Module 1 is a subtype of ALL-B (except for AML_13), and it is defined by a set of more than 800 genes. (2) Module 2 is a subtype of ALL-B, and it is defined by a set of more than 2,500 genes. (3) Module 3 is exactly the type ALL-T, and is defined by a set of more than 1,000 genes. (4) Module 4 is the type AML (missing AML_13), and is defined by a set of more than 1,000 genes.

These results demonstrate that both modules 1 and 2 are biologically meaningful subtypes of the type ALL-B except for AML_13.

According to Figs 1 and 4, the true types and the modules identified by our algorithm 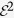 are distinguishable. However, the pictures are not highly-defined because, the gene expression profiles in the diagonal blocks are not extremely high and the gene expression profiles other than the diagonal blocks are nontrivially high.

### Three-dimensional gene map

**Figure 5:**
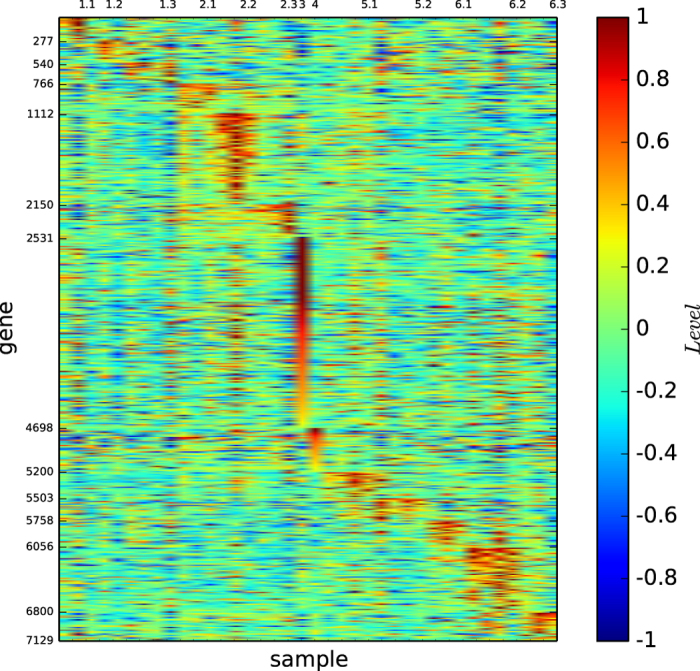
Gene map of the modules of acute leukaemia identified by 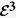. The 6 modules and their submodules are provided in the supplementary information.

Figure 5 depicts the gene map of the refined classification of acute leukaemia provided by our algorithm 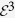.

Figure 5 and S-Table 6 reveal the following results:

1. Algorithm 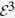 identified 6 modules. Each module *X*_*i*_ is either a true type or a subset of a true type that consists of several distinguishable submodules and indicate that all of the modules are distinguishable and each submodule *Y*_*i*,*j*_ is uniquely determined by a block of genes *B*_*i*,*j*_, which generates a high-definition and one-to-one map from the submodules *Y*_*i*,*j*_ to gene expression patterns *B*_*i*,*j*_ for all *i* and *j*.
2. Submodule 2.2 is defined by a set of more than 1,000 genes.
3. Module 3 is defined by a set of more than 2,000 genes.
4. Module 4 is defined by a set of more than 400 genes.
5. Submodule 6.2 is defined by a set of more than 700 genes.

It is conceivable that an analysis of gene set *B* that defines a module *X* or a submodule *Y* may be required to treat the corresponding module *X* or submodule *Y*. However, our results show that a module *X* or a submodule *Y* usually has a large gene set *B* that expresses the module *X* or submodule *Y*, which could lead to difficulty in treating a tumour type. To address this issue, our three-dimensional gene map analysis suggests that for a tumour type *X*, we may divide *X* into submodules *Y*_1_, *Y*_2_ and *Y*_3_. Upon analysis, the gene sets *B*_1_, *B*_2_ and *B*_3_ may express *Y*_1_, *Y*_2_ and *Y*_3_, respectively. This analysis could aid in treating the tumour type *X*. Nevertheless, we believe that it is fundamental to identify and analyse the large set of genes that express a biologically meaningful module or submodule, and our three-dimensional cancer gene map can provide this method.

The results (1) to (5) demonstrate that all of the modules and submodules classified by our algorithm 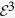 maybe biologically meaningful. In particular, according to (3) and (4), ALL_21302_B-cell and ALL_7092_B-cell are remarkably cells that may play essential roles in the classification, diagnosis and therapy of acute leukaemia. According to (2) and (5), submodules 2.2 and 6.2 could be extremely important in the classification, diagnosis and therapy of acute leukaemia.

*Remark*. All the algorithms 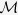, 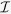, 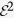 and 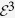 fail to correctly assign the cell sample AML_13 to the AML type. This finding implies that AML_13 could be particularly interesting.

## Gene Map of Lymphoma

### Similarity

Table 2 shows the similarity of the modules of the lymphoma identified by 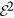, 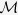, 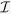 and 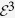 to the true types.

According to S-Tables 8, 9, 10, 11 and 14, we have the following results: (1) There are 9 true types. (2) Algorithm 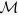 found 4 modules. (3) Algorithm 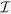 found 9 modules. (4) Algorithm 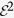 found 11 modules. (5) Algorithm 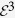 found 13 modules.

Table 2 reveals the following results: (1) 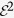 exactly identifies 4 true types. (2) 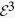 exactly identifies 4 true types. (3) 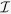 exactly identifies 2 true types. (4) 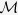 exactly identifies only one true type.

The results above demonstrate that the modules found by 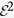 and 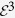 may be the best. In fact, dividing DLBCL according to the algorithms 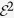 and 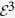 defines the prognostic categories. This example also indicates that weighted similarities are insufficient for evaluating the quality of the modules for detecting algorithms because the true cancer types may not be absolutely correct (otherwise, these cancers may have already been resolved).

### Gene map of true types

**Figure 6:**
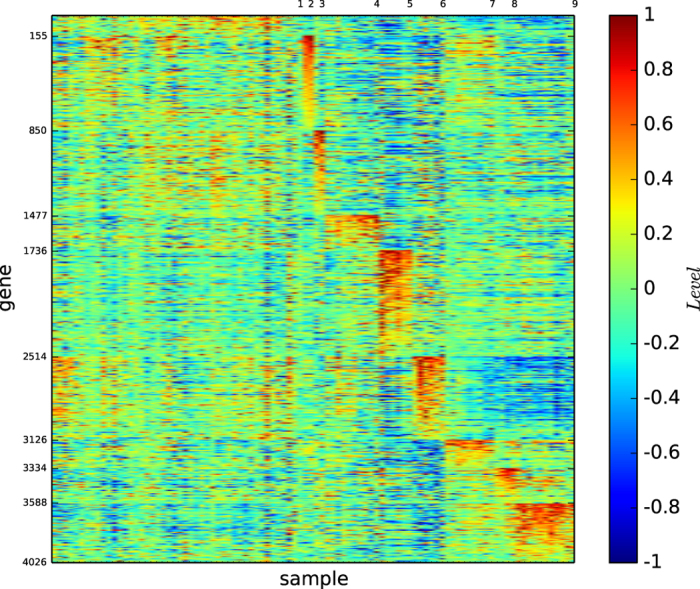
Gene map of the true types of lymphoma. The nine types are: 1 DLBCL, 2 Germinal centre B, 3 Nl. lymph node/tonsil, 4 Activated blood B, 5 Resting/activated T, 6 Transformed cell lines, 7 FL, 8 Resting blood B, and 9 CLL.

Figure 6 shows the gene map of the true types of lymphoma, which consists of 9 types: DLBCL, germinal centre B, NL. lymph node/tonsil, activated blood B, resting/activated T, transformed cell lines, FL, resting blood B, and CLL, which are ordered as listed.

Figure 6 reveals the following results:1) All of the types are distinguishable because they are defined by different blocks of genes. 2) All of the types except DLBCL are expressed by different sets of genes. 3) The type DLBCL is a large set; however, it is not well-expressed. 4) Four types (germinal centre B, NL. lymph node/tonsil, resting/activated T and transformed cell lines) are highly expressed by a set of many genes; thus, the blocks of genes expressing the types are large. 5) Except for DLBCL, the 8 remaining types are highly expressed by their corresponding blocks of genes.

These results imply that DLBCL is not a well-defined type, which will be shown in the gene map of the modules of lymphoma identified by the algorithm 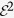.

### Gene map of the modules by modularity maximisation

**Figure 7:**
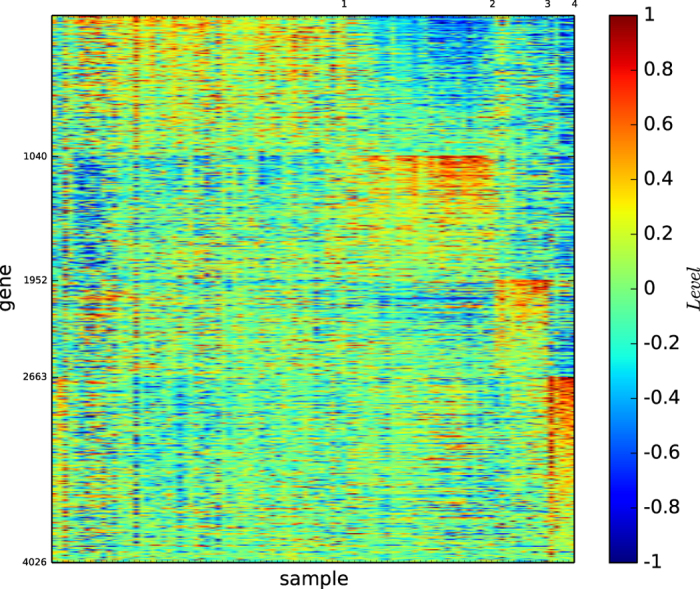
Gene map of the modules of lymphoma identified by 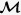. The 4 modules and their modules are provided in the supplementary information.

Figure 7 depicts the gene map of the modules of lymphoma identified by the modularity maximisation algorithm 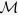.

Figure 7 and S-Table 9 reveal the following properties: (1) Algorithm 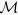 identified 4 modules. (2) The four modules identified by 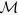 are distinguishable by different sets of genes.

(1) indicates that the modules identified by 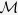 are far from the true types. (1) and (2) imply that gene expression patterns alone is insufficient for evaluating the modules identified by a community detection algorithm.

### Gene map by InforMap

**Figure 8:**
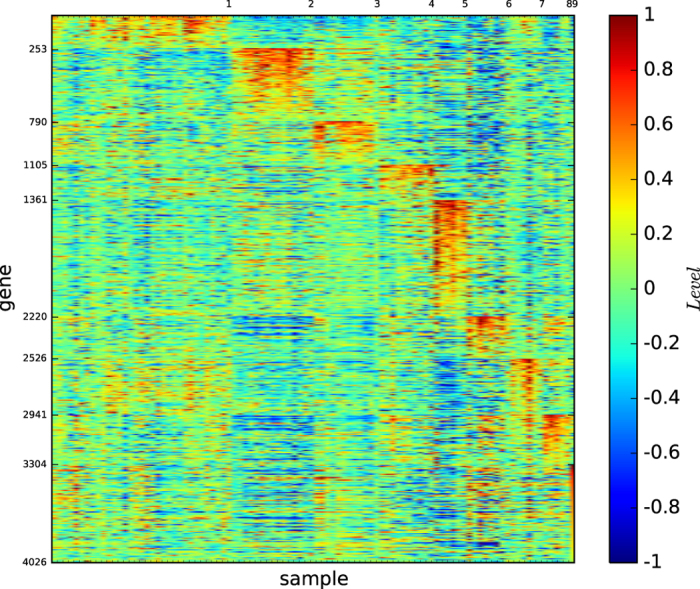
Gene map of the modules of lymphoma identified by 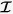. The 9 modules and their modules are provided in the supplementary information.

Figure 8 depicts the gene map of lymphoma classified by algorithm 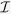.

Figure 8 and S-Table 10 reveal the following results: (1) 9 modules are found. (2) Each of the 9 modules is defined by a unique set of genes. (3) The large DLBCL type is divided, and the DLBCL samples are assigned to 6 modules.

(3) is interesting. As we mentioned before, the DLBCL type is too large, and may not be a well-defined true type. Here, we see that the algorithm 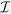 assigned the DLBCL cell samples to 6 modules. We will further analyse the divisions of the DLBCL samples using clinical data.

### Gene map of the modules by structural entropy minimisation algorithm 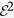


Our algorithm 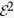 identified 11 modules for lymphoma, and the results are provided in the supplementary information.

**Figure 9:**
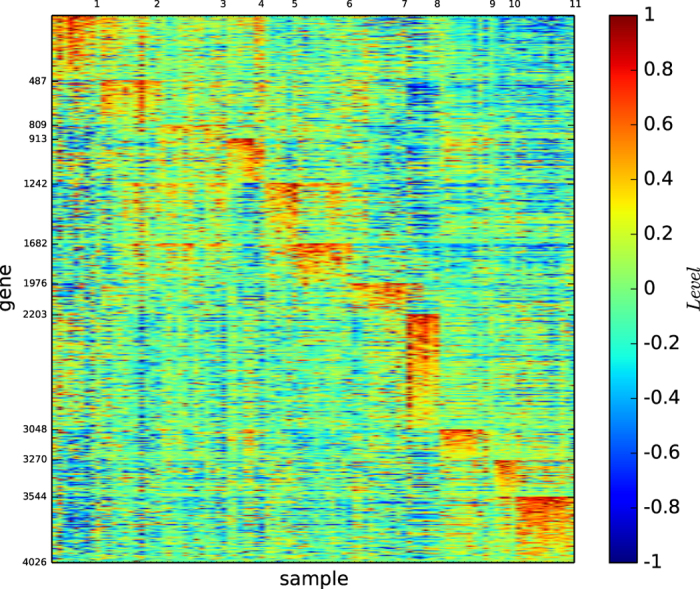
Gene map of the modules of lymphoma identified by 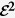. The 11 modules and their modules are provided in the supplementary information.

Figure 9 depicts the gene map of lymphoma classified by our algorithm 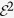.

Figure 9 and S-Table 11 reveal the following properties: (1) Modules 1, 2, 3, 7, 8, 9, 10, and 11 essentially correspond to transformed cell lines, activated B-like DLBCL, GC B-like DLBCL, activated blood B, resting/activated T, FL, resting blood B, and CLL, respectively. (2) The DLBCL type is essentially divided into modules 2, 3, and 6. (3) Except for module 3, which contains the subtype GC B-like DLBCL, every module is highly expressed by a significantly large set of genes. (4) Module 2 is the subtype activated B-like DLBCL and is highly expressed by a set of more than 300 genes. (5) Module 3 contains the subtype GC B-like DLBCL (except for DLCL-0011) and is large. However, the module is well-expressed only by a set of less than 100 genes. This finding could be caused by i) the expression of the subtype GC B-like by only a small set of genes or ii) an incomplete current gene expression array. (6) Module 6 contains a subset of DLBCL, and its biological and medical classification is unknown. However, our two-dimensional gene map shows that the module is highly expressed by a set of more than 290 genes. (7) Module 4 is a combination of GC B-like DLBCL, and germinal centre B. Our gene map shows that the module is highly expressed by a set of more than 300 genes. (8) Module 5 is a combination of activated B-like DLBCL and NL. lymph node/tonsil. Our gene map shows that the module is highly expressed by a set of more than 400 genes, implying that, it is a biologically meaningful type. (9) Module 8 is the resting/activated T, and it is highly expressed by a set of more than 1,800 genes. (10) Module 11 is the CLL, and it is highly expressed by a set of more than 450 genes.

These results imply that modules 2, 3 and 6 could be new subtypes of DLBCL and modules 4 and 5 could be new subtypes of lymphoma. We will verify these results by clinical data analyses.

### Three-dimensional gene map

**Figure 10:**
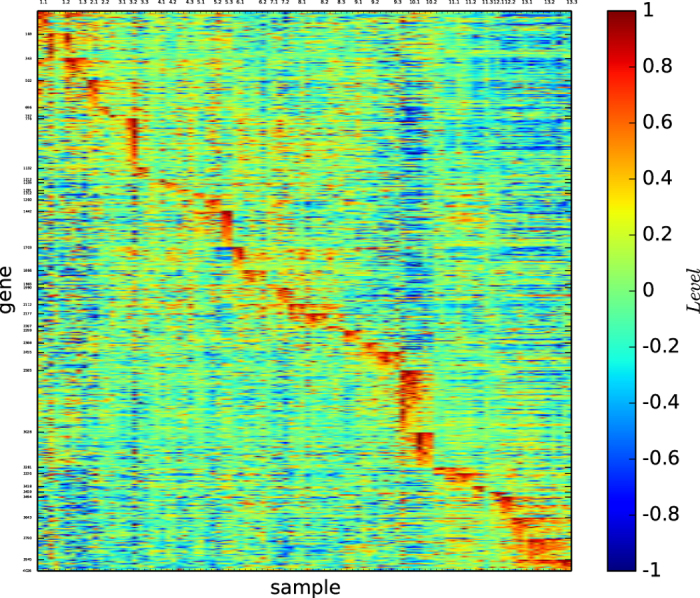
Gene map of the modules of lymphoma identified by 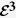. The 13 modules and their submodules are provided in the supplementary information.

Figure 10 depicts the gene map of the refined classification of lymphoma derived by our algorithm 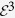, for which the details of modules and submodules are provided in the supplementary information. Figure 10 and S-Table 12 establish the three-dimensional gene map from the types and subtypes of the gene expression patterns, which shows the types and subtypes of lymphoma, and demonstrate that almost all of the subtypes may have a biological meaning related to the classification of tumour types and subtypes of lymphoma. In particular, it predicts some remarkable subtypes for DLBCL and lymphoma, which will be verified by clinical data analyses.

*Remark*: Interestingly, all of the algorithms isolate DLCL-0009 from the other DLBCL samples, although the reason remains unclear.

## Gene Map of Multi-tissues

### Similarity

Table 3 describes the similarity of the modules of the multi-tissues identified by the algorithms 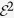, 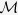, 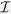 and 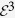. Table 3 shows the following results: (1) Algorithm 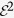 exactly identifies 1 true type and approximates the other three true types with similarities greater than 0.94. (2) Algorithm 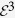 exactly identifies 1 true type and approximates the other three true types with similarities greater than 0.91. (3) Algorithm 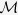 identifies 1 true type and approximates the other three true types with similarities greater than 0.85. (4) Algorithm 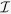 approximates all the true types with similarities greater than 0.76.

According to S-Tables 17, 18, 19, 20 and 22, we have the following results: (1) There are 4 true types. (2) 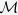 found 5 modules. (3) 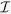 found 6 modules. (4) 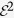 found 4 modules. (5) 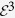 found 7 modules, including 3 singletons, BR_U16, BR_UX7 and LU_A17T.

### Gene map of true types

**Figure 11:**
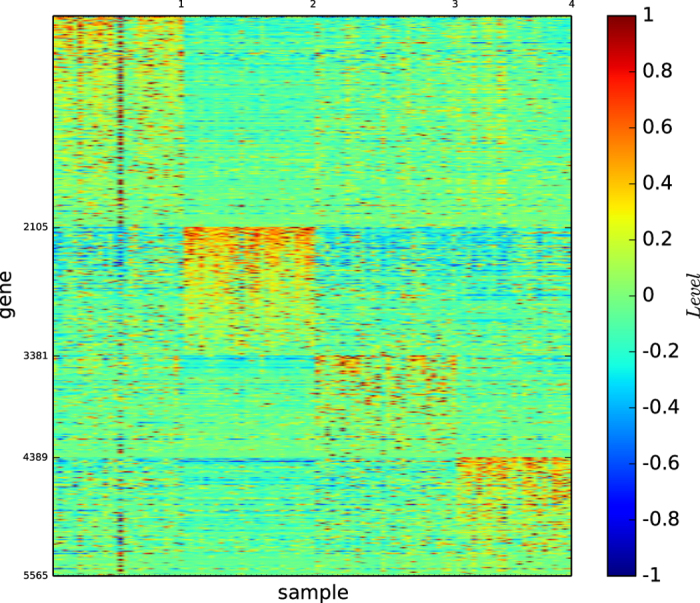
Gene map of the true types of multi-tissues. The types 1, 2, 3 and 4 are the BR, PR, LU and CO, respectively.

Figure 11 depicts the gene map of the true types of the multi-tissues ordered according to the types BR, PR, LU and CO.

As shown in Fig. 11, each of the types is well-expressed by the gene expression map and all of the types are distinguishable.

### Gene map of the classification by modularity maximisation

**Figure 12:**
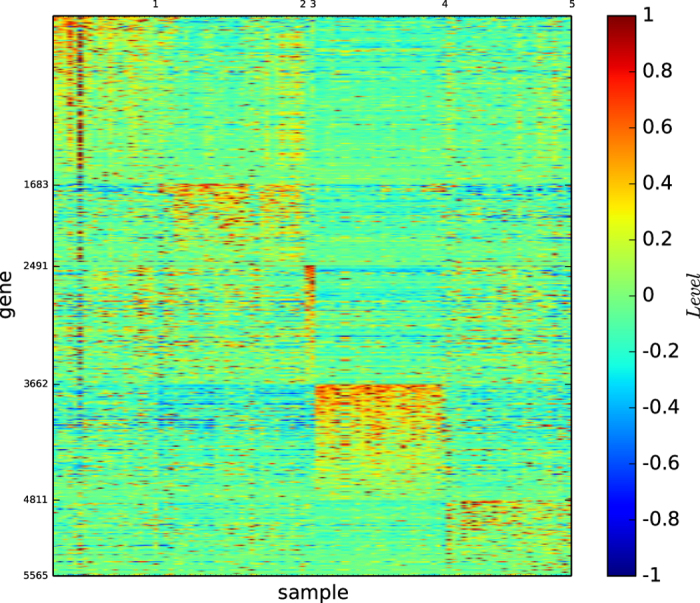
Gene map of the modules of multi-tissues identified by 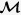. The 5 modules and their modules are provided in the supplementary information.

Figure 12 depicts the gene map of the modules of the multi-tissues identified by the modularity maximisation algorithm 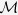.

Figure 12 and S-Table 18 reveal the following results: (1) 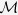 identified 5 modules, and one is a true type. (2) Except for the true type, the other 4 modules are not well-defined by a distinct set of genes.

### Gene map by InforMap

**Figure 13:**
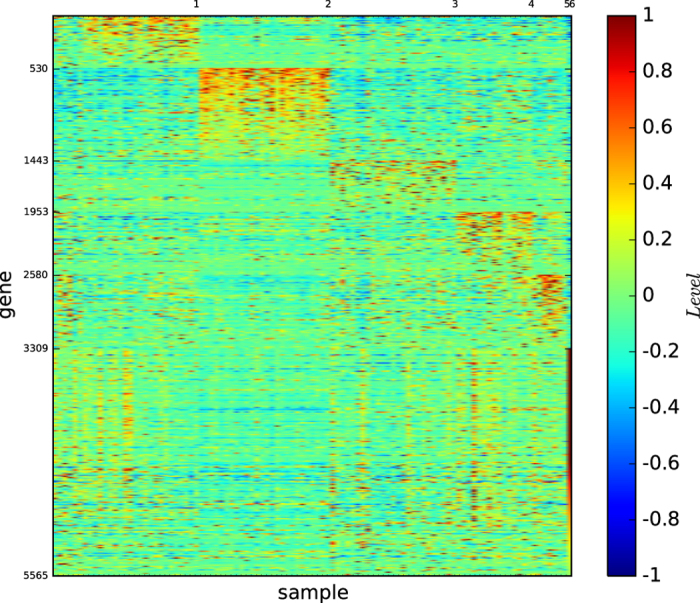
Gene map of the modules of multi-tissues identified by 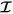. The 6 modules and their modules are provided in the supplementary information.

Figure 13 depicts the gene map of the multi-tissue dataset according to algorithm 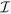.

Figure 13 and S-Table 19 reveal the following results: (1) 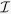 identified 6 modules, and one is a true type. (2) Each module is well-defined by a distinct set of genes. (3) The 6th module contains only one sample, BR_U16.

### Gene map of the multi-tissues by structural entropy minimisation algorithm 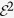


**Figure 14:**
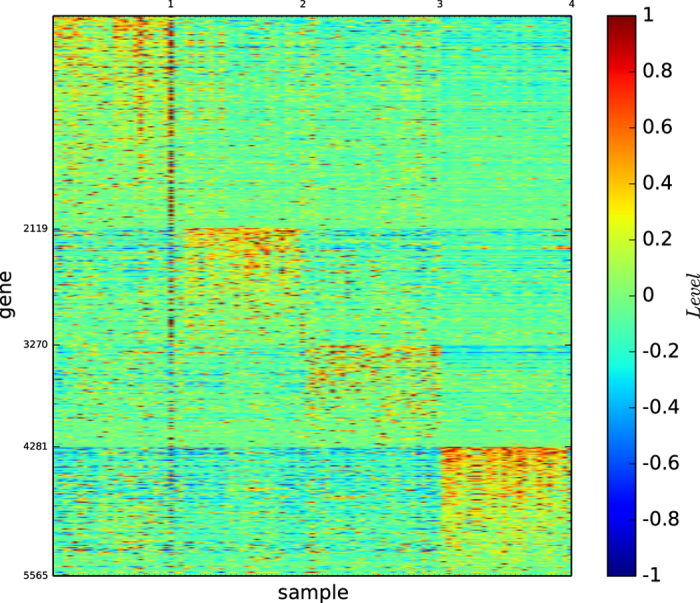
Gene map of the modules of multi-tissues identified by 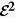. The 4 modules are exactly the same or almost the BR, CO, LU and PR respectively, for which the details are provided in the supplementary information.

Figure 14 depicts the gene map of the multi-tissue dataset provided by our algorithm 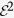. Figure 14 and S-Table 20 reveal that the modules 1–4 here are the same as or highly similar to BR, CO, LU and PR, respectively.

### Three-dimensional gene map

**Figure 15:**
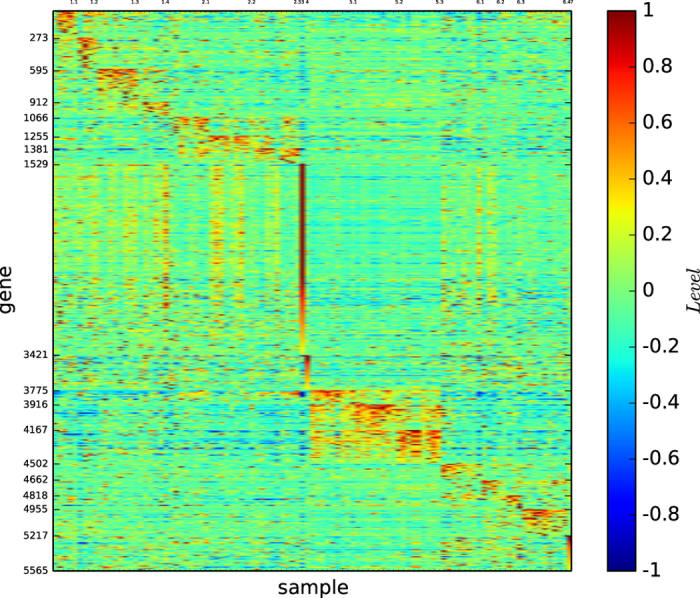
Gene map of the modules of multi-tissues identified by 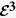. The 7 modules and their submodules are described in the supplementary information.

Figure 15 depicts the gene map of the refined module and submodule classification of the multi-tissue dataset according to our algorithm 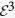.

Figure 15 and S-Table 21 reveal the following results: (1) Module 1 is essentially of the type BR. (2) Module 2 is of the type CO and consists of 3 distinguishable submodules. (3) Modules 3, 4 and 7 are singletons and consist of BR_U16, BR_UX7 and LU_A_LU_A17T, respectively. (4) Module 5 is of the PR type and consists of 3 distinguishable submodules. (5) Module 6 is of the type LU and consists of 4 distinguishable submodules. (6) Each submodule is defined by a set of a significantly large number of genes. (7) Every gene pattern defining a submodule fails to define any other module or submodule. (8) The three cells, BR_U16, BR_UX7 and LU_A_LU_A17T are expressed by sets of more than 1,500, 300 and 300 genes, respectively.

These results demonstrate that our three-dimensional gene map shown in Fig. 15 provides a high-definition, one-to-one map from the submodules of true cell types to the gene expression patterns of the multi-tissues and indicates that BR_U16, BR_UX7 and LU_A_LU_A17T are remarkable cells that may play special roles in the classification of multi-tissues.

## DLBCL Submodules Identified by 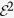 and 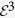 Define Prognostic Categories

We used the DLBCL clinical data from Alizadeh *et al*.^2^ to analyse the overall survival times, survival ratios and IPI scores of the submodules of the DLBCL type identified by the algorithms 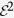, 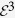, 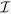 and 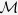.

### Submodules identified by 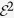


S-Tables 44 and 45 show the statistical survival times, survival ratios and IPI scores of the DLBCL submodules identified by the 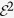 algorithm.

S-Tables 44 and 45 reveal the following results:

1. Except for DLCL_0041 and DLCL_0009, the DLBCL samples are divided into modules 2 to 6.
2. Module 2 essentially represents activated B-like DLBCL samples. The overall survival time is 26.4 months, the overall survival ratio is 20%, and the IPI scores are 2 or 3.
3. Module 3 contains 12 samples. The module includes GC B-like DLBCL samples. The overall survival time is 49.6 months, the survival ratio is 55%, and the IPI scores are 3 or 4.
4. Module 4 contains 4 GC B-like DLBCL samples. The overall survival time is 85.7 months, the survival ratio is 100%, and the IPI scores are 0 or 1.
5. Module 5 contains 4 activated B-like DLBCL samples. The overall survival time is 30 months, the survival ratio is 25%, and the IPI scores are 2 or 3.
6. For modules 2 to 5, most DLBCL samples within the same module share similar survival times, survival indicators and IPI scores.
7. Module 6 contains 10 samples. The overall survival time is 37 months, the survival ratio is 40%, and the IPI scores range from 0 to 4.
8. The DLBCL samples in distinct modules among modules 2 to 6 have different overall survival times, survival ratios and IPI scores.

These results demonstrate that most of the DLBCL samples divided in each of the modules 2–5 share similar survival times, survival indicators and IPI scores, indicate that the samples in different modules have significantly different overall survival times, survival ratios, and IPI scores, and show that the samples in module 6 have divergent survival times, survival indicators and IPI scores. These results indicate that the classification of the DLBCL samples in modules 2 to 5 are interpretable and distinguishable in clinical practice. However, module 6 is not well-defined in clinical practice.

### Submodules identified by 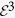


S-Tables 46 and 47 show the statistical survival times, survival ratios and IPI scores of the DLBCL submodules identified by 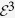.

S-Tables 11 and 14 show that 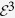 refines the modules identified by 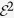. Therefore, the submodules of lymphoma identified by 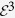 correspond to the submodules of the modules identified by 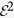. In particular, the DLBCL samples are divided into a number of submodules by 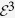.

S-Tables 46 and 47 reveal the following results:

1. The DLBCL samples in each of the submodules 2.2, 3.1, 3.3, 4.1, 4.3, 5.1, 6.1, 6.2 and 8.1 are similar to one another in survival times, survival indicators and IPI scores.
2. However, the DLBCL samples in submodules 3.2, 7.1, 7.2, 8.2 and 8.3 are divergent in survival times, survival indicators and IPI scores.
3. The overall survival times, survival ratios and IPI scores in most of the submodules are distinguishable.

Therefore, many of the submodules of the DLBCL samples identified by 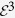 are interpretable by the similarity of survival times, survival indicators and IPI scores for the cell samples within the same submodule, and distinguishable by overall survival times, survival ratios and IPI scores for different submodules.

### Submodules identified by 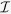 and 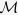


S-Tables 48 and 49 describe the statistical survival times, survival ratios and IPI scores of the DLBCL submodules identified by 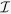.

S-Tables 48 and 49 reveal the following results:

1. Except for DLCL_0041 and DLCL_0009, the DLBCL samples are divided into modules 1, 7, and 8.
2. Module 1 contains 32 DLBCL samples. The overall survival time is 47.58 months, the survival ratio is 52%, and the IPI scores range from 0 to 4.
3. Module 7 contains 6 DLBCL samples. The overall survival time is 32.68, the survival ratio is 33%, and the IPI scores are 2 or 3.
4. Module 8 contains 3 DLBCL samples. The overall survival time is 20.07 months, the survival ratio is 0%, and the IPI scores are 1, 2 and 3.

Therefore, except for module 7, the DLBCL modules identified by 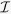 are non-interpretable and undistinguishable in clinical practice.

S-Table 50 describes the statistical survival times, survival ratios and IPI scores of the DLBCL submodules identified by 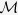.

S-Table 50 shows that the algorithm 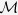 fails to identify submodules for the DLBCL samples.

Our results can be summarised as follows:

- Algorithm 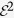 divides the DLBCL samples into several modules, and for almost all of the modules, most of the samples in the same module share similarity in survival times, survival indicators, and IPI scores, and the samples in different modules exhibit distinct overall survival times, survival ratios and IPI scores.
- Algorithm 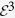 further refines the modules identified by 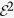 into submodules, and for most submodules, the samples in the same submodule share similar survival times, survival indicators, and IPI scores, and the samples in different submodules are distinguishable by the overall survival times, survival ratios and IPI scores.

The results above are better than expected. Surprisingly, our algorithms are derived from pure mathematics, and we did not use any information from biology or concepts from learning theory. The results may provide new insights into cancer study and therapies. For example, our results may indicate that a tumour type in different stages may correspond to different subtypes that have different gene expression patterns and are in different prognostic categories. Furthermore, our theory and algorithms have potential applications in a wide range of fields, including computer science, networking and data processing.

However, the algorithms 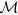 and 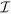 fail to divide the DLBCL samples into clinically meaningful subtypes.

In summary, in the networks constructed from the gene expression profiles on the basis of the one-dimensional structural entropy minimisation, the algorithms 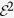 and 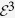 on the basis of the two- and three-dimensional structural entropy minimisation may identify the subtypes of tumours that are clinically interpretable and distinguishable. The results demonstrate that structural entropy minimisation, including the one-, two- and three-dimensional cases, could be the correct principle for defining the natural modules in nature, such as for defining cell types and subtypes of cancers.

## Gene Map of New Data Leukemia

### Similarity

Tables 4 and 5 describe the similarity of the modules of the new data Leukemia identified by the algorithms 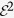, 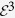, 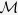 and 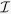.

Tables 4 and 5 show the following results: (1) Algorithm 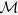 finds 9 modules which approximate 6 true types with similarity greater than 0.8. (2) Algorithm 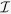 finds 27 modules which approximate 7 true types with similarity greater than 0.8. (3) Algorithm 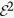 finds 17 modules which approximate 8 true types with similarity greater than 0.8. (4) Algorithm 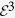 finds 18 modules which approximate 7 true types with similarity greater than 0.8.

According to S-Tables 55–91, we observe the following results: (1) There are 18 true types. (2) Algorithm 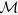 found 9 modules. (3) Algorithm 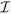 found 27 modules. (4) Algorithm 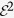 found 17 modules. (5) Algorithm 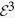 found 18 modules.

(1–5) demonstrate that our algorithms 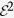 and 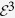 not only approximate well the true types, but also give the correct number of subtypes. However, the algorithms 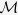 and 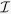 cannot even estimate the correct number of true types when the number of cell samples is large.

### Gene map of true types

**Figure 16:**
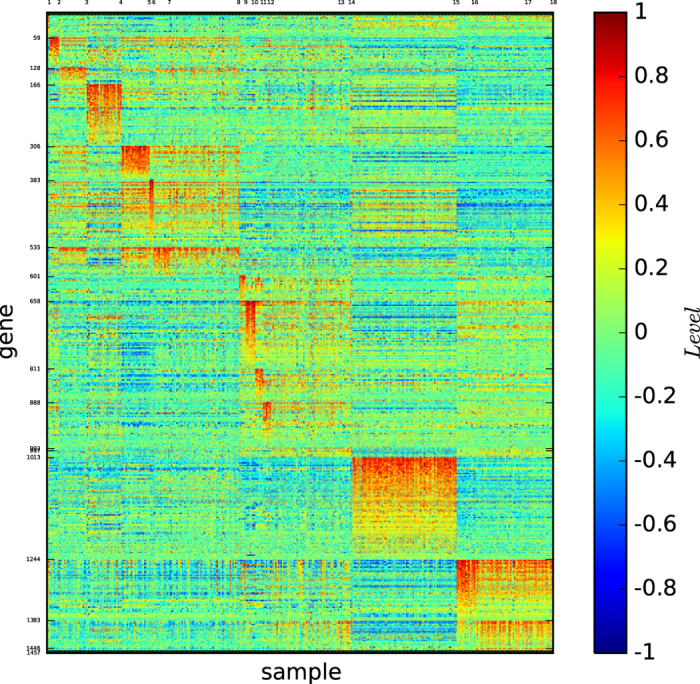
Gene map of the true types of new test leukemia data. There are 18 subtypes of the cell sample network.

Figure 16 depicts the gene map of the true types of the new data Leukemia.

Figure 16 shows that all the 18 subtypes are distinguishable, that C1, C3, C8, C13 and C18 are not well-defined by the corresponding gene patterns, and that all the other subtypes are well-defined by a unique gene pattern.

### Gene map of the classification by modularity maximisation

**Figure 17:**
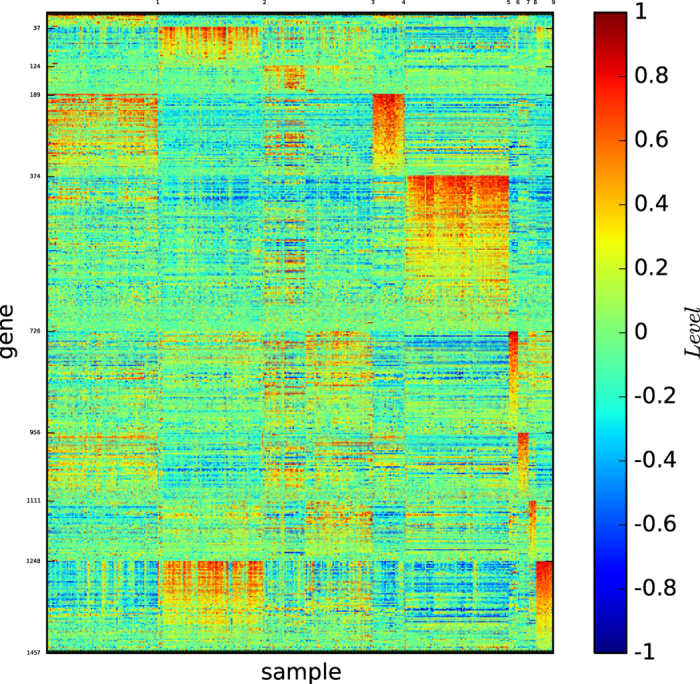
Gene map of the modules of new test leukemia data identified by 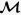. The 9 modules and their modules are provided in the supplementary information.

Figure 17 depicts the gene map of the modules of the new data Leukemia identified by the modularity maximisation algorithm 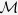.

Figure 17 reveals the following results: (1) The algorithm 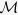 found 9 modules. (2) Modules 4, 5, 6, 7 and 8 are well-defined by the corresponding gene patterns, and all the other modules are not well-defined by the corresponding gene patterns.

### Gene map by InforMap

**Figure 18:**
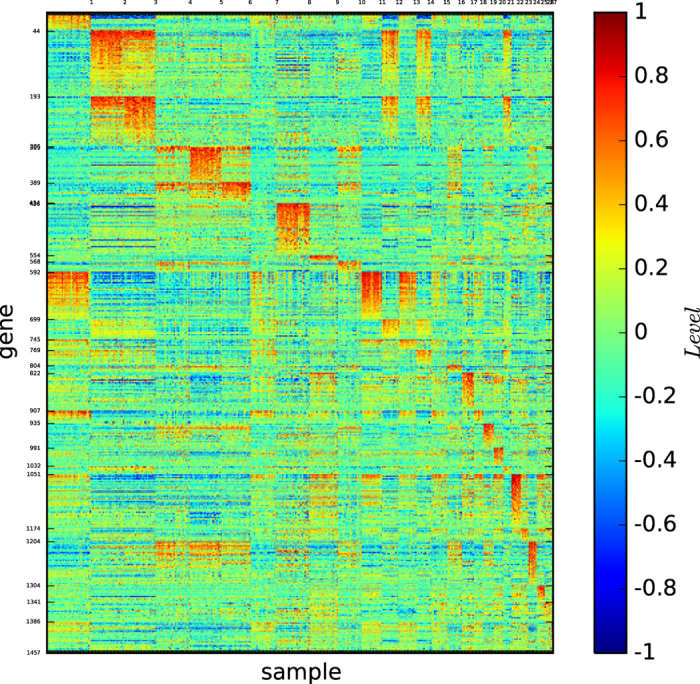
Gene map of the modules of new test leukemia data identified by 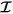. The 27 modules and their modules are provided in the supplementary information.

Figure 18 depicts the gene map of the new data Leukemia according to algorithm 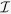.

Figure 18 reveals the following results: (1) The algorithm 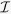 found 27 modules. (2) Most of the modules are not well-defined by the unique corresponding gene pattern.

### Gene map of the multi-tissues by structural entropy minimisation algorithm 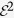


**Figure 19:**
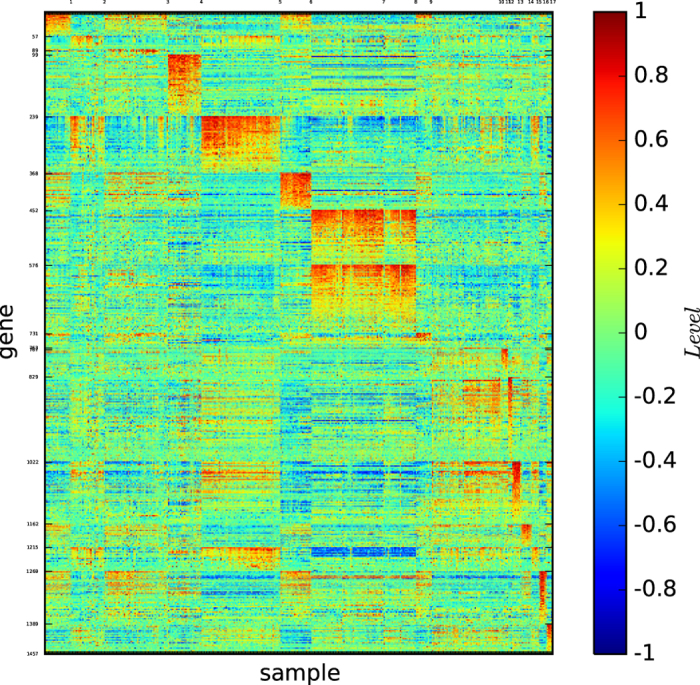
Gene map of the modules of new test leukemia data identified by 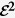. The 17 modules are exactly the same or almost the BR, CO, LU and PR respectively, for which the details are provided in the supplementary information.

Figure 19 depicts the gene map of the new data Leukemia provided by our algorithm 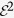.

Figure 19 reveals the following results: (1) The algorithm 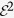 found 17 modules. (2) All the modules are distinguishable by the corresponding gene patterns. (3) Modules 3 and 10 are not well-defined by the unique corresponding gene patterns, and all the other modules are well-defined by their corresponding unique gene patterns.

### Three-dimensional gene map

**Figure 20:**
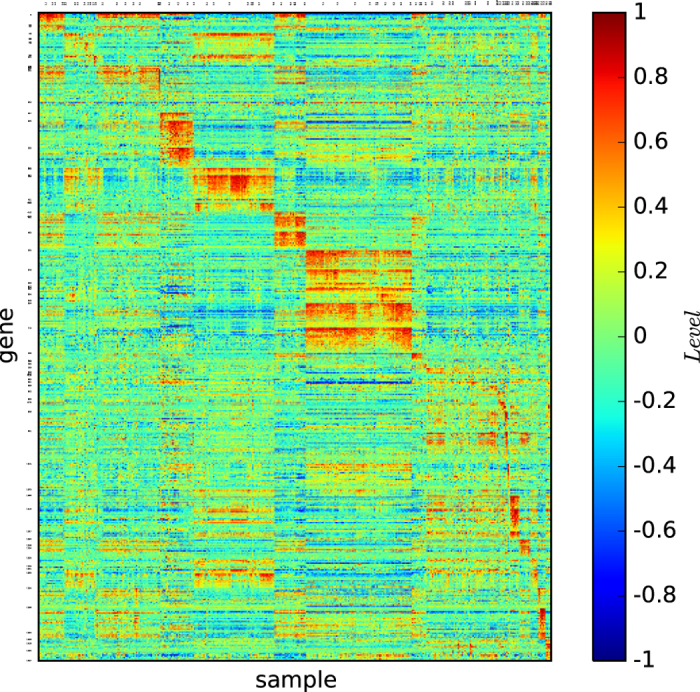
Gene map of the modules of new test leukemia data identified by 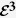. The 18 modules and their submodules are described in the supplementary information.

Figure 20 depicts the gene map of the new data Leukemia provided by our algorithm 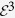.

Figure 20 reveals the following results: (1) The algorithm 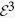 found 18 modules, each of which consists of a few submodules. (2) The 18 modules are distinguishable by the corresponding gene patterns, and most of the modules are well-defined by the corresponding unique gene patterns. (3) The submodules of a module are distinguishable by the corresponding gene patterns.

## Hypotheses and Criteria of Tumour Classification

Li, Li and Pan^17^ and Li *et al*.^18^ have shown that the minimisation of two-dimensional structural entropy is the principle for discovering natural communities in networks. This result indicates that the minimisation of two-dimensional structural entropy or equivalently, the minimisation of non-determinism of structures, is the principle of network self-organisation.

Our results reveal that the same principle holds true for defining tumour types and subtypes. We thus propose the following hypothesis.

*Hypothesis for defining the type and subtype of tumours*

- The construction of a network on the basis of gene expression profiles provides a global approach to defining the types and subtypes of tumours by network algorithms.
- One-dimensional structural entropy minimisation is the principle for constructing the network of unstructured data like the gene expression profiles.
- The partitioning of cell sample graphs provides an approach to defining tumour types and subtypes.
- Two-dimensional structural entropy minimisation is a principle of tumour-type classification.
- Three-dimensional structural entropy minimisation is a principle for defining tumour subtypes.

According to the definition, structural entropy minimisation minimises the non-determinism of structures within networks. This is a new principle for the self-organisation of many networks in nature and society. Here, we verify that the same principle holds for tumour classification. Furthermore, we conclude that high-dimensional structural entropy minimisation is the principle for structuring and processing big data in general. However, further studies will be required to provide a resolution for this hypothesis.

Unlike the high-dimensional structural entropy, the definition of the one-dimensional structural entropy does not imply any principle, because it is determined purely by the distributions of the edges and the corresponding weights. However, our results demonstrate that one-dimensional structural entropy minimisation could be a principle for us to detect the natural or true network that evolves in nature and society. This discovery is interesting, because, it means that although there are many reasons to affect the evolution of a real world network, the natural such network still follows some principles, for example, the principle of minimisation of non-determinism or uncertainty as explored by this research. Furthermore, our results demonstrate that the real world network may not simply be one of the networks generated purely by a random ensemble, it is in fact the network that follows the random ensemble towards minimisation of the one-dimensional structural entropy. This discovery may have deep implications in a wide range of disciplines. For example, it implies that there is a general principle that controls the formation and evolution of the natural structure of a real world complex system from random variations. This understanding is an analogy of Darwin’s evolution theory: natural selection is the principle that controls the evolution of species from random variations^16^. More importantly, our results demonstrate that one-dimensional structural entropy minimisation is the principle that controls the evolution of the natural structure from random variations. Therefore, our one-dimensional structural entropy could provide a quantitative measure to understand the laws of the nature. (For this reason, we suggest a future project to investigate the relationship between the structural entropy minimisation principle and Darwin’s natural selection).

Nevertheless, our results indicate that structural entropy minimisation, including the one-, two- and three-dimensional cases, is the principle for a new theory of big data in future computer science.

Considering the evaluation of an identified cancer type or subtype, a single criterion cannot verify the accuracy of a defined type or subtype of a cancer because cancer is a disease that has not been fully elucidated. Our results suggest that the true type or subtype of a cancer must simultaneously satisfy the criteria listed below.

### Criteria for verifying a type or subtype of a tumour

A defined type or subtype *T* is true, if it satisfies the following criteria:

1. (Similarity) *T* is similar to a type *T*′ defined in cancer biology.
2. (High-definition gene mapping) There is a set *B* of genes such that *B* highly expresses *T*, but fails to express any other cell types.
3. (Interpretability) Most of the cell samples in *T* share similar survival times, survival indicators and IPI scores.
4. (Prognostic distinction) Type *T* exhibits distinct overall survival times, survival ratios and IPI scores with other types in medical practices.

Theoretically, if an identified type or subtype *T* is highly expressed by a unique set of genes, then *T* should have a biological meaning; however, if it does not have a biological meaning, we must explain why the identified type or subtype *T* is biologically trivial when it is highly expressed by a unique and significantly large set of genes. According to this understanding, the criteria (1–3) above could be useful in identifying tumour types and subtypes. Criterion (4) requires that theoretical types or subtypes must be verified by medical practices.

Our results presented here demonstrate that the modules identified by the 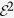 algorithm and the submodules identified by the 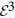 algorithm for different cancer types simultaneously satisfy the four criteria (1–4).

## Discussions

### Construction of a cell sample graph

Our theory depends on the construction of a cell sample graph determined by gene expression profiles. Our method is realised by appropriately choosing the parameter *k*. The choice of *k* must ensure that trivial or noisy profile weights are removed and that nontrivial profile weights are maintained. Therefore, *k* must not be too large or too small. More importantly, the choice of *k* depends on the data of the gene expression profiles. The principle we proposed here is to choose the *k* such that the one-dimensional structural entropy of the generated graph is the least among all stable points, that is, the points at which minimal one-dimensional structural entropy is achieved.

Our algorithm 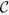 for choosing *k* is an approximated realisation of the general principle that one-dimensional structural entropy minimisation is a correct principle for networking of unstructured data. It works very well for the cell sample graph construction in the present paper.

However, the algorithm has some disadvantage, for example, we require that each cell sample keeps the edges of the top *k* weights. In real world, different cell sample may have to keep different numbers of edges. For this reason, we believe that there must be better methods to realise the general principle. The general principle allows us to construct interactive graphs for various kinds of data and sparsify networks. More optimal cell sample graphs may be constructed from the gene expression profiles, and these graphs may allow our algorithms 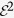 and 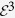 provide improved cancer classification. We believe this is still a grand challenge for future computer science.

### Challenges

The two- and three-dimensional gene maps developed by the algorithms 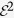 and 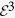 identify biologically and medically meaningful subtypes of tumours such that each subtype is defined by a unique gene expression pattern. The results provide new insights for cancer study.

Our three-dimensional gene map also demonstrates that, for a biologically and medically meaningful tumour subtype *X*, there is usually a large gene set *B* that defines *X*. In this case, the gene expression profiles fail to differentiate the genes in *B*. For cancer, however, it is important to select a small number of genes from *B* such that the small set of genes determines the subtype *X*.

Therefore, the three-dimensional gene map also suggests a fundamental challenge: for a subtype *X*, a small number of genes that essentially determines the subtype *X* should be identified.

Our three-dimensional gene map indicates that the gene expression profiles do not help to resolve this challenge.

## Conclusions

In this study, we propose a method of identifying the high-dimensional structural entropy of graphs for the construction of networks from gene expression profiles and we also propose the construction of heuristic algorithms 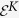 to detect the natural *K*-dimensional structure of networks by minimising the *K*-dimensional structural entropy, or the non-determinism of the *K*-dimensional structure of the networks. Algorithm 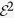 identifies the modules of the cell samples for five cancers and healthy tissue, and algorithm 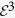 identifies the submodules of the cell samples of five cancers and healthy tissue. Almost all of the modules and submodules identified by our algorithms 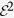 and 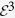 are defined by a unique gene expression pattern. By using currently available clinical data for type DLBCL lymphoma, we demonstrate that most samples in the DLBCL module or the submodule identified by 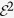 and 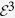 share similar survival times, survival indicators and IPI scores and indicate that distinct modules and submodules identified by 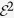 and 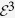 are distinguishable in overall survival times, survival ratios and IPI scores. Our results demonstrate that a tumour type may consist of several subtypes that satisfy the following criteria: (i) the subtypes are definable by a unique gene expression pattern; (ii) most of the samples of the same subtype share similar survival times, survival indicators and IPI scores; and (iii) different subtypes have distinct overall survival times, survival ratios and IPI scores. Our results demonstrate that the tumour subtypes satisfying the above criteria can be identified by our algorithms 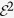 and 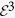. The algorithms 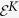 are used to minimise the *K*-dimensional structural entropy of networks. Therefore, high-dimensional structural entropy minimisation is the principle to define tumour types and subtypes. Our algorithms perform deep searches in networks, indicating that networking is the correct approach to defining tumour subtypes and their corresponding gene expression patterns. Our three-dimensional gene map of cancers provides the first high-definition, one-to-one map between biologically and medically meaningful subtypes and gene expression patterns, and our theory may have potential implications in cancer biology.

Our results demonstrate that one-dimensional structural entropy minimisation is the principle for networking of unstructured data, and that *K*-dimensional structural entropy minimisation is the principle for detecting the natural *K*-dimensional structures of real world networks for *K* > 1. The principle may have implications in a wide range of disciplines such as physics, biology, computer science, networking, and big data processing.

## Methods

### Normalisation of gene expression profiles

For a gene *g*, suppose that (*a*_1_, *a*_2_, ···, *a*_*n*_) is the vector of the gene expression profiles of *g* for all the samples *v*_1_, *v*_2_, ···, *v*_*n*_. We normalise the gene expression vector as follows:

1. Set 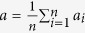 and 
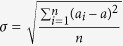
.
2. For each *i*, set 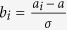.
3. Set 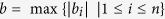.
4. For each *i*, set 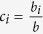.

According to the definition above, (*c*_1_, *c*_2_, ···, *c*_*n*_) is a coded vector of gene expression vector (*a*_1_, *a*_2_, ···, *a*_*n*_) such that the average value of *c*_*i*_ is 0, and each *c*_*i*_ is in the interval [−1, 1].

### Data analysis

The module and submodule of classifications for acute leukaemia, lymphoma, and multi-tissues are summarised in the supplementary information. We extract the top 10 genes for each of the modules or submodules identified by our algorithms 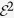 and 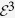 (supplementary information). We also analyse the gene map for three additional networks of lung cancer and healthy tissue, and the results are provided in the supplementary information.

## Additional Information

**How to cite this article**: Li, A. *et al*. Three-Dimensional Gene Map of Cancer Cell Types: Structural Entropy Minimisation Principle for Defining Tumour Subtypes. *Sci. Rep*. **6**, 20412; doi: 10.1038/srep20412 (2016).

## Supplementary Material

Supplementary Information

## Figures and Tables

**Table 1:**
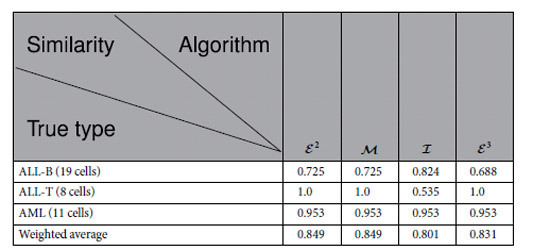
Similarity of the cell types of leukaemia identified by 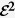, 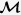, 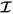 and 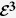.

**Table 2:**
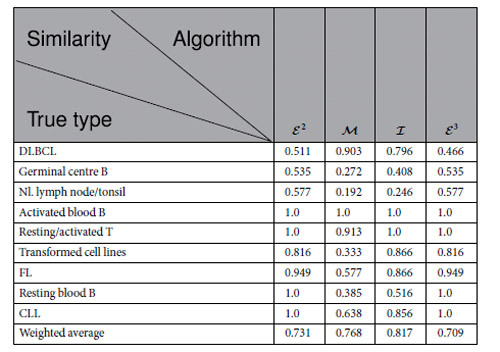
Similarity of the modules of the lymphoma found by 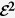, 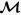, 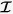 and 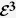.

**Table 3:**
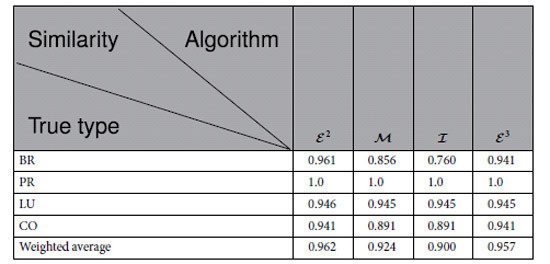
Similarity of the modules of multi tissue found by 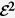, 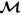, 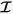 and 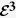.

**Table 4:**
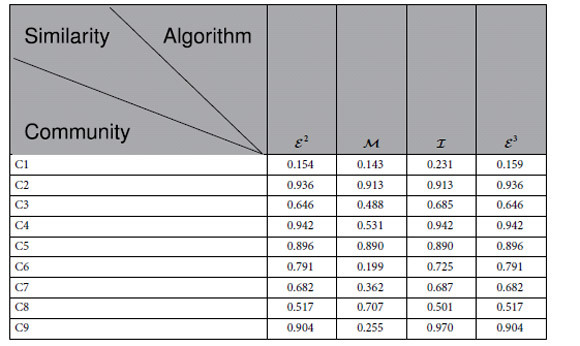
Similarity of new data Leukemia found by 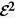, 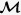, 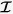 and 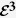-1.

**Table 5:**
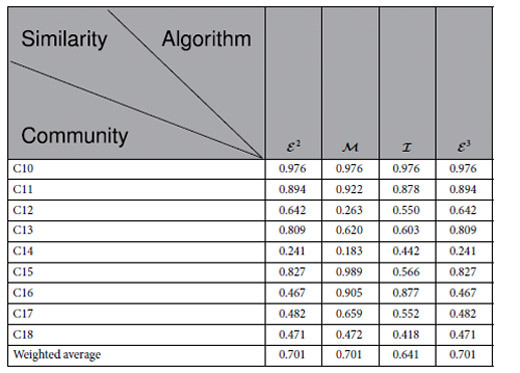
Similarity of new data Leukemia found by 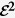, 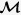, 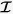 and 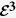-2.
