# Supplementary material for: Three-Dimensional Gene Map of Cancer Cell Types: Structural Entropy Minimisation Principle for Defining Tumour Subtypes
Source: Sci Rep. 2016 Feb 4;6:20412. doi: 10.1038/srep20412 (PMC4984903; doi:10.1038/srep20412)
Supplement: Supplementary Information [file srep20412-s1.pdf]

# Three-Dimensional Gene Map of Cancer Cell Types: Structural Entropy Minimisation Principle for Defining Tumour Subtypes (Supplementary Information) *

Angsheng Li, Xianchen Yin, Yicheng Pan
State Key Laboratory of Computer Science
Institute of Software, Chinese Academy of Sciences
Beijing, 100190, P. R. China

**Abstract**

We depict the curves of the one-dimensional structural entropy function of the algorithm $\mathcal{C}$ for the acute leukaemia, lymphoma, multi-tissues, lung cancer and healthy tissue. We describe the modules of acute leukaemia, lymphoma and multi-tissues identified by the algorithms $\mathcal{M}$, $\mathcal{I}$ and our new algorithms $\mathcal{E}^2$ and $\mathcal{E}^3$, and describe the modules and gene maps of lung cancer, and healthy tissue defined by the algorithms $\mathcal{M}$, $\mathcal{I}$ and our new algorithms $\mathcal{E}^2$ and $\mathcal{E}^3$. We report the statistic survival times, survival indicators and IPI scores of the subtypes of the DLBCL lymphoma identified by the algorithms $\mathcal{M}$, $\mathcal{I}$ and our new algorithms $\mathcal{E}^2$ and $\mathcal{E}^3$.

## Gene Expression Data

In this section, we introduce the gene expression data for five cancers and healthy tissue as follows:

1. Acute leukaemia

   The data are from [1], which are obtained from acute leukaemia patients at the time of diagnosis. The data contain the expression of $7,129$ genes for $38$ samples which form $3$ cell types. The three tumor types obtained from acute leukaemia patients at the time of diagnosis are: $11$ acute myeloid leukaemia (AML) samples; $8$ T-lineage acute lymphoblastic leukaemia (ALL) samples; and $19$ B-lineage ALL samples.

*The authors are partially supported by the Grand Project "Network Algorithms and Digital Information" of the Institute of Software, Chinese Academy of Sciences, by an NSFC grant No. 61161130530, by a High-Tech Program (863) Grant No. 2012AA8113011, and by a 973 program Grant No. 2014CB340302.

2. Lymphoma

   The data are from [2]. The data contain the expression of $4,026$ genes for $96$ samples which form $9$ cell types. The $9$ types consist of three different types of tumors, i.e., diffuse large B cell lymphoma (DLBCL), chronic lymphocytic leukaemia (CLL), and follicular lymphoma (FL), as well as normal B and T cells at different stages of cell differentiation, including germinal centre B, NL. lymph node/tonsil, activated blood B, resting/activated T, transformed cell lines, and resting blood B etc.

   [2] suggested to divide the DLBCL type into two subtypes, the GC B-like DLBCL and the activated B-like DLBCL using gene expression profiling. The GC B-like and activated B-like DLBCLs were associated with statistically significant differences in over all survival (P $<$ 0.01) and in event-free survival.

3. Multi-tissues

   The dataset of multi-tissues are from [3]. The data contain the expression of $5,565$ genes for $103$ samples which form $4$ cell types. The tissue samples are from four distinct cancer types: $26$ breast, $26$ prostate, $28$ lung, and $23$ colon samples.

4. Lung cancer

   The dataset of lung cancer are from [9]. It uses a simple signal-to-noise ratio (SNR) to rank genes. The final gene pool is obtained by selecting the most upregulated genes for each class, where the exact number depends on the original dataset [5]. The data in [9] contain the expression of $1,000$ genes for 197 samples which form $4$ cell types. The four known classes are: $139$ adenocarcinomas (AD), $21$ squamous cell carcinomas (SQ), $20$ carcinoids (COID), and $17$ normal lung (NL), where the AD class is highly heterogeneous, and substructure is known to exist, although not well understood.

5. Healthy tissue

   We use the dataset healthy tissue in [6]. It uses a simple signal-to-noise ratio (SNR) to rank genes. The final gene pool is obtained by selecting the most upregulated genes for each class, where the exact number depends on the original dataset in [3]. The data in [6] contain the expression of $1,277$ genes for $90$ samples which form 13 cell types. The $13$ distinct tissue types are: breast (5), prostate (9), lung (7),colon (11), germinal center cells (6), bladder (7), uterus (6), peripheral blood monocytes (5), kidney (12), pancreas (10), ovary (4), whole brain (5), and cerebellum (3).

6. The large test data for leukemia

   We use the test data for leukemia from [8].

# One-dimensional Structure Entropy

In this section, we depict the curves of the one-dimensional structure entropy function of the gene expression profiles of the acute leukaemia, the lymphoma, the multi-tissues, the lung cancer and the healthy tissue. In the implementation of the algorithm $\mathcal{C}$, we fix a noise amplifier $\sigma = \frac{1}{2n}$, where $n$ is the number of cell samples. We choose the parameter $k$ from 1 to 20. Here, the upper bound 20 has no specific meaning. It only means that $k$ must not be too large.

Figure 1 depicts the curve of the one-dimensional structure entropy function of the gene expression of the acute leukaemia.

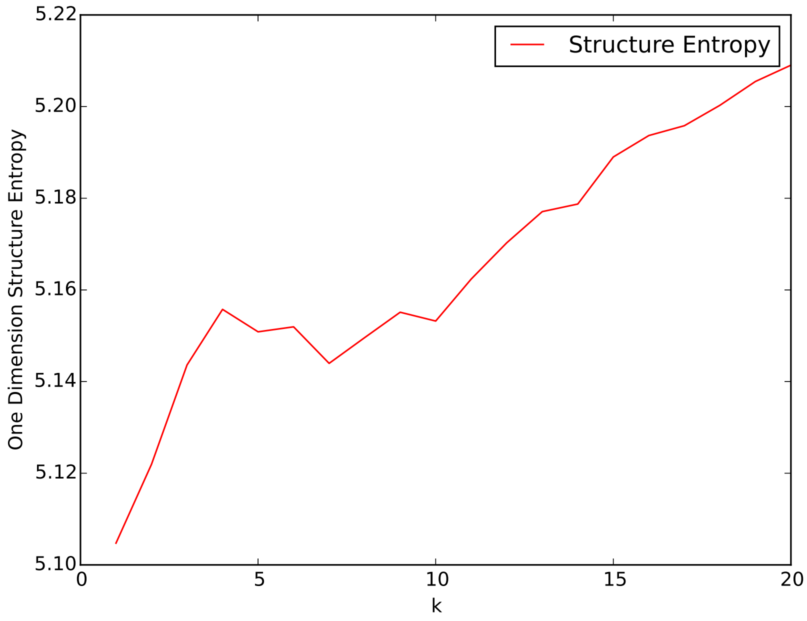


Figure 1: One-dimensional structure entropy of gene graph for the acute leukaemia. The figure shows that the output of algorithm $\mathcal{C}$ for the acute leukaemia is $k = 7$.

According to Figure 1, we have that the output of algorithm $\mathcal{C}$ for the acute leukaemia is $k = 7$.

Figure 2 depicts the curve of the one-dimensional structure entropy function of the gene expression of the lymphoma.

According to Figure 2, we have that the output of algorithm $\mathcal{C}$ for the lymphoma is $k = 6$.

Figure 3 depicts the curve of the one-dimensional structure entropy function of the gene expression of the multi-tissues.

According to Figure 3, we have that the output of algorithm $\mathcal{C}$ for the multi-tissues

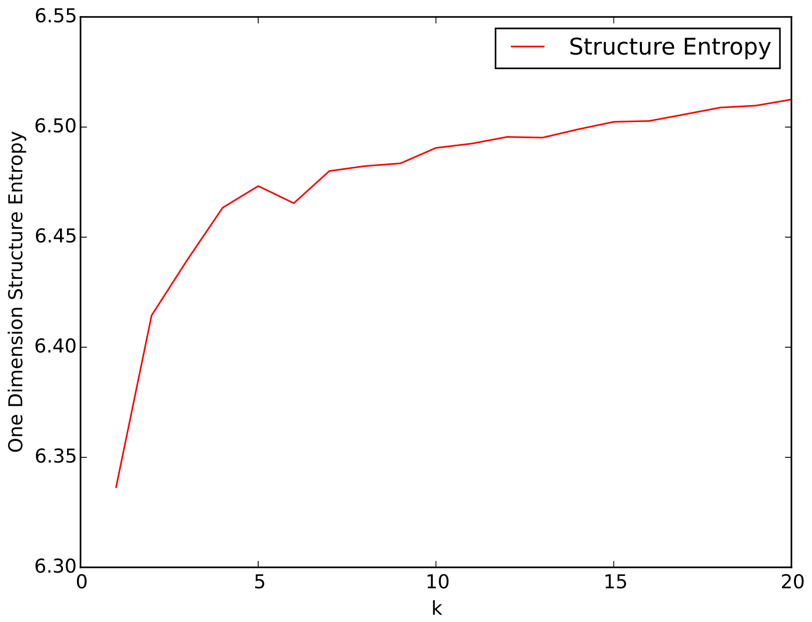


Figure 2: One-dimensional structure entropy of gene graph for the lymphoma. The figure shows that the output of algorithm $\mathcal{C}$ for the lymphoma is $k = 6$.

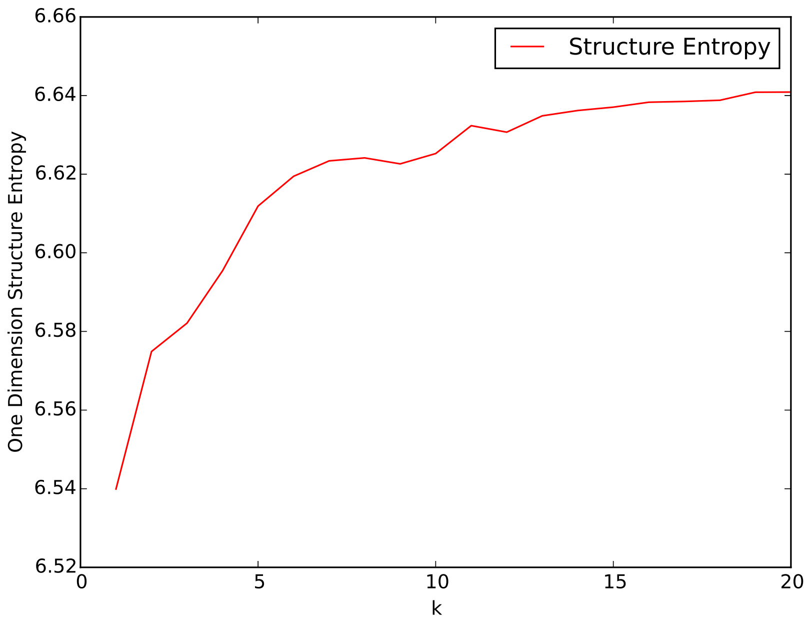


Figure 3: One-dimensional structure entropy of gene graph for the multi-tissues. The figure shows that the output of algorithm $\mathcal{C}$ for the multi-tissues is $k = 9$.

is $k = 9$.

Figure 4 depicts the curve of the one-dimensional structure entropy of the cell sample networks of the test data for leukemia in [8].

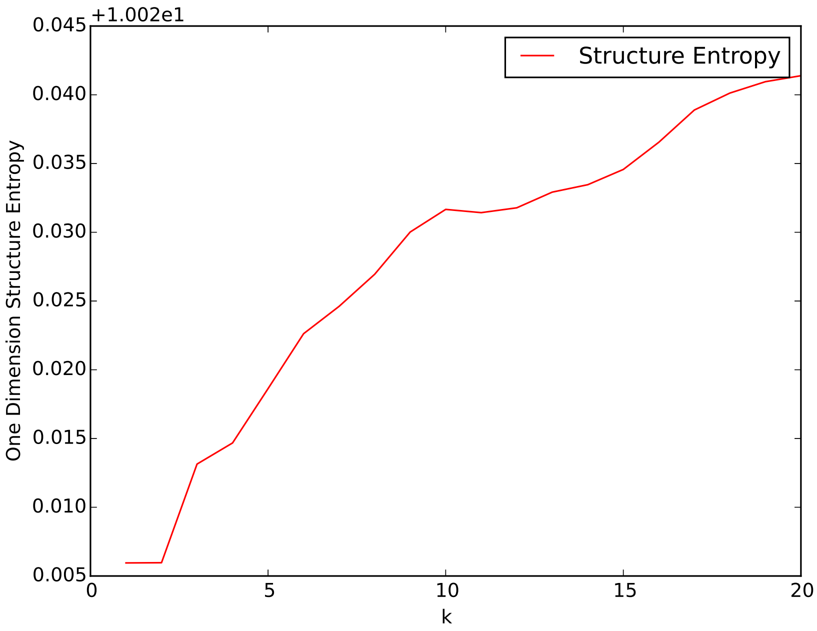


Figure 4: One-dimensional structure entropy of gene graph for the test data of leukemia. The figure shows that the output of algorithm $\mathcal{C}$ for the normal tissue is $k = 11$.

Figure 5 depicts the curve of the one-dimensional structure entropy function of the gene expression of the lung cancer.

According to Figure 5, we have that the output of algorithm $\mathcal{C}$ for the lung cancer is $k = 6$.

Figure 6 depicts the curve of the one-dimensional structure entropy function of the gene expression of the normal tissue.

According to Figure 6, we have that the output of algorithm $\mathcal{C}$ for the normal tissue is $k = 3$.

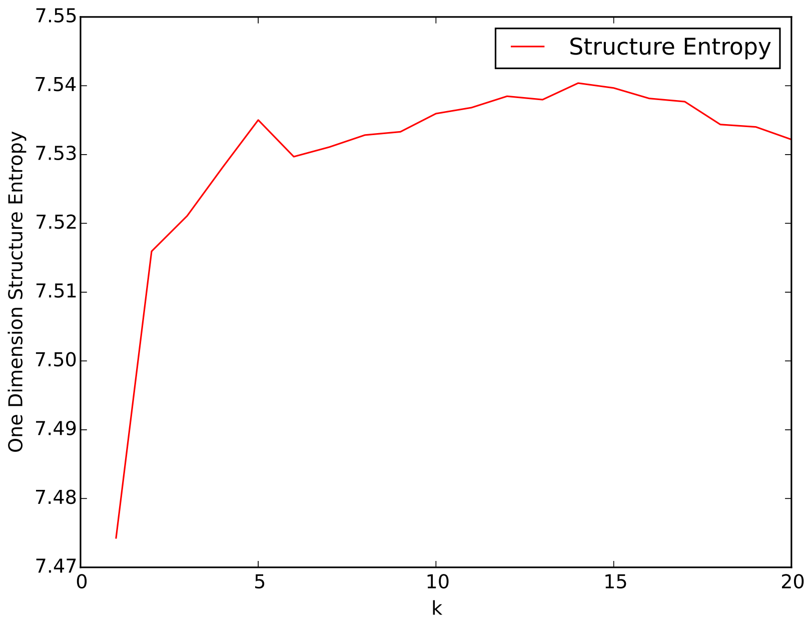


Figure 5: One-dimensional structure entropy of gene graph for the lung cancer. The figure shows that the output of algorithm $\mathcal{C}$ for the lung cancer is $k = 6$.

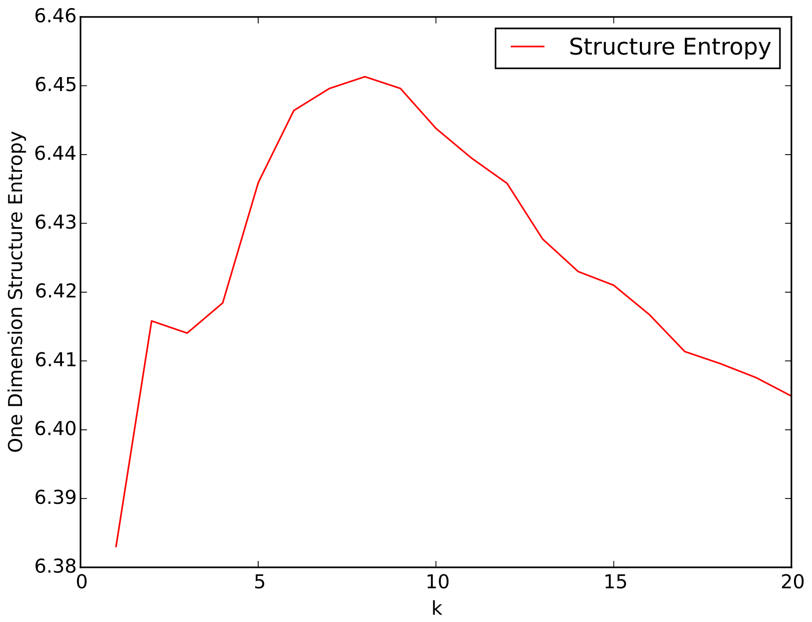


Figure 6: One-dimensional structure entropy of gene graph for the normal tissue. The figure shows that the output of algorithm $\mathcal{C}$ for the normal tissue is $k = 3$.

# Acute Leukaemia

## True types

Table 1 describes the true types of the acute leukaemia.

| True type | Samples | Cell type |
|---|---|---|
| 1 | ALL_19769_B-cell, ALL_23953_B-cell, ALL_28373_B-cell, ALL_9335_B-cell, ALL_9692_B-cell, ALL_14749_B-cell, ALL_17281_B-cell, ALL_19183_B-cell, ALL_20414_B-cell, ALL_21302_B-cell, ALL_549_B-cell, ALL_17929_B-cell, ALL_20185_B-cell, ALL_11103_B-cell, ALL_18239_B-cell, ALL_5982_B-cell, ALL_7092_B-cell, ALL_R11_B-cell, ALL_R23_B-cell | ALL-B |
| 2 | ALL_16415_T-cell, ALL_19881_T-cell, ALL_9186_T-cell, ALL_9723_T-cell, ALL_17269_T-cell, ALL_14402_T-cell, ALL_17638_T-cell, ALL_22474_T-cell | ALL-T |
| 3 | AML_12, AML_13, AML_14, AML_16, AML_20, AML_1, AML_2, AML_3, AML_5, AML_6, AML_7 | AML |

Table 1: True types of the cell samples of acute leukaemia

Figure 1 reveals the following results:

1) The types (ALL-B, ALL-T, and AML) are distinguishable because they are well-defined by different sets of genes, i.e., genes 1 to 2,326, 2,327 to 4,608, and 4,609 to 7, 129 marked in Figure 1 and denoted by blocks $B_1$, $B_2$ and $B_3$, respectively.

2) The genes in $B_1$ have high expression profiles for ALL-B but low expression profiles for ALL-T and AML.

3) The genes in $B_2$ have the highest expression profiles for ALL-T, low profiles for AML, and significantly high profiles for ALL-B.

4) The genes in $B_3$ have high profiles for AML but low profiles for ALL-B and ALL-T.

5) There are cells in ALL-B that are highly expressed by many genes in all three blocks $B_1$, $B_2$ and $B_3$.

6) The expression level of tumour type ALL-B by $B_1$ is less than that of ALL-T by $B_2$ and of AML by $B_3$.

## Modules identified by $\mathcal{M}$

Table 2 describes the modules of acute leukaemia identified by the algorithm $\mathcal{M}$.

Figure 2 and S-Table 2 (Table 2 in the supplementary information, denoted S-Table 2) reveal the following results:

| Modules | Samples | Cell type |
|---|---|---|
| 1 | ALL_19769_B-cell, ALL_23953_B-cell, ALL_9335_B-cell, AML_13, ALL_18239_B-cell, ALL_R23_B-cell, ALL_9692_B-cell, ALL_14749_B-cell, ALL_17929_B-cell | ALL-B, AML |
| 2 | ALL_28373_B-cell, ALL_17281_B-cell, ALL_20414_B-cell, ALL_21302_B-cell, ALL_20185_B-cell, ALL_5982_B-cell, ALL_19183_B-cell, ALL_549_B-cell, ALL_11103_B-cell, ALL_R11_B-cell | ALL-B |
| 3 | ALL_7092_B-cell | ALL-B |
| 4 | ALL_16415_T-cell, ALL_19881_T-cell, ALL_9186_T-cell, ALL_22474_T-cell, ALL_17269_T-cell, ALL_14402_T-cell, ALL_9723_T-cell, ALL_17638_T-cell | ALL-T |
| 5 | AML_12, AML_14, AML_3, AML_16, AML_7, AML_6, AML_1, AML_5, AML_20, AML_2 | AML |

Table 2: The modules of the cell samples of acute leukaemia identified by $\mathcal{M}$

(1) Module 1 consists of certain ALL-B samples with AML_13.

(2) Module 2 is a subset of ALL-B, and module 3 consists of only ALL_7092_B_cells.

(3) Module 4 is the correct ALL-T.

(4) Module 5 is the AML missing AML_13.

(5) Each of the modules is defined by a set of genes.

## Modules identified by $\mathcal{I}$

Table 3 describes the modules of acute leukaemia identified by $\mathcal{I}$.

| Modules | Samples | Cell type |
|---|---|---|
| 1 | ALL_19769_B-cell, ALL_23953_B-cell, ALL_28373_B-cell, ALL_9335_B-cell, ALL_9692_B-cell, ALL_14749_B-cell, ALL_17281_B-cell, ALL_19183_B-cell, ALL_20414_B-cell, ALL_21302_B-cell, ALL_549_B-cell, ALL_17929_B-cell, ALL_20185_B-cell, ALL_11103_B-cell, ALL_18239_B-cell, ALL_5982_B-cell, ALL_7092_B-cell, ALL_R11_B-cell, ALL_R23_B-cell, ALL_16415_T-cell, ALL_19881_T-cell, ALL_9186_T-cell, ALL_9723_T-cell, ALL_17269_T-cell, ALL_14402_T-cell, ALL_17638_T-cell, ALL_22474_T-cell, AML_13 | ALL-B, ALL-T, AML |
| 2 | AML_12, AML_14, AML_16, AML_20, AML_1, AML_2, AML_3, AML_5, AML_6, AML_7 | AML |

Table 3: The modules of the cell samples of acute leukaemia identified by $\mathcal{I}$

## Modules identified by $\mathcal{E}^2$

Table 4 describes the modules of acute leukaemia identified by $\mathcal{E}^2$.

| Module | Samples | Cell type |
|---|---|---|
| 1 | ALL_19769_B-cell, ALL_23953_B-cell, ALL_9335_B-cell, AML_13, ALL_18239_B-cell, ALL_R23_B-cell, ALL_9692_B-cell, ALL_14749_B-cell, ALL_17929_B-cell, ALL_7092_B-cell | ALL-B (but AML_13) |
| 2 | ALL_28373_B-cell, ALL_17281_B-cell, ALL_20185_B-cell, ALL_19183_B-cell, ALL_20414_B-cell, ALL_5982_B-cell, ALL_549_B-cell, ALL_R11_B-cell, ALL_11103_B-cell, ALL_21302_B-cell | ALL-B |
| 3 | ALL_16415_T-cell, ALL_19881_T-cell, ALL_9186_T-cell, ALL_22474_T-cell, ALL_17269_T-cell, ALL_14402_T-cell, ALL_9723_T-cell, ALL_17638_T-cell | ALL-T |
| 4 | AML_12, AML_14, AML_16, AML_7, AML_3, AML_1, AML_6, AML_5, AML_20, AML_2 | AML (missing AML_13) |

Table 4: The modules of cell samples of acute leukaemia identified by $\mathcal{E}^2$.

Figure 4 and S-Table 4 show the following results:

(1) Module $1$ consists of ALL_19769_B-cell, ALL_23953_B-cell, ALL_9335_B-cell, AML_13, ALL_18239_B-cell, ALL_R23_B-cell, ALL_9692_B-cell, ALL_14749_B-cell, ALL_17929_B-cell, and ALL_7092_B-cell, which is a subtype of ALL-B (except for AML_13), and it is defined by a set of more than $800$ genes.

(2) Module $2$ consists of ALL_28373_B-cell, ALL_17281_B-cell, ALL_20185_B-cell, ALL_19183_B-cell, ALL_20414_B-cell, ALL_5982_B-cell, ALL_549_B-cell, ALL_R11_B-cell, ALL_11103_B-cell, and ALL_21302_B-cell, which is a subtype of ALL-B, and it is defined by a set of more than $2,500$ genes.

(3) Module $3$ is exactly the type ALL-T, and is defined by a set of more than $1,000$ genes.

(4) Module $4$ is the type AML (missing AML_13), and is defined by a set of more than $1,000$ genes.

Table 4 shows that:

(i) Our algorithm $\mathcal{E}^2$ divides the type ALL-B into two modules, Modules 1 and 2.

(ii) Our algorithm $\mathcal{E}^2$ misclassifies AML_13 to Module 1, a subset of ALL-B.

(iii) Except for (i) and (ii) above, our algorithm $\mathcal{E}^2$ is consistent with the classifications of the true types.

(i) implies that ALL-B may really consist of two subtypes. (ii) is interesting in the sense that it poses a question: Why does our algorithm $\mathcal{E}^2$ fail to assign this cell correctly? (iii) means that our algorithm is almost correct. More importantly, (i) - (iii) mean that the "errors" of our algorithm $\mathcal{E}^2$ may imply new interesting discoveries.

In Table 5, we describe the top 10 genes of the modules identified by $\mathcal{E}^2$ as shown in Table 4 and the true types listed in Table 1.

| Modules identified by $\mathcal{E}^2$ | Top 10 genes of modules identified by $\mathcal{E}^2$ | Top 10 genes of true types | Type |
|---|---|---|---|
| 1 | U43885_at, D12763_at, U66580_at, U64871_at, L10338_s_at, U36798_at, AFFX-M27830_5_at, J00268_s_at, M96944_at, U00951_at | M28170_at, U05259_rna1_at, X00274_at, L06797_s_at, X63469_at, X58529_at, M84371_rna1_s_at, U43885_at, M73547_at, M29551_at | ALL-B |
| 2 | X77366_at, X63469_at, D26018_at, M29551_at, U03911_at, U62293_rna1_s_at, HG2887-HT3031_at, L39060_at, D79990_at, U90552_at | M28170_at, U05259_rna1_at, X00274_at, L06797_s_at, X63469_at, X58529_at, M84371_rna1_s_at, U43885_at, M73547_at, M29551_at | ALL-B |
| 3 | X04391_at, M37815_cds1_at, X03934_at, L05148_at, M16279_at, X73358_s_at, X69398_at, U23852_s_at, U04241_at, M26692_s_at | X04391_at, M37815_cds1_at, X03934_at, L05148_at, M16279_at, X73358_s_at, X69398_at, U23852_s_at, U04241_at, M26692_s_at | ALL-T |
| 4 | U82759_at, Z48501_s_at, X17042_at, M21904_at, M28209_at, M62762_at, M11147_at, Y00433_at, D49950_at, U81554_at | U82759_at, D49950_at, X17042_at, Z48501_s_at, M55150_at, M11147_at, M62762_at, M21904_at, M21551_rna1_at, Y00433_at | AML |

Table 5: Top 10 genes of the modules of acute leukaemia identified by $\mathcal{E}^2$ and the true types

Table 5 shows the following properties:

i) The top 10 genes of Module 1 is disjoint with that of the ALL-B.

ii) The join of the top 10 genes of Module 2 and ALL-B contains only X63469_at and M29551_at.

iii) The join of the top 10 genes of Module 4 and AML contains 8 genes, where

| Modules | Submodules | Cell type |
|---|---|---|
| 1 | ALL_19769_B-cell, ALL_9692_B-cell, ALL_17929_B-cell | |
| | ALL_23953_B-cell, ALL_14749_B-cell | |
| | ALL_9335_B-cell, AML_13, ALL_18239_B-cell, ALL_R23_B-cell | |
| 2 | ALL_28373_B-cell, ALL_549_B-cell, ALL_5982_B-cell | |
| | ALL_17281_B-cell, ALL_20185_B-cell, ALL_19183_B-cell | |
| | ALL_20414_B-cell, ALL_R11_B-cell, ALL_11103_B-cell | |
| 3 | ALL_21302_B-cell | |
| 4 | ALL_7092_B-cell | |
| 5 | ALL_16415_T-cell, ALL_14402_T-cell, ALL_9723_T-cell, ALL_17638_T-cell | |
| | ALL_19881_T-cell, ALL_9186_T-cell, ALL_22474_T-cell, ALL_17269_T-cell | |
| 6 | AML_12, AML_6, AML_5 | |
| | AML_14, AML_16, AML_7, AML_3 | |
| | AML_1, AML_20, AML_2 | |

Table 6: The modules and submodules of cell samples of acute leukaemia identified by $\mathcal{E}^3$

M28209_at and U81554_at are the extra two more genes for Module 4, M55150_at, and M21551_rna1_at are the extra two more genes for AML.

i) - ii) show that the top genes for a subset of a type are very much different from that of the type. iii) means that a minor difference of classification of cells may make a big difference of the top genes expressing the modules and the types. The results also indicate that dividing a type into submodules may make a big difference for understanding the expressing genes, the mechanisms of cells, the diagnosis and therapy of cancers.

## Submodules identified by $\mathcal{E}^3$

In Table 6, we describe the types and subtypes of cell samples of acute leukaemia identified by our algorithm $\mathcal{E}^3$.

Figure 5 and S-Table 6 reveal the following results:

(1) Algorithm $\mathcal{E}^3$ identified 6 modules. Module 1 is of the subtype of ALL-B and consists of 3 submodules, 1.1: ALL_19769_B-cell, ALL_9692_B-cell, ALL_17929_B-cell; 1.2: ALL_23953_B-cell, ALL_14749_B-cell; and 1.3: ALL_9335_B-cell, AML_13, ALL_18239_B-cell, ALL_R23_B-cell (except for AML_13). Module 2 is of the subtype of ALL-B and consists of 3 submodules, 2.1: ALL_28373_B-cell, ALL_549_B-cell, ALL_5982_B-cell; 2.2: ALL_17281_B-cell, ALL_20185_B-cell, ALL_19183_B-cell; and 2.3: ALL_20414_B-cell, ALL_R11_B-cell, ALL_11103_B-cell. Modules 3 and 4 contain a single cell, ALL_21302_B-cell and ALL_7092_B-cell, respectively. Module 5 is of the type ALL-T and consists

of two submodules, 5.1: ALL_16415_T-cell, ALL_14402_T-cell, ALL_9723_T-cell, ALL_17638_T-cell; and 5.2: ALL_19881_T-cell, ALL_9186_T-cell, ALL_22474_T-cell, ALL_17269_T-cell. Module 6 is of the type AML, missing AML_13 and consists of three subtypes, 6.1: AML_12, AML_6, AML_5; 6.2: AML_14, AML_16, AML_7, AML_3; and 6.3: AML_1, AML_20, AML_2.

We use $X_i$ to denote module $i$, and $Y_{i,j}$ to denote the $j$-th submodule of $X_i$. We use $B_{i,j}$ to denote the set of genes that determine the submodule $Y_{i,j}$, which is marked on the left side boundary of Figure 5.

(2) For each pair $i$, $j$, the submodule $Y_{i,j}$ is defined by the genes in $B_{i,j}$.

(3) Every gene is in a set $B_{i,j}$ for certain $i$, $j$.

(4) For every $i$, $X_i$ is a precise subtype of certain true cell type.

(5) For all $i$ and $j$, most genes in $B_{i,j}$ highly express submodule $Y_{i,j}$ and few genes in $B_{i,j}$ significantly express any module or submodule other than $Y_{i,j}$.

In this case, we say that $Y_{i,j}$ is defined by the set of genes $B_{i,j}$, or $B_{i,j}$ defines submodule $Y_{i,j}$.

(6) For each pair $i$ and $j$, submodule $Y_{i,j}$ is defined by set $B_{i,j}$.

(7) Submodule 2.2 consists of ALL_17281_B-cell, ALL_20185_B-cell, and ALL_19183_B-cell, and it is defined by a set of more than $1,000$ genes.

(8) Module 3 consists of a single cell, ALL_21302_B-cell and is defined by a set of more than $2,000$ genes.

(9) Module $4$ consists of a single cell, ALL_7092_B-cell and is defined by a set of more than $400$ genes.

(10) Submodule 6.2 consists of AML_14, AML_16, AML_7, and AML_3 and is defined by a set of more than $700$ genes.

Results (1) to (6) imply that each module $X_i$ is either a true type or a subset of a true type that consists of several distinguishable submodules and indicate that all of the modules are distinguishable and each submodule $Y_{i,j}$ is uniquely determined by a block of genes $B_{i,j}$, which generates a high-definition and one-to-one map from the submodules $Y_{i,j}$ to gene expression patterns $B_{i,j}$ for all $i$ and $j$. It is conceivable that an analysis of gene set $B$ that defines a module $X$ or a submodule $Y$ may be required to treat the corresponding module $X$ or submodule $Y$. However, our results show that a module $X$ or a submodule $Y$ usually has a large gene set $B$ that expresses the module $X$ or submodule $Y$, which could lead to difficulty in treating a tumour type. To address this issue, our three-dimensional gene map analysis suggests that for a tumour type $X$, we may divide $X$ into submodules $Y_1, Y_2$ and $Y_3$. Upon analysis, the gene sets $B_1$, $B_2$ and $B_3$ may express $Y_1$, $Y_2$ and $Y_3$, respectively. This analysis could aid in treating the tumour type $X$. Nevertheless, we believe that it is fundamental to identify and analyse the large set of genes that express a biologically meaningful module or submodule, and our three-dimensional cancer gene map can provide this ability.

The results (1) to (10) demonstrate that all of the modules and submodules classified by our algorithm $\mathcal{E}^3$ maybe biologically meaningful. In particular, according to (8) and (9), ALL_21302_B-cell and ALL_7092_B-cell are remarkably cells that may play essential roles in the classification, diagnosis and therapy of acute leukaemia. According to (7) and (10), submodules 2.2 and 6.2 could be extremely important in the classification, diagnosis and therapy of acute leukaemia.

Table 6 shows the following results:

(i) Modules 1, 2, 5 and 6 are almost the same as the modules 1, 2, 3 and 4 identified by $\mathcal{E}^2$ respectively.

(ii) Modules 3 and 4 are both singletons.

(iii) Each of Modules 1, 2, 5 and 6 is further divided into several submodules.

(iv) Our algorithm $\mathcal{E}^3$ fails to identify AML_13 again.

Our three-dimensional gene map shows that the classification in Table 6 has a high-definition, one-to-one map between the submodules and the gene expression patterns, although it fails to assign cell AML_13 correctly.

In Table 7, we describe the top 10 genes that express the submodules of acute leukaemia in Table 6.

| Submodules | Top 10 genes of submodules identified by $\mathcal{E}^3$ |
|---|---|
| 1.1 | U43885_at, X93017_at, Y10517_at, M25164_at, D29992_at, D86974_at, X00274_at, L38503_at, M27504_s_at, X16354_at |
| 1.2 | X87767_at, M85220_at, U13044_at, M64572_at, S72043_rna1_at, AFFX-BioB-M_st, X53795_at, K02215_at, M93311_at, X52896_s_at |
| 1.3 | X03350_at, L06499_at, X80822_at, U63332_at, HG1428-HT1428_s_at, U14973_at, D89501_at, U36798_at, X12794_at, M96944_at |
| 2.1 | M74542_at, U62293_rna1_s_at, D14660_at, M16653_at, X77366_at, X99961_at, U97188_at, U00957_at, L07033_at, X90846_s_at |
| 2.2 | X17093_at, M29551_at, X59417_at, Z35102_at, AB000410_s_at, HG3576-HT3779_f_at, M63838_s_at, M83738_at, L29376_at, L06175_at |
| 2.3 | M28170_at, X58987_at, D79990_at, HG2887-HT3031_at, X99657_at, U27699_at, HG544-HT544_at, D38305_at, D88270_at, L13698_at |
| 3 | AFFX-CreX-5_st, AFFX-LysX-5_at, AB000460_at, AB002366_at, AB003698_at, AF002020_at, AF008937_at, AF012270_at, AF014958_at, AF015910_at |
| 4 | AFFX-M27830_5_at, D85429_at, HG4724-HT5166_at, J03764_at, L02321_at, L09604_at, L76687_at, M11717_rna1_at, M12759_at, M22489_at |
| 5.1 | X73358_s_at, Z50853_at, U04241_at, L05148_at, X04391_at, U81001_at, D85131_s_at, M23323_s_at, U67122_s_at, M85217_at |
| 5.2 | V00599_s_at, M13792_at, U93049_at, U78180_at, M28826_at, X03934_at, M37815_cds1_at, M14483_rna1_s_at, U89916_at, M26708_s_at |
| 6.1 | D55640_at, HG2614-HT2710_at, M23197_at, U17327_at, U57093_at, U82759_at, X83573_at, D31846_at, Z29067_at, U60666_at |
| 6.2 | D16217_at, M57710_at, U06681_at, D79206_s_at, Z48501_s_at, M28209_at, M13485_at, X17042_at, D87989_at, D25547_at |
| 6.3 | M77810_at, X85237_at, Z32684_at, U63289_at, M33772_s_at, AFFX-TrpnX-3_at, U03399_at, M19309_s_at, Y10510_at, M63589_at |

Table 7: Top 10 genes of submodules identified by $\mathcal{E}^3$

# Lymphoma

## True types

Table 8 lists the true types of lymphoma.
Figure 6 reveals the following results:

1) All of the types are distinguishable because they are defined by different blocks of genes.

2) All of the types except DLBCL are expressed by different sets of genes.

3) The type DLBCL is a large set; however, it is not well-expressed.

4) Four types (germinal centre B, NL. lymph node/tonsil, resting/activated T and transformed cell lines) are highly expressed by a set of many genes; thus, the blocks of genes expressing the types are large.

5) Except for DLBCL, the $8$ remaining types are highly expressed by their corresponding blocks of genes.

## Modules identified by $\mathcal{M}$

Table 9 describes the modules of lymphoma identified by $\mathcal{M}$.
Figure 7 and S-Table 9 reveal the following properties:

(1) Algorithm $\mathcal{M}$ identified $4$ modules. The first module consists of DLBCL, NL. lymph node/tonsil, transformed cell lines, and resting/ activated T; the second module consists of germinal centre B, FL, DLBCL, resting blood B, and CLL; the third module consists of activated blood B; and the fourth module consists of resting/ activated T.

(2) The four modules identified by $\mathcal{M}$ are distinguishable by different sets of genes.

(1) indicates that the modules identified by $\mathcal{M}$ are far from the true types. (1) and (2) imply that gene expression patterns alone is insufficient for evaluating the modules identified by a community detection algorithm.

## Modules identified by $\mathcal{I}$

Table 10 describes the modules of the lymphoma identified by $\mathcal{I}$.
Figure 8 and S-Table 10 reveal the following results:

(1) Module 1 consists of DLBCL and NL. lymph node/tonsil; module 2 consists of resting blood B and CLL; module 3 consists of germinal centre B, FL, and DLBCL; module 4 consists of activated blood B; module 5 consists of resting/ activated T; module 6 consists of transformed cell lines and DLBCL; and modules 7, 8 and 9 consist of certain DLBCL cell samples.

(2) Each of the $9$ modules is defined by a unique set of genes.

(3) The large DLBCL type is divided, and the DLBCL samples are assigned to $6$ modules.

(3) is interesting. As we mentioned before, the DLBCL type is too large, and may not be a well-defined true type. Here, we see that the algorithm $\mathcal{I}$ assigned the DLBCL cell samples to $6$ modules. We will further analyse the divisions of the DLBCL samples using clinical data.

## Modules identified by $\mathcal{E}^2$

In Table 11, we describe the classification of the tumor types of lymphoma identified by our algorithm $\mathcal{E}^2$.

Figure 9 and S-Table 11 reveal the following properties:

(1) Modules $1$, $2$, $3$, $7$, $8$, $9$, $10$, and $11$ essentially correspond to transformed cell lines, activated B-like DLBCL, GC B-like DLBCL, activated blood B, resting/activated T, FL, resting blood B, and CLL, respectively.

(2) The DLBCL type is essentially divided into modules $2$, $3$, and $6$.

(3) Except for module $3$, which contains the subtype GC B-like DLBCL, every module is highly expressed by a significantly large set of genes.

(4) Module $2$ is the subtype activated B-like DLBCL and is highly expressed by a set of more than $300$ genes.

(5) Module $3$ contains the subtype GC B-like DLBCL (except for DLCL-0011) and is large. However, the module is well-expressed only by a set of less than $100$ genes. This finding could be caused by i) the expression of the subtype GC B-like by only a small set of genes or ii) an incomplete current gene expression array.

(6) Module $6$ contains a subset of DLBCL consisting of DLCL-0015, DLCL-0026, DLCL-0023, DLCL-0027, DLCL-0024, DLCL-0005, DLCL-0013, DLCL-0016, DLCL-0014, and DLCL-004, and its biological and medical classification is unknown. However, our two-dimensional gene map shows that the module is highly expressed by a set of more than $290$ genes.

(7) Module $4$ consists of DLCL-0004, DLCL-0029, DLCL-0008, Tonsil GC B, Tonsil GC Centroblasts, SUDHL6, and DLCL-0052, and it is a combination of GC B-like DLBCL, and germinal centre B. Our gene map shows that the module is highly expressed by a set of more than $300$ genes.

(8) Module $5$ consists of DLCL-0006, DLCL-0049, tonsil, DLCL-0039, lymph Node, and DLCL-0002, and it is a combination of activated B-like DLBCL and NL. lymph node/tonsil. Our gene map shows that the module is highly expressed by a set of more than $400$ genes, implying that, it is a biologically meaningful type.

(9) Module 8 is the resting/activated T, and it is highly expressed by a set of more than $1,800$ genes.

(10) Module 11 is the CLL, and it is highly expressed by a set of more than $450$ genes.

These results imply that modules 2, 3 and 6 could be new subtypes of DLBCL and modules 4 and 5 could be new subtypes of lymphoma. We will verify these results by clinical data analyses.

In Tables 12 and 13, we describe the top $10$ genes expressing the modules in Table 11 and the true types.

## Modules identified by $\mathcal{E}^3$

In Table 14, we describe the modules and submodules of cell samples of the lymphoma identified by our algorithm $\mathcal{E}^3$.

Figure 10 and S-Table 12 reveal the following results:

(1) Here, $13$ modules are defined. Module 1 is the same as the module 1 identified by the two-dimensional algorithm $\mathcal{E}^2$. The union of modules 2 and 3 is the activated B-like (except for DLCL-0012). The union of modules 4 and 7 is the GC B-like DLBCL (except for DLCL-0011). Modules 5 and 8 are the same as the modules 4 and 6 identified by $\mathcal{E}^2$, respectively. Module 6 represents the activated B-like DLBCL with NL. lymph node/tonsil. Modules 9, 10, 11, 12 and 13 represent the activated blood B, resting activated T, FL, resting blood B and CLL, respectively. Each module is divided into several submodules.

We use $X_i$ to denote module $i$, $Y_{i,j}$ to denote the $j$-th submodule of module $X_i$, and $B_{i,j}$ to denote the set of genes that express $Y_{i,j}$, which is marked at the left side of the boundary of Figure 10.

(2) As with acute leukaemia, our gene map shown in Figure 10 generates a high-definition, one-to-one map between the submodules $Y_{i,j}$ and the gene patterns $B_{i,j}$ for all $i$ and $j$.

(3) Subtype 3.2 consists of DLCL-0040 and DLCL-0017 and is remarkably expressed by a set of more than $350$ genes.

(4) Subtype 5.3 consists of tonsil GC B and tonsil GC centroblasts (germinal centre B) and is remarkably expressed by a set of more than $260$ genes.

(5) Subtype 10.1 consists of Blood T cells;Adult Naive CD4+ Unstimulated, Blood T cells;Adult Naive CD4+ I+P Stimulated, Cord Blood T cells;Neonatal Naive I+P Stimulated and is remarkably expressed by a set of more than $440$ genes.

(6) Subtype 10.2 consists of Blood T cells;Neonatal Naive CD4+ Unstimulated, Thymic T cells;Fetal CD4+ I+P Stimulated, Thymic T cells;Fetal CD4+ Unstimulated and is remarkably expressed by a set of more than $550$ genes.

In Tables 15 and 16, we describe the top $10$ genes expressing the submodules identified in Table 14.

| True type | Samples | Cell type |
|---|---|---|
| 1 | OCI Ly3, OCI Ly10, DLCL-0042, DLCL-0007, DLCL-0031, DLCL-0036;OCT, DLCL-0030, DLCL-0004, DLCL-0029, SUDHL6, DLCL-0008, DLCL-0052, DLCL-0034, DLCL-0051, DLCL-0011, DLCL-0032, DLCL-0006, DLCL-0049, DLCL-0039, DLCL-0001, DLCL-0018, DLCL-0037, DLCL-0010, DLCL-0015, DLCL-0026, DLCL-0005, DLCL-0023, DLCL-0027, DLCL-0024, DLCL-0013, DLCL-0002, DLCL-0016, DLCL-0020, DLCL-0003, DLCL-0014, DLCL-0048, DLCL-0033, DLCL-0025, DLCL-0040, DLCL-0017, DLCL-0028, DLCL-0012, DLCL-0021, OCI Ly1, DLCL-0041, DLCL-0009 | DLBCL |
| 2 | Tonsil GC B, Tonsil GC Centroblasts | Germinal centre B |
| 3 | Tonsil, Lymph Node | NL.lymph node/tonsil |
| 4 | Blood B cells;anti-IgM+CD40L low 48h, Blood B cells;anti-IgM+CD40L high 48h, Blood B cells;anti-IgM+CD40L 24h, Blood B cells;anti-IgM 24h, Blood B cells;anti-IgM+IL-4 24h, Blood B cells;anti-IgM+CD40L+IL-4 24h, Blood B cells;anti-IgM+IL-4 6h, Blood B cells;anti-IgM 6h, Blood B cells;anti-IgM+CD40L 6h, Blood B cells;anti-IgM+CD40L+IL-4 6h | Activated blood B |
| 5 | Blood T cells;Adult Naive CD4+ Unstimulated, Blood T cells;Adult Naive CD4+ I+P Stimulated, Cord Blood T cells;Neonatal Naive I+P Stimulated, Blood T cells;Neonatal Naive CD4+ Unstimulated, Thymic T cells;Fetal CD4+ Unstimulated, Thymic T cells;Fetal CD4+ I+P Stimulated | Resting/ activated T |
| 6 | WSU1, Jurkat, U937, OCI Ly12, OCI Ly13.2, SUDHL5 | Transformed cell lines |
| 7 | FL-9, FL-9;CD19+, FL-12;CD19+, FL-10;CD19+, FL-10, FL-11, FL-11;CD19+, FL-6;CD19+, FL-5;CD19+ | FL |
| 8 | Blood B cells;memory CD27+, Blood B cells;naive CD27-, Blood B cells, Cord Blood B cells | Resting blood B |
| 9 | CLL-60, CLL-68, CLL-9, CLL-14, CLL-51, CLL-65, CLL-71;Richter's, CLL-71, CLL-13, CLL-39, CLL-52 | CLL |

Table 8: True types of the cell samples of lymphoma.

| Module | Samples | Cell type |
|---|---|---|
| 1 | OCI Ly3, Thymic T cells;Fetal CD4+ Unstimulated, OCI Ly1, DLCL-0041, WSU1, OCI Ly13.2, U937, Jurkat, OCI Ly12, SUDHL5, OCI Ly10, DLCL-0042, DLCL-0007, DLCL-0031, DLCL-0036;OCT, DLCL-0017, DLCL-0025, DLCL-0028, DLCL-0040, DLCL-0021, DLCL-0012, DLCL-0014, DLCL-0003, DLCL-0010, DLCL-0018, DLCL-0030, DLCL-0015, DLCL-0037, DLCL-0052, DLCL-0051, SUDHL6, DLCL-0004, DLCL-0008, DLCL-0001, DLCL-0034, DLCL-0032, DLCL-0029, DLCL-0020, DLCL-0033, DLCL-0048, DLCL-0016, DLCL-0023, DLCL-0011, DLCL-0006, DLCL-0002, DLCL-0005, DLCL-0013, DLCL-0027, DLCL-0026, DLCL-0024, DLCL-0049, Lymph Node, Tonsil, DLCL-0039 | DLBCL, NL.lymph node/tonsil, Transformed cell lines, Resting/ activated T |
| 2 | Tonsil GC B, Tonsil GC Centroblasts, FL-9, FL-9;CD19+, FL-12;CD19+, FL-10;CD19+, FL-6;CD19+, FL-5;CD19+, DLCL-0009, FL-10, FL-11, FL-11;CD19+, CLL-65, Blood B cells;memory CD27+, Blood B cells;naive CD27-, Cord Blood B cells, Blood B cells, CLL-60, CLL-71;Richter's, CLL-9, CLL-68, CLL-51, CLL-71, CLL-14, CLL-13, CLL-52, CLL-39 | Germinal centre B, FL, DLBCL, Resting blood B, CLL |
| 3 | Blood B cells;anti-IgM+CD40L low 48h, Blood B cells;anti-IgM+CD40L 24h, Blood B cells;anti-IgM+CD40L high 48h, Blood B cells;anti-IgM 24h, Blood B cells;anti-IgM+CD40L+IL-4 24h, Blood B cells;anti-IgM+IL-4 24h, Blood B cells;anti-IgM+CD40L 6h, Blood B cells;anti-IgM 6h, Blood B cells;anti-IgM+IL-4 6h, Blood B cells;anti-IgM+CD40L+IL-4 6h | Activated blood B |
| 4 | Blood T cells;Adult Naive CD4+ Unstimulated, Thymic T cells;Fetal CD4+ I+P Stimulated, Blood T cells;Adult Naive CD4+ I+P Stimulated, Cord Blood T cells;Neonatal Naive I+P Stimulated, Blood T cells;Neonatal Naive CD4+ Unstimulated | Resting/ activated T |

Table 9: The modules of the cell samples of lymphoma identified by $\mathcal{M}$

| Module | Samples | Cell type |
|---|---|---|
| 1 | DLCL-0030, DLCL-0004, DLCL-0029, DLCL-0008, DLCL-0052, DLCL-0034, DLCL-0051, DLCL-0011, DLCL-0032, DLCL-0006, DLCL-0049, Tonsil, DLCL-0039, Lymph Node, DLCL-0001, DLCL-0018, DLCL-0037, DLCL-0010, DLCL-0015, DLCL-0026, DLCL-0005, DLCL-0023, DLCL-0027, DLCL-0024, DLCL-0013, DLCL-0002, DLCL-0016, DLCL-0020, DLCL-0003, DLCL-0014, DLCL-0048, DLCL-0033, DLCL-0012 | DLBCL, NL.lymph node/tonsil |
| 2 | Blood B cells;memory CD27+, Blood B cells;naive CD27-, Blood B cells, Cord Blood B cells, CLL-60, CLL-68, CLL-9, CLL-14, CLL-51, CLL-65, CLL-71;Richter's, CLL-71, CLL-13, CLL-39, CLL-52 | Resting blood B, CLL |
| 3 | Tonsil GC B, Tonsil GC Centroblasts, FL-9, FL-9;CD19+, FL-12;CD19+, FL-10;CD19+, FL-10, FL-11, FL-11;CD19+, FL-6;CD19+, FL-5;CD19+, DLCL-0009 | Germinal centre B, FL, DLBCL |
| 4 | Blood B cells;anti-IgM+CD40L low 48h, Blood B cells;anti-IgM+CD40L high 48h, Blood B cells;anti-IgM+CD40L 24h, Blood B cells;anti-IgM 24h, Blood B cells;anti-IgM+IL-4 24h, Blood B cells;anti-IgM+CD40L+IL-4 24h, Blood B cells;anti-IgM+IL-4 6h, Blood B cells;anti-IgM 6h, Blood B cells;anti-IgM+CD40L 6h, Blood B cells;anti-IgM+CD40L+IL-4 6h | Activated blood B |
| 5 | Blood T cells;Adult Naive CD4+ Unstimulated, Blood T cells;Adult Naive CD4+ I+P Stimulated, Cord Blood T cells;Neonatal Naive I+P Stimulated, Blood T cells;Neonatal Naive CD4+ Unstimulated, Thymic T cells;Fetal CD4+ Unstimulated, Thymic T cells;Fetal CD4+ I+P Stimulated | Resting/ activated T |
| 6 | OCI Ly1, WSU1, Jurkat, U937, OCI Ly12, OCI Ly13.2, SUDHL5, DLCL-0041 | Transformed cell lines, DLBCL |
| 7 | DLCL-0036;OCT, DLCL-0025, DLCL-0040, DLCL-0017, DLCL-0028, DLCL-0021 | DLBCL |
| 8 | OCI Ly3, OCI Ly10, DLCL-0042, DLCL-0007, DLCL-0031 | DLBCL |
| 9 | SUDHL6 | DLBCL |

Table 10: The modules of the cell samples of lymphoma identified by $\mathcal{I}$

| Module | Samples | Cell type |
|---|---|---|
| 1 | OCI Ly3, OCI Ly1, WSU1, Jurkat, U937, OCI Ly12, OCI Ly13.2, SUDHL5, DLCL-0041 | DLBCL, Transformed cell lines |
| 2 | OCI Ly10, DLCL-0042, DLCL-0007, DLCL-0031, DLCL-0036;OCT, DLCL-0025, DLCL-0040, DLCL-0017, DLCL-0028, DLCL-0021, DLCL-0012 | Activated B-like DLBCL (but DLCL-0012) |
| 3 | DLCL-0030, DLCL-0011, DLCL-0020, DLCL-0032, DLCL-0033, DLCL-0003, DLCL-0034, DLCL-0051, DLCL-0001, DLCL-0018, DLCL-0037, DLCL-0010 | GC B-like DLBCL (but DLCL-0011) |
| 4 | DLCL-0004, DLCL-0029, DLCL-0008, Tonsil GC B, Tonsil GC Centroblasts, SUDHL6, DLCL-0052 | GC B-like DLBCL, Germinal centre B |
| 5 | DLCL-0006, DLCL-0049, Tonsil, DLCL-0039, Lymph Node, DLCL-0002 | Activated B-like DLBCL, Nl. lymph node/tonsil |
| 6 | DLCL-0015, DLCL-0026, DLCL-0023, DLCL-0027, DLCL-0024, DLCL-0005, DLCL-0013, DLCL-0016, DLCL-0014, DLCL-0048 | DLBCL |
| 7 | Blood B cells;anti-IgM+CD40L low 48h, Blood B cells;anti-IgM+CD40L high 48h, Blood B cells;anti-IgM+CD40L 24h, Blood B cells;anti-IgM 24h, Blood B cells;anti-IgM+IL-4 24h, Blood B cells;anti-IgM+IL-4 6h, Blood B cells;anti-IgM 6h, Blood B cells;anti-IgM+CD40L 6h, Blood B cells;anti-IgM+CD40L+IL-4 6h, Blood B cells;anti-IgM+CD40L+IL-4 24h | Activated blood B |
| 8 | Blood T cells;Adult Naive CD4+ Unstimulated, Blood T cells;Adult Naive CD4+ I+P Stimulated, Cord Blood T cells;Neonatal Naive I+P Stimulated, Blood T cells;Neonatal Naive CD4+ Unstimulated, Thymic T cells;Fetal CD4+ Unstimulated, Thymic T cells;Fetal CD4+ I+P Stimulated | Resting/activated T |
| 9 | FL-9, FL-12;CD19+, FL-10;CD19+, FL-10, FL-11, FL-11;CD19+, FL-6;CD19+, FL-9;CD19+, FL-5;CD19+, DLCL-0009 | FL (with DLCL-0011) |
| 10 | Blood B cells;memory CD27+, Blood B cells, Cord Blood B cells, Blood B cells;naive CD27- | Resting blood B |
| 11 | CLL-60, CLL-68, CLL-9, CLL-14, CLL-51, CLL-71;Richter's, CLL-71, CLL-13, CLL-65, CLL-52, CLL-39 | CLL |

Table 11: Modules of cell samples of lymphoma identified by $\mathcal{E}^2$.

| Modules identified by $\mathcal{E}^2$ | Top 10 genes of modules identified by $\mathcal{E}^2$ | Top 10 genes of true types | Type |
|---|---|---|---|
| 1 | GENE784X, GENE696X, GENE753X, GENE785X, GENE595X, GENE694X, GENE692X, GENE750X, GENE688X, GENE276X | GENE595X, GENE784X, GENE753X, GENE275X, GENE785X, GENE276X, GENE783X, GENE544X, GENE694X, GENE786X | Transformed cell lines |
| 2 | GENE565X, GENE524X, GENE3944X, GENE636X, GENE3956X, GENE493X, GENE3753X, GENE722X, GENE712X, GENE513X | GENE1648X, GENE1636X, GENE1702X, GENE1610X, GENE1651X, GENE1641X, GENE1637X, GENE1607X, GENE1603X, GENE1631X | DLBCL |
| 3 | GENE1718X, GENE1720X, GENE1717X, GENE1719X, GENE3234X, GENE1731X, GENE3617X, GENE3361X, GENE1607X, GENE1763X | GENE1648X, GENE1636X, GENE1702X, GENE1610X, GENE1651X, GENE1641X, GENE1637X, GENE1607X, GENE1603X, GENE1631X | DLBCL |
| 4 | GENE740X, GENE3618X, GENE429X, GENE3326X, GENE742X, GENE3332X, GENE3330X, GENE741X, GENE3614X, GENE3232X, | GENE3325X, GENE194X, GENE3335X, GENE3326X, GENE3393X, GENE3344X, GENE3334X, GENE3694X, GENE3209X, GENE430X | Germinal centre B |
| 5 | GENE1658X, GENE1587X, GENE1653X, GENE1785X, GENE1659X, GENE1309X, GENE1663X, GENE1592X, GENE1665X, GENE1622X | GENE1793X, GENE1678X, GENE1665X, GENE1535X, GENE1653X, GENE3893X, GENE3474X, GENE3125X, GENE1563X, GENE3816X | Nl. lymph node/tonsil |
| 6 | GENE1651X, GENE1636X, GENE1641X, GENE1640X, GENE1637X, GENE1632X, GENE1760X, GENE1638X, GENE1424X, GENE1649X | GENE1648X, GENE1636X, GENE1702X, GENE1610X, GENE1651X, GENE1641X, GENE1637X, GENE1607X, GENE1603X, GENE1631X | DLBCL |

Table 12: Top 10 genes of modules of the lymphoma identified by $\mathcal{E}^2$ and true types-1

| Modules identified by $\mathcal{E}^2$ | Top 10 genes of modules identified by $\mathcal{E}^2$ | Top 10 genes of true types | Type |
|---|---|---|---|
| 7 | GENE1202X, GENE1172X, GENE2515X, GENE1229X, GENE2758X, GENE1171X, GENE1216X, GENE1223X, GENE1162X, GENE1214X | GENE1202X, GENE1172X, GENE2515X, GENE1229X, GENE2758X, GENE1171X, GENE1216X, GENE1223X, GENE1162X, GENE1214X | Activated blood B |
| 8 | GENE1316X, GENE1305X, GENE2712X, GENE3002X, GENE1995X, GENE2820X, GENE1996X, GENE2821X, GENE1329X, GENE2009X | GENE1316X, GENE1305X, GENE2712X, GENE3002X, GENE1995X, GENE2820X, GENE1996X, GENE2821X, GENE1329X, GENE2009X | Resting/ activated T |
| 9 | GENE3286X, GENE3285X, GENE3321X, GENE3320X, GENE2309X, GENE3705X, GENE2335X, GENE2415X, GENE3318X, GENE3314X | GENE3286X, GENE3285X, GENE3320X, GENE3321X, GENE2309X, GENE2335X, GENE2409X, GENE2310X, GENE2415X, GENE3275X | FL |
| 10 | GENE1887X, GENE1363X, GENE1367X, GENE1886X, GENE2487X, GENE1355X, GENE2401X, GENE1362X, GENE3084X, GENE2486X | GENE1887X, GENE1363X, GENE1367X, GENE1886X, GENE2487X, GENE1355X, GENE2401X, GENE1362X, GENE2220X, GENE1373X | Resting blood B |
| 11 | GENE2244X, GENE2496X, GENE2390X, GENE2395X, GENE2392X, GENE2407X, GENE2203X, GENE2402X, GENE2328X, GENE2365X | GENE2244X, GENE2496X, GENE2390X, GENE2395X, GENE2392X, GENE2407X, GENE2203X, GENE2402X, GENE2328X, GENE2365X | CLL |

Table 13: Top 10 genes of modules of the lymphoma identified by $\mathcal{E}^2$ and true types-2

| Module | Submodule | Cell type |
|---|---|---|
| 1 | OCI Ly3, SUDHL5 | |
| | OCI Ly1, DLCL-0041, WSU1, Jurkat | |
| | U937, OCI Ly12, OCI Ly13.2 | |
| 2 | OCI Ly10, DLCL-0042 | |
| | DLCL-0007, DLCL-0031 | |
| 3 | DLCL-0036;OCT, DLCL-0025, DLCL-0028 | |
| | DLCL-0040, DLCL-0017 | |
| | DLCL-0021, DLCL-0012 | |
| 4 | DLCL-0030, DLCL-0032, DLCL-0051 | |
| | DLCL-0011, DLCL-0020 | |
| | DLCL-0033, DLCL-0034, DLCL-0003 | |
| 5 | DLCL-0004, DLCL-0029 | |
| | DLCL-0008, DLCL-0052, SUDHL6 | |
| | Tonsil GC B, Tonsil GC Centroblasts | Germinal centre B |
| 6 | DLCL-0006, DLCL-0002 | |
| | DLCL-0049, Tonsil, DLCL-0039, Lymph Node | |
| 7 | DLCL-0001, DLCL-0018 | |
| | DLCL-0037, DLCL-0010 | |
| 8 | DLCL-0015, DLCL-0005, DLCL-0013 | |
| | DLCL-0026, DLCL-0027, DLCL-0024, DLCL-0023 | |
| | DLCL-0016, DLCL-0014, DLCL-0048 | |
| 9 | Blood B cells;anti-IgM+CD40L low 48h, Blood B cells;anti-IgM+CD40L high 48h, Blood B cells;anti-IgM+CD40L 24h | |
| | Blood B cells;anti-IgM 24h, Blood B cells;anti-IgM+IL-4 24h, Blood B cells;anti-IgM+CD40L+IL-4 24h | |
| | Blood B cells;anti-IgM+IL-4 6h, Blood B cells;anti-IgM 6h, Blood B cells;anti-IgM+CD40L 6h, Blood B cells;anti-IgM+CD40L+IL-4 6h | |
| 10 | Blood T cells;Adult Naive CD4+ Unstimulated, Blood T cells;Adult Naive CD4+ I+P Stimulated, Cord Blood T cells;Neonatal Naive I+P Stimulated | |
| | Blood T cells;Neonatal Naive CD4+ Unstimulated, Thymic T cells;Fetal CD4+ I+P Stimulated, Thymic T cells;Fetal CD4+ Unstimulated | |
| 11 | FL-9, FL-9;CD19+, FL-11, FL-11;CD19+ | |
| | FL-12;CD19+, FL-10;CD19+, FL-10 | |
| | FL-6;CD19+, FL-5;CD19+, DLCL-0009 | |
| 12 | Blood B cells;memory CD27+, Blood B cells;naive CD27- | |
| | Blood B cells, Cord Blood B cells | |
| 13 | CLL-60, CLL-68, CLL-65 | |
| | CLL-9, CLL-51, CLL-71;Richter's, CLL-13 | |
| | CLL-14, CLL-71, CLL-52, CLL-39 | |

Table 14: The modules and submodules of cell samples of lymphoma identified by $\mathcal{E}^3$.

| Submodule | Top 10 genes of submodules identified by $\mathcal{E}^3$ |
|---|---|
| 1.1 | GENE784X, GENE258X, GENE717X, GENE696X, GENE753X, GENE945X, GENE785X, GENE169X, GENE170X, GENE2752X |
| 1.2 | GENE686X, GENE331X, GENE330X, GENE745X, GENE424X, GENE410X, GENE409X, GENE726X, GENE408X, GENE2970X |
| 1.3 | GENE504X, GENE1578X, GENE783X, GENE661X, GENE275X, GENE778X, GENE786X, GENE1043X, GENE798X, GENE276X |
| 2.1 | GENE824X, GENE364X, GENE3950X, GENE815X, GENE1019X, GENE825X, GENE435X, GENE3944X, GENE3626X, GENE1253X |
| 2.2 | GENE848X, GENE545X, GENE687X, GENE3371X, GENE657X, GENE690X, GENE608X, GENE628X, GENE655X, GENE3404X |
| 3.1 | GENE3956X, GENE3619X, GENE3958X, GENE3620X, GENE1037X, GENE3839X, GENE3955X, GENE3484X, GENE1478X, GENE3611X |
| 3.2 | GENE3753X, GENE1555X, GENE3941X, GENE518X, GENE473X, GENE1671X, GENE3933X, GENE3754X, GENE1614X, GENE963X |
| 3.3 | GENE881X, GENE986X, GENE829X, GENE3225X, GENE3224X, GENE3476X, GENE60X, GENE1582X, GENE360X, GENE1581X |
| 4.1 | GENE3917X, GENE1151X, GENE3918X, GENE1780X, GENE3253X, GENE3235X, GENE3696X, GENE1572X, GENE3018X, GENE3019X |
| 4.2 | GENE1731X, GENE1688X, GENE1740X, GENE1598X, GENE914X, GENE1594X, GENE1596X, GENE1595X, GENE1728X, GENE3312X |
| 4.3 | GENE3607X, GENE371X, GENE1732X, GENE3387X, GENE1826X, GENE3553X, GENE859X, GENE3856X, GENE1750X, GENE1751X |
| 5.1 | GENE3815X, GENE3296X, GENE2031X, GENE3428X, GENE3395X, GENE3425X, GENE3538X, GENE4002X, GENE4001X, GENE3649X |
| 5.2 | GENE113X, GENE741X, GENE742X, GENE3310X, GENE3530X, GENE126X, GENE1066X, GENE674X, GENE3304X, GENE461X |
| 5.3 | GENE3325X, GENE194X, GENE3335X, GENE3326X, GENE3393X, GENE3344X, GENE3334X, GENE3694X, GENE3209X, GENE430X |
| 6.1 | GENE1225X, GENE1625X, GENE1226X, GENE1131X, GENE1601X, GENE1610X, GENE611X, GENE1612X, GENE1227X, GENE1658X |
| 6.2 | GENE1587X, GENE1653X, GENE1936X, GENE3468X, GENE3844X, GENE1785X, GENE1592X, GENE1665X, GENE1491X, GENE3851X |
| 7.1 | GENE3927X, GENE1377X, GENE1378X, GENE3926X, GENE1510X, GENE3249X, GENE1723X, GENE1670X, GENE1076X, GENE3355X |
| 7.2 | GENE3855X, GENE3338X, GENE100X, GENE554X, GENE3848X, GENE451X, GENE1718X, GENE3581X, GENE1674X, GENE635X |

Table 15: Top 10 genes of submodules of the lymphoma identified by $\mathcal{E}^3$-1

| Submodule | Top 10 genes of the submodules identified by $\mathcal{E}^3$ |
|---|---|
| 8.1 | GENE1650X, GENE1632X, GENE1639X, GENE1583X, GENE1689X, GENE1748X, GENE1585X, GENE1649X, GENE1603X, GENE1620X |
| 8.2 | GENE1651X, GENE1415X, GENE1640X, GENE1636X, GENE1641X, GENE1406X, GENE1411X, GENE1633X, GENE1637X, GENE1416X |
| 8.3 | GENE1666X, GENE1814X, GENE1424X, GENE1820X, GENE1752X, GENE1513X, GENE946X, GENE1909X, GENE1681X, GENE391X |
| 9.1 | GENE1505X, GENE1504X, GENE2545X, GENE105X, GENE1223X, GENE1230X, GENE106X, GENE142X, GENE1229X, GENE571X |
| 9.2 | GENE563X, GENE2880X, GENE2098X, GENE1193X, GENE2535X, GENE89X, GENE277X, GENE82X, GENE892X, GENE965X |
| 9.3 | GENE1202X, GENE1172X, GENE1249X, GENE1205X, GENE2515X, GENE1175X, GENE2834X, GENE1171X, GENE1161X, GENE2292X |
| 10.1 | GENE2757X, GENE2304X, GENE1330X, GENE1329X, GENE1316X, GENE324X, GENE1328X, GENE1315X, GENE1204X, GENE1292X |
| 10.2 | GENE2869X, GENE2722X, GENE2913X, GENE1326X, GENE1978X, GENE2663X, GENE1995X, GENE2868X, GENE1992X, GENE2915X |
| 11.1 | GENE3275X, GENE3200X, GENE3199X, GENE3274X, GENE3198X, GENE4014X, GENE4011X, GENE4015X, GENE4012X, GENE2416X |
| 11.2 | GENE3286X, GENE3285X, GENE3320X, GENE3227X, GENE3321X, GENE3226X, GENE2309X, GENE3288X, GENE2154X, GENE3068X |
| 11.3 | GENE3329X, GENE1916X, GENE3314X, GENE3793X, GENE3023X, GENE3864X, GENE3284X, GENE3021X, GENE1863X, GENE3313X |
| 12.1 | GENE1360X, GENE1371X, GENE1362X, GENE1361X, GENE1358X, GENE1182X, GENE1886X, GENE2220X, GENE2401X, GENE1368X |
| 12.2 | GENE2066X, GENE3084X, GENE2488X, GENE2486X, GENE2487X, GENE1373X, GENE1356X, GENE2489X, GENE2365X, GENE3083X |
| 13.1 | GENE2184X, GENE2251X, GENE2407X, GENE2203X, GENE2236X, GENE2183X, GENE2221X, GENE2247X, GENE2246X, GENE2174X |
| 13.2 | GENE2162X, GENE2196X, GENE2238X, GENE2385X, GENE2318X, GENE2345X, GENE2389X, GENE2501X, GENE3520X, GENE2383X |
| 13.3 | GENE2496X, GENE2390X, GENE2391X, GENE2502X, GENE2328X, GENE2423X, GENE2392X, GENE2398X, GENE2393X, GENE2394X |

Table 16: Top 10 genes of the submodules of the lymphoma identified by $\mathcal{E}^3$-2

# Multi-tissues

## True types

Table 17 describes the true types of the multi-tissues.

| True type | Samples | Cell type |
|---|---|---|
| 1 | BR_BR8T, BR_BR10T, BR_BR14T, BR_BR16T, BR_BR17T, BR_BR20T, BR_BR6T, BR_BR15T, BR_BR21T, BR_BR29T, BR_BR31T, BR_BR32T, BR_U1, BR_U16, BR_UX7, BR_UX8, BR_UX19, BR_B24T, BR_B46T, BR_B30T, BR_B34T, BR_B36T, BR_B37T, BR_B38T, BR_B39T, BR_B41T | Br |
| 2 | PR_PR29T, PR_PR13BT, PR_PR8T, PR_PR21T, PR_PR27T, PR_PR9T, PR_PR7T, PR_PR19T, PR_PR5T, PR_PR24T, PR_U40, PR_U41, PR_PR4, PR_PR3, PR_PR1, PR_PR10, PR_PR22, PR_PR12, PR_PR31, PR_PR30, PR_PR26, PR_PR16, PR_PR23, PR_PR6, PR_PR11, PR_PR17 | Pr |
| 3 | LU_A_LU_A31T, LU_A_LU_A34T, LU_A_LU_A39T, LU_A_LU_A8T, LU_A_LU_A20T, LU_A_LU_A5T, LU_A_LU_A6T, LU_A_LU_A17T, LU_A_LU_A18T, LU_S_LU_S19T, LU_S_LU_S24T, LU_S_LU_S25T, LU_S_LU_S12T, LU_S_LU_S13T, LU_S_LU_S14T, LU_S_LU_S11T, LU_S_LU_S7T, LUA_U17, LUA_UX4, LUA_LU40T, LUA_LU44T, LUA_LU33T, LUS_U2, LUS_U19, LUS_LU41T, LUS_LU30T, LUS_LU26T, LUS_LU36T | Lu |
| 4 | CO_CO7T, CO_CO9T, CO_CO42T, CO_CO15T, CO_CO40T, CO_CO27T, CO_CO30T, CO_CO32T, CO_CO20T, CO_CO24T, CO_CO8T, CO_U6, CO_U12, CO_CO14T, CO_CO21T, CO_CO23T, CO_CO5T, CO_CO43T, CO_CO44T, CO_CO49T, CO_CO51T, CO_CO56T, CO_CO61T | Co |

Table 17: The true types of the multi-tissues

## Modules identified by $\mathcal{M}$

Table 18 describes the modules of the multi-tissues identified by $\mathcal{M}$.

## Modules identified by $\mathcal{I}$

Table 19 describes the modules of the multi-tissues identified by $\mathcal{I}$.

## Modules identified by $\mathcal{E}^2$

In Table 20, we describe the classification of modules of the multi-tissues identified by our algorithm $\mathcal{E}^2$.

| Module | Samples | Cell type |
|---|---|---|
| 1 | BR_BR8T, BR_BR14T, BR_BR16T, BR_BR20T, BR_BR6T, BR_U16, BR_BR15T, BR_BR32T, BR_UX8, BR_B38T, BR_B36T, BR_BR29T, BR_B34T, BR_BR17T, BR_UX19, BR_B24T, BR_BR21T, BR_B30T, BR_B37T, BR_B39T, LU_A_LU_A17T | Br, Lu |
| 2 | BR_BR10T, BR_BR31T, BR_B41T, CO_CO23T, CO_CO61T, CO_CO42T, CO_CO56T, CO_CO44T, CO_CO8T, CO_CO27T, CO_CO5T, CO_CO14T, CO_CO32T, CO_CO43T, CO_CO49T, CO_CO51T, CO_CO21T, CO_U12, LUA_U17, BR_U1, CO_U6, CO_CO30T, CO_CO40T, CO_CO9T, CO_CO15T, CO_CO7T, CO_CO24T, CO_CO20T, LU_S_LU_S13T | Br, Co, Lu |
| 3 | BR_UX7, BR_B46T | Br |
| 4 | PR_PR29T, PR_PR13BT, PR_PR8T, PR_PR21T, PR_PR24T, PR_U40, PR_PR17, PR_PR9T, PR_PR4, PR_PR12, PR_PR11, PR_PR7T, PR_PR1, PR_PR23, PR_PR19T, PR_PR10, PR_PR27T, PR_PR22, PR_PR31, PR_PR3, PR_PR5T, PR_PR30, PR_PR6, PR_PR16, PR_PR26, PR_U41 | Pr |
| 5 | LU_A_LU_A31T, LU_A_LU_A34T, LU_S_LU_S19T, LU_S_LU_S24T, LU_S_LU_S12T, LUS_LU30T, LUS_U2, LU_S_LU_S11T, LU_S_LU_S25T, LUS_U19, LU_S_LU_S14T, LU_S_LU_S7T, LUS_LU41T, LUS_LU36T, LUS_LU26T, LUA_LU44T, LU_A_LU_A39T, LU_A_LU_A20T, LUA_UX4, LUA_LU33T, LUA_LU40T, LU_A_LU_A6T, LU_A_LU_A18T, LU_A_LU_A8T, LU_A_LU_A5T | Lu |

Table 18: The modules of the multi-tissues identified by $\mathcal{M}$

In Table 21, we describe the top $10$ genes expressing the modules identified by $\mathcal{E}^2$ and the true types of the multi-tissue.

## Modules identified by $\mathcal{E}^3$

In Table 22, we describe the modules and submodules of cell samples of the multi-tissues identified by our algorithm $\mathcal{E}^3$.

Figure 15 and S-Table 21 reveal the following results:

(1) Module $1$ is essentially of the type BR and consists of $4$ distinguishable submodules as follows:

1.1: BR_BR8T, BR_B30T, BR_B37T, BR_B46T, BR_B39T; 1.2: BR_BR10T, BR_B41T, BR_BR31T, BR_BR21T; 1.3: BR_B34T, BR_B36T, BR_BR16T, BR_B38T, BR_BR17T, BR_B24T, BR_BR32T, BR_BR29T; and 1.4: BR_UX8, BR_BR15T, BR_UX19, BR_BR14T, BR_BR6T, BR_BR20T.

(2) Module $2$ is of the type CO and consists of $3$ distinguishable submodules as follows:

| Module | Samples | Cell type |
|---|---|---|
| 1 | BR_BR10T, BR_BR31T, BR_U1, BR_B41T, LU_S_LU_S13T, LU-A_U17, CO_CO7T, CO_CO9T, CO_CO42T, CO_CO15T, CO_CO40T, CO_CO27T, CO_CO30T, CO_CO32T, CO_CO20T, CO_CO24T, CO_CO8T, CO_U6, CO_U12, CO_CO14T, CO_CO21T, CO_CO23T, CO_CO5T, CO_CO43T, CO_CO44T, CO_CO49T, CO_CO51T, CO_CO56T, CO_CO61T | Br, Lu, Co |
| 2 | PR_PR29T, PR_PR13BT, PR_PR8T, PR_PR21T, PR_PR27T, PR_PR9T, PR_PR7T, PR_PR19T, PR_PR5T, PR_PR24T, PR_U40, PR_U41, PR_PR4, PR_PR3, PR_PR1, PR_PR10, PR_PR22, PR_PR12, PR_PR31, PR_PR30, PR_PR26, PR_PR16, PR_PR23, PR_PR6, PR_PR11, PR_PR17 | Pr |
| 3 | LU_A_LU_A31T, LU_A_LU_A34T, LU_A_LU_A39T, LU_A_LU_A8T, LU_A_LU_A20T, LU_A_LU_A5T, LU_A_LU_A6T, LU_A_LU_A18T, LU_S_LU_S19T, LU_S_LU_S24T, LU_S_LU_S25T, LU_S_LU_S12T, LU_S_LU_S14T, LU_S_LU_S11T, LU_S_LU_S7T, LUA_UX4, LUA_LU40T, LUA_LU44T, LUA_LU33T, LUS_U2, LUS_U19, LUS_LU41T, LUS_LU30T, LUS_LU26T, LUS_LU36T | Lu |
| 4 | BR_BR14T, BR_BR16T, BR_BR17T, BR_BR20T, BR_BR6T, BR_BR15T, BR_BR21T, BR_BR29T, BR_BR32T, BR_UX8, BR_UX19, BR_B24T, BR_B34T, BR_B36T, BR_B38T | Br |
| 5 | BR_BR8T, BR_UX7, BR_B46T, BR_B30T, BR_B37T, BR_B39T, LU_A_LU_A17T | Br, Lu |
| 6 | BR_U16 | Br |

Table 19: The modules of the multi-tissues identified by $\mathcal{I}$

2.1: BR_U1, LUA_U17, CO_CO14T, CO_CO21T, CO_U12, CO_CO23T, CO_CO49T, CO_CO51T; 2.2: CO_CO7T, CO_CO20T, CO_CO24T, CO_CO40T, CO_CO9T, CO_CO30T, CO_CO15T, CO_CO8T, CO_U6; and 2.3: CO_CO32T, CO_CO56T, CO_CO27T, CO_CO5T, LU_S_LU_S13T, CO_CO44T, CO_CO43T, CO_CO61T, CO_CO42T.

(3) Modules 3, 4 and 7 are singletons and consist of BR_U16, BR_UX7 and LU_A_LU_A17T, respectively.

(4) Module 5 is of the PR type and consists of 3 distinguishable submodules as follows:

5.1: PR_PR29T, PR_PR31, PR_PR6, PR_PR16, PR_PR26, PR_U41, PR_PR19T, PR_PR27T, PR_PR30; 5.2: PR_PR13BT, PR_PR8T, PR_PR7T, PR_PR1, PR_PR5T, PR_PR3, PR_PR23, PR_PR10, PR_PR22; and 5.3: PR_PR21T, PR_PR24T, PR_U40, PR_PR17, PR_PR9T, PR_PR4, PR_PR11, PR_PR12.

(5) Module 6 is of the type LU and consists of 4 distinguishable submodules as follows:

| Module | Samples | Cell type |
|---|---|---|
| 1 | BR_BR8T, BR_BR10T, BR_B41T, BR_BR31T, BR_BR21T, BR_B30T, BR_B37T, BR_B46T, BR_B39T, BR_B34T, BR_B36T, BR_UX8, BR_BR16T, BR_B38T, BR_BR32T, BR_BR17T, BR_BR29T, BR_BR20T, BR_BR15T, BR_B24T, BR_UX19, BR_BR6T, BR_BR14T, BR_U16 | BR (missing BR_U1, BR_UX7) |
| 2 | BR_U1, LUA_U17, CO_CO7T, CO_CO20T, CO_CO40T, CO_CO24T, CO_CO9T, CO_CO30T, CO_CO8T, CO_CO15T, CO_U6, CO_CO14T, CO_CO51T, CO_CO32T, CO_CO49T, CO_CO43T, CO_CO21T, CO_CO61T, CO_CO23T, CO_U12, CO_CO42T, CO_CO56T, CO_CO27T, CO_CO5T, CO_CO44T, LU_S_LU_S13T | CO (but BR_U1, LU_S_LU_S13T) |
| 3 | BR_UX7, LU_A_LU_A31T, LU_A_LU_A34T, LU_A_LU_A39T, LU_S_LU_S19T, LU_S_LU_S24T, LUS_U2, LUS_LU30T, LU_S_LU_S11T, LU_S_LU_S12T, LU_S_LU_S25T, LUS_U19, LU_S_LU_S14T, LUS_LU41T, LU_S_LU_S7T, LUS_LU26T, LUS_LU36T, LUA_LU44T, LU_A_LU_A20T, LUA_UX4, LUA_LU33T, LU-A_LU40T, LU_A_LU_A6T, LU_A_LU_A18T, LU_A_LU_A8T, LU_A_LU_A5T, LU_A_LU_A17T | LU (but BR_UX7, missing LU_S_LU_S13T) |
| 4 | PR_PR29T, PR_PR13BT, PR_PR8T, PR_PR21T, PR_PR19T, PR_PR24T, PR_U40, PR_PR17, PR_PR30, PR_PR12, PR_PR4, PR_PR9T, PR_PR11, PR_PR23, PR_PR7T, PR_PR1, PR_PR22, PR_PR10, PR_PR27T, PR_PR5T, PR_PR31, PR_PR3, PR_PR6, PR_PR16, PR_PR26, PR_U41 | PR |

Table 20: The modules of cell samples of multi-tissue identified by $\mathcal{E}^2$.

6.1: LU_A_LU_A31T, LU_A_LU_A18T, LUA_LU40T, LU_A_LU_A8T, LUA_LU33T, LU_A_LU_A34T, LU_A_LU_A20T, LU_A_LU_A6T; 6.2: LU_A_LU_A39T, LUS_LU26T, LUA_UX4, LU_S_LU_S14T;
6.3: LU_S_LU_S19T, LUS_LU36T, LUA_LU44T, LU_A_LU_A5T; and 6.4: LU_S_LU_S24T, LUS_U2, LUS_LU30T, LU_S_LU_S11T, LU_S_LU_S12T, LU_S_LU_S25T, LUS_U19, LUS_LU41T, LU_S_LU_S7T.

(6) Each submodule is defined by a set of a significantly large number of genes.

(7) Every gene pattern defining a submodule fails to define any other module or submodule.

(8) The three cells, BR_U16, BR_UX7 and LU_A_LU_A17T are expressed by sets of more than $1,500$, $300$ and $300$ genes, respectively.

| Modules identified by $\mathcal{E}^2$ | Top 10 genes of modules identified by $\mathcal{E}^2$ | Top 10 genes of true types | Type |
|---|---|---|---|
| 1 | CACYBP, MFAP2, ARID1A, RHOG, PRKCSH, UBN1, TANK, CCBL1, CALM2, HSPB2 | CACYBP, MFAP2, RHOG, ARID1A, TANK, ATP2B3, PRKCSH, CALM2, CCBL1, HSPB2 | BR |
| 2 | KRT8, ELF3, TMS-B4X, CDH17, HSPD1, RPS21, SSBP1, RPLP0, NP, JTV1 | ELF3, CDH17, KRT8, HSPD1, RPS21, JTV1, XK, RPLP0, TSPAN8, PLS1 | CO |
| 3 | IGL@, IGKC, IGH@, IGHA1, ANXA2, GSTP1, FTL, LOC91316, SFN, S100A11 | IGL@, IGKC, IGH@, IGHA1, GSTP1, RAB6IP1, SFN, S100A11, FTL, LOC91316 | LU |
| 4 | KLK3, CIRBP, KLK2, FOXA1, PDLIM5, SC5DL, LRPAP1, SFRS5, SNRPN, RPS4Y1 | KLK3, CIRBP, KLK2, FOXA1, PDLIM5, SC5DL, LRPAP1, SFRS5, SNRPN, RPS4Y1 | PR |

Table 21: Top 10 genes of modules of the multi-tissue identified by $\mathcal{E}^2$ and true types

These results demonstrate that our three-dimensional gene map shown in Figure 15 provides a high-definition, one-to-one map from the submodules of true cell types to the gene expression patterns of the multi-tissues and indicates that BR_U16, BR_UX7 and LU_A_LU_A17T are remarkable cells that may play special roles in the classification of multi-tissues.

In table 23, we describe the top 10 genes expressing the submodules of the multi-tissues identified by $\mathcal{E}^3$ in Table 22.

| Module | Submodule | Cell type |
|---|---|---|
| 1 | BR_BR8T, BR_B30T, BR_B37T, BR_B46T, BR_B39T | |
| | BR_BR10T, BR_B41T, BR_BR31T, BR_BR21T | |
| | BR_B34T, BR_B36T, BR_BR16T, BR_B38T, BR_BR17T, BR_B24T, BR_BR32T, BR_BR29T | |
| | BR_UX8, BR_BR15T, BR_UX19, BR_BR14T, BR_BR6T, BR_BR20T | |
| 2 | BR_U1, LUA_U17, CO_CO14T, CO_CO21T, CO_U12, CO_CO23T, CO_CO49T, CO_CO51T | |
| | CO_CO7T, CO_CO20T, CO_CO24T, CO_CO40T, CO_CO9T, CO_CO30T, CO_CO15T, CO_CO8T, CO_U6 | |
| | CO_CO32T, CO_CO56T, CO_CO27T, CO_CO5T, LU_S_LU_S13T, CO_CO44T, CO_CO43T, CO_CO61T, CO_CO42T | |
| 3 | BR_U16 | |
| 4 | BR_UX7 | |
| 5 | PR_PR29T, PR_PR31, PR_PR6, PR_PR16, PR_PR26, PR_U41, PR_PR19T, PR_PR27T, PR_PR30 | |
| | PR_PR13BT, PR_PR8T, PR_PR7T, PR_PR1, PR_PR5T, PR_PR3, PR_PR23, PR_PR10, PR_PR22 | |
| | PR_PR21T, PR_PR24T, PR_U40, PR_PR17, PR_PR9T, PR_PR4, PR_PR11, PR_PR12 | |
| 6 | LU_A_LU_A31T, LU_A_LU_A18T, LUA_LU40T, LU_A_LU_A8T, LUA_LU33T, LU_A_LU_A34T, LU_A_LU_A20T, LU_A_LU_A6T | |
| | LU_A_LU_A39T, LUS_LU26T, LUA_UX4, LU_S_LU_S14T | |
| | LU_S_LU_S19T, LUS_LU36T, LUA_LU44T, LU_A_LU_A5T | |
| | LU_S_LU_S24T, LUS_U2, LUS_LU30T, LU_S_LU_S11T, LU_S_LU_S12T, LU_S_LU_S25T, LUS_U19, LUS_LU41T, LU_S_LU_S7T | |
| 7 | LU_A_LU_A17T | |

Table 22: The modules and submodules of cell samples of multi-tissue identified by $\mathcal{E}^3$.

| Submodule | Top 10 genes of the submodules identified by $\mathcal{E}^3$ |
|---|---|
| 1.1 | ATR, TANK, C6orf62, ZFR, DKFZP564F0522, NCBP2, SMNDC1, HNRPA2B1, IFRD1, TPD52L2 |
| 1.2 | SFRS1, CDK3, HRMT1L2, RPS27A, RPL7, RPL13A, LYRIC, PALM, HNRPM, RBMX |
| 1.3 | GATA3, CDH11, FRAG1, LUM, POSTN, C RY2, C-SPG2, FN1, LMAN2, ZNF142 |
| 1.4 | CD164, ZFHX1B, NOTCH4, C2orf23, COL18A1, PDGFRB, XBP1, SNAPC2, COL5A2, PIP |
| 2.1 | CLDN4, PTK2, CLDN3, RPL14, CASK, EIF3S9, ADR-M1, CEACAM5, ILVBL, ELF4 |
| 2.2 | ELF3, EEF1A1, TMSB4X, TRIM31, XK, KRT20, H-SPD1, LGALS3, SLC25A5, EBP |
| 2.3 | RPS19, RPS3, RPL18, RPSA, HNRPA1, RPS21, SLC39A14, RPS23, RPLP0, SLC35D2 |
| 3 | 2'-PDE, 384D8-2, 76P, AANAT, ABCA1, ABCA3, ABCC8, ABCF2, ABHD14A, ABI2 |
| 4 | AGTRL1, BST2, BTK, CAT, CCL8, CCNE1, CD24, CHI3L1, CHRNB2, CLPP |
| 5.1 | KLK3, RPL10A, RPS4Y1, RPL29, RPL3, KLK2, M-LLT2, FOXA1, FLJ30092, RPL32 |
| 5.2 | ILK, MALT1, PPP1R12A, CBX7, MYH11, ACTA2, EP-S15, DMN, MYLK, PPP1R12B |
| 5.3 | GABARAP, VCP, CIRBP, WDR23, NDUFV2, NDUF-B7, NCOA2, MGST2, PARK7, RASSF3 |
| 6.1 | IGHA1, FBXL11, POU2AF1, TNFRSF7, CD6, C4BPA, NFKBIA, SFTPB, EVER1, ADCY7 |
| 6.2 | FTL, AKR1C1, CES1, RIT1, TALDO1, SPP1, GCLM, ANXA2, TXNRD1, GSTP1 |
| 6.3 | ENPP2, PRG1, IGHM, TRBC1, CD53, EVI2B, EMP3, CD48, CD3Z, FGL2 |
| 6.4 | SFN, KRT5, KRT6A, DR1, EIF4G1, PKM2, AP2M1, P-KP1, ENO1, PGAM1 |
| 7 | ATP6V0D1, BPGM, C10orf10, CFH, CYP24A1, ENTH, F8A1, GPX1, GRN, HCLS1 |

Table 23: Top 10 genes of submodules of the multi-tissue identified by $\mathcal{E}^3$

# Lung Cancer

## True types

Table 24 describes the true types of lung cancer.

| True type | Samples | Cell type |
|---|---|---|
| 1 | AD043, AD111, AD114, AD115, AD118, AD119, AD120, AD122, AD123, AD127, AD130, AD131, AD136, AD157, AD158, AD159, AD162, AD163, AD164, AD167, AD169, AD170, AD172, AD173, AD177, AD178, AD179, AD183, AD185, AD186, AD187, AD188, AD201, AD202, AD203, AD207, AD208, AD210, AD212, AD213, AD218, AD221, AD224, AD225, AD226, AD228, AD230, AD232, AD234, AD236, AD238, AD239, AD240, AD241, AD243, AD247, AD249, AD250, AD252, AD253, AD255, AD258, AD259, AD260, AD261, AD262, AD266, AD267, AD268, AD269, AD275, AD276, AD277, AD283, AD285, AD287, AD294, AD295, AD296, AD299, AD301, AD302, AD304, AD305, AD308, AD309, AD311, AD313, AD314, AD315, AD317, AD318, AD320, AD323, AD327, AD330, AD331, AD332, AD334, AD335, AD336, AD337, AD338, AD340, AD341, AD346, AD347, AD350, AD351, AD352, AD353, AD355, AD356, AD360, AD361, AD362, AD363, AD366, AD367, AD368, AD370, AD374, AD375, AD379, AD382, AD383, AD384, AD10, AD15, AD16, AD18, AD19, AD1, AD2, AD31, AD3, AD4, AD5, AD7 | AD |
| 2 | NL1179, NL1675, NL1698, NL1884, NL2378, NL2562, NL268, NL279, NL3104, NL3681, NL4083, NL4353, NL504, NL6084, NL6853, NL6943, NL7530 | NL |
| 3 | SQ1174, SQ1670, SQ2557, SQ2572, SQ2921, SQ3197, SQ3529, SQ3624, SQ4172, SQ4389, SQ5897, SQ6147, SQ7324, SQ10, SQ13, SQ14, SQ20, SQ4, SQ5, SQ6, SQ8 | SQ |
| 4 | COID1429, COID2260, COID3580, COID4385, COID4518, COID9794, COID10, COID11, COID12, COID13, COID14, COID16, COID18, COID3, COID4, COID5, COID6, COID7, COID8, COID9 | COID |

Table 24: True types of lung cancer

In Figure 7, we depict the gene expression map of the true types AD, NL, SQ and COID, indexed by 1, 2, 3 and 4 respectively.

By observing Figure 7, we have the following results:

1) The gene expression profiles of the true types are ranked by COID, NL, SQ and AD.

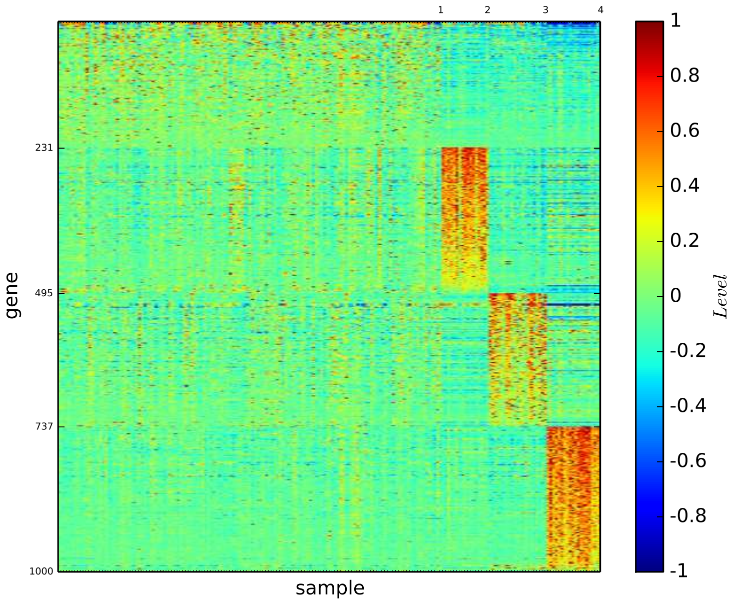


Figure 7: Gene map of true types of the lung cancer.

2) The true types AD, NL, SQ and COID are distinguished by the gene expression profiles.

3) For each type, most or almost all the genes in the corresponding type have high expression profiles for the type.

4) The type AD is not well-expressed by the genes in the first block $B_1$. This means that the type AD may not be a well-defined type.

## Similarity of the true types of lung cancer to the modules by the algorithms

In Table 25, we list the similarities of the true types of lung cancer identified by algorithms $\mathcal{E}^2$, $\mathcal{M}$ and $\mathcal{I}$.

| Similarity / Algorithm / Type | $\mathcal{E}^2$ | $\mathcal{M}$ | $\mathcal{I}$ | $\mathcal{E}^3$ |
|---|---|---|---|---|
| AD | 0.380 | 0.495 | 0.360 | 0.380 |
| NL | 0.890 | 0.882 | 0.868 | 0.890 |
| SQ | 0.865 | 0.865 | 0.837 | 0.751 |
| COID | 0.975 | 0.975 | 0.975 | 0.975 |
| Weighted average | 0.536 | 0.617 | 0.517 | 0.524 |

Table 25: Similarity of the cell types of lung Cancer identified by $\mathcal{E}^2$, $\mathcal{M}$ and $\mathcal{I}$, respectively.

## Modules identified by $\mathcal{M}$

Table 26 describes the modules of lung cancer identified by $\mathcal{M}$.
Figure 8 is the gene map of the modules of lung cancer identified by $\mathcal{M}$.

## Modules identified by $\mathcal{I}$

Table 27 describes the modules of lung cancer identified by $\mathcal{I}$.
Figure 9 is the gene map of the modules of lung cancer identified by $\mathcal{I}$.

## Modules of the lung cancer identified by $\mathcal{E}^2$

In Table 28, we list the classification of tumor types of the lung cancer identified by our algorithm $\mathcal{E}^2$.

By observing Table 28, we have the following:

(1) The types COID, NL and SQ are well-approximated.

(2) The type AD is basically divided into 11 modules.

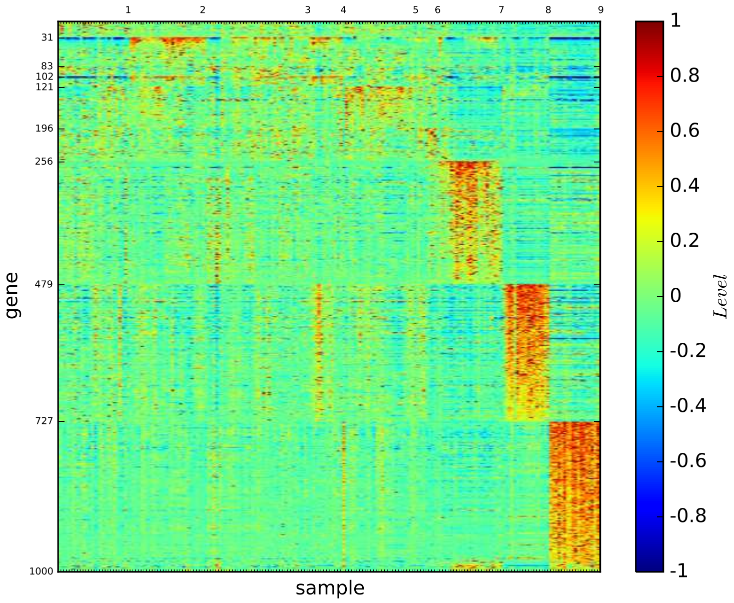


Figure 8: Gene map of the lung cancer identified by $\mathcal{M}$.

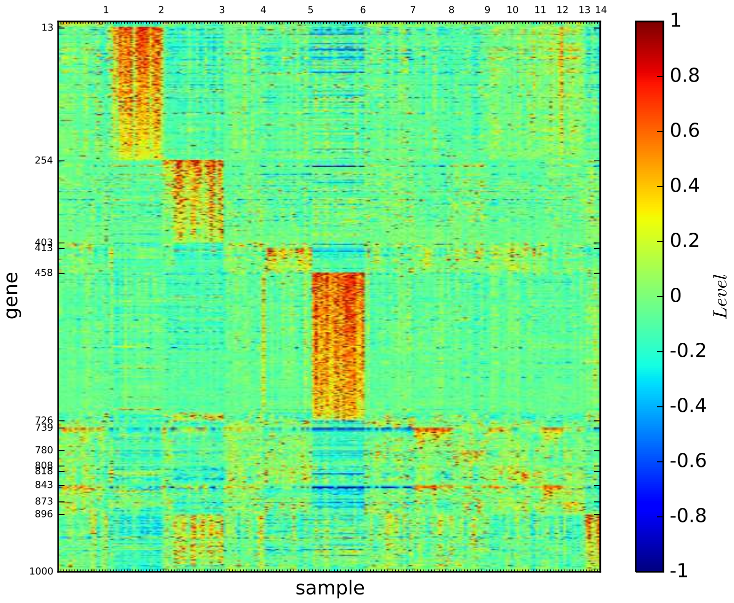


Figure 9: Gene map of the lung cancer identified by $\mathcal{I}$.

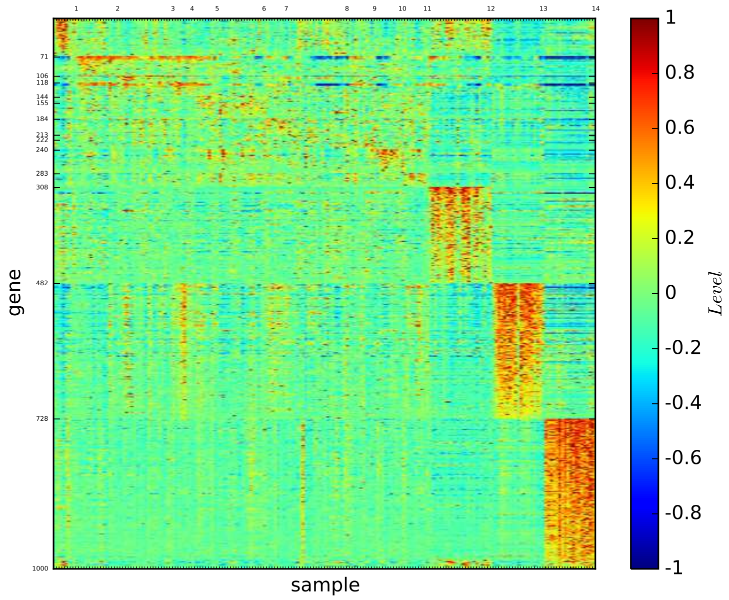


Figure 10: Gene map of the lung cancer identified by $\mathcal{E}^2$.

| Module | Samples | Cell type |
|---|---|---|
| 1 | AD043, AD157, AD164, AD221, AD236, AD360, AD31, AD287, AD314, AD5, AD320, AD7, AD296, AD4, AD212, AD340, AD355, AD167, AD309, AD294, AD163, AD169, NL504, AD331, AD186, AD201 | AD, NL |
| 2 | AD111, AD115, AD119, AD247, AD347, AD243, AD250, AD330, AD226, AD232, AD366, AD239, AD208, AD252, AD120, AD269, AD185, AD367, AD379, AD277, AD301, AD2, AD368, AD123, AD177, AD323, AD253 | AD |
| 3 | AD114, AD131, AD268, AD351, SQ6, SQ10, AD179, AD249, AD230, AD285, AD202, AD228, AD210, AD241, AD384, AD352, AD335, AD382, AD283, AD311, AD172, AD375, AD122, AD370, AD304, AD238, AD313, AD337, AD334, AD336, AD218, AD276, AD302, AD10, AD258, AD15, AD332, AD3 | AD |
| 4 | AD118, AD127, AD1, NL2378, AD259, AD18, AD346, AD305, AD266, AD159, AD207, AD162, COID8 | AD, COID, NL |
| 5 | AD130, AD158, AD173, AD178, AD318, AD356, AD255, AD225, AD315, AD308, AD260, AD240, AD203, AD338, AD213, AD353, AD188, AD224, AD183, AD362, AD170, AD327, AD361, AD19, AD350, AD317 | AD |
| 6 | AD136, AD261, AD262, AD16, AD187, AD374, AD383, AD299 | AD |
| 7 | AD234, AD275, AD295, AD341, SQ20, SQ4389, SQ14, SQ1670, SQ5, SQ13, SQ3624, SQ8, SQ2557, SQ3529, SQ3197, SQ2921, SQ4172, SQ1174, SQ7324, SQ2572, SQ6147, SQ4, SQ5897 | AD, SQ |
| 8 | AD267, AD363, NL1179, NL1698, NL268, NL6943, NL3681, N-L4353, NL6084, NL2562, NL6853, NL3104, NL279, NL4083, N-L1884, NL7530, NL1675 | AD, NL |
| 9 | COID1429, COID4518, COID14, COID16, COID7, COID5, COID11, COID6, COID3, COID4, COID3580, COID18, COID9, COID4385, COID13, COID12, COID10, COID9794, COID2260 | COID |

Table 26: The modules of lung cancer identified by $\mathcal{M}$

In Figure 10, we depict the gene expression map of the lung cancer by the classification identified by our algorithm $\mathcal{E}^2$.

By observing Figure 10 and Table 28, we have the following results:

(1) The modules, denoted by $X_1, \cdots, X_{14}$, are distinguishable by the gene expression map.

(2) Modules from $X_1$ to $X_{11}$ are a basically a classification of AD, in which $X_1$, $X_2$, $X_3$, $X_4$, $X_10$ and $X_{11}$ are well-expressed by the corresponding gene sets, and others are not well-defined.

(3) Modules $X_{12}$, $X_{13}$ and $X_{14}$ are almost equal to SQ, NL and COID, respectively. They are well-defined by the corresponding gene expression patterns.

In table 29 and 30, we describe the top $10$ genes of the modules of the lung cancer identified by $\mathcal{E}^2$.

## $3$-Dimensional gene expression map of the lung cancer

In tables 31 and 32, we describe the modules and submodules of cell samples of the lung cancer identified by our algorithm $\mathcal{E}^3$.

According to Tables 28, and 31 and 32, the classification of the algorithm $\mathcal{E}^3$ is basically a refined classification of that given by the algorithm $\mathcal{E}^2$ for the lung cancer cell samples.

In Figure 11, we depict the gene expression map of the refined classification of cell modules and submodules identified by our algorithm $\mathcal{E}^3$, with the ordering in Tables 31 and 32.

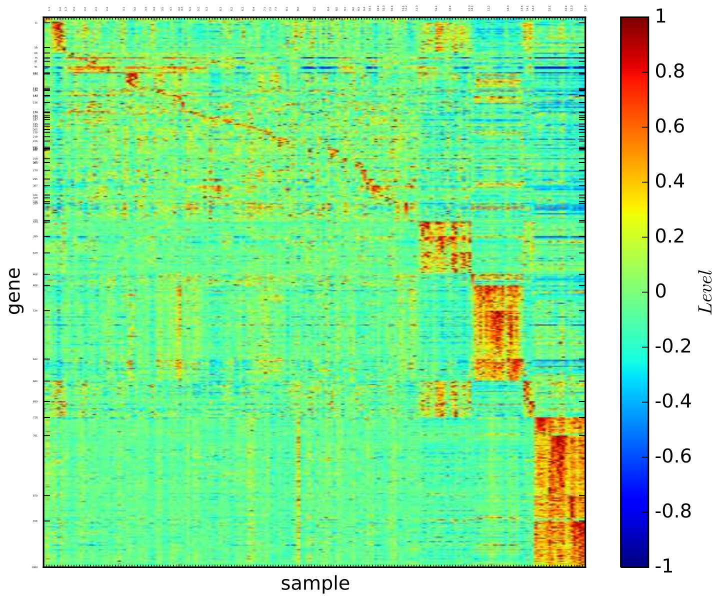


Figure 11: Three-dimensional gene map of lung cancer identified by $\mathcal{E}^3$.

According to Figure 11, we observed that almost all the submodules $Y$ of the lung cancer identified by $\mathcal{E}^3$ are well-defined by the corresponding gene expression patterns, due to the fact that, the high expression profiles coded by the red color are concentrated on the diagonal blocks of the gene map. This result is very interesting, because, for the AD samples, the gene expression files are not high for almost all the cell samples.

In tables 33, 34 and 35, we describe the top $10$ genes of the submodules of the lung cancer identified by $\mathcal{E}^3$.

| Module | Samples | Cell type |
|---|---|---|
| 1 | AD043, AD122, AD123, AD172, AD177, AD210, AD238, AD241, AD276, AD283, AD311, AD334, AD337, AD352, AD370, AD375, AD384, SQ5897 | AD, SQ |
| 2 | AD267, AD309, AD363, AD4, NL1179, NL1675, NL1698, N-L1884, NL2562, NL268, NL279, NL3104, NL3681, NL4083, NL4353, NL504, NL6084, NL6853, NL6943, NL7530 | AD, NL |
| 3 | AD234, AD275, AD295, AD341, SQ1174, SQ1670, SQ2557, SQ2572, SQ2921, SQ3197, SQ3529, SQ3624, SQ4172, SQ4389, SQ6147, SQ7324, SQ13, SQ14, SQ20, SQ4, SQ5, SQ8 | AD, SQ |
| 4 | AD114, AD159, AD162, AD179, AD202, AD207, AD228, AD266, AD285, AD299, AD305, AD313, AD336, AD382, COID8 | AD, COID |
| 5 | AD130, AD170, AD178, AD183, AD188, AD201, AD203, AD213, AD224, AD240, AD268, AD318, AD327, AD331, AD338, AD353, AD362 | AD |
| 6 | COID1429, COID2260, COID3580, COID4385, COID4518, COID9794, COID10, COID11, COID12, COID13, COID14, COID16, COID18, COID3, COID4, COID5, COID6, COID7, COID9 | COID |
| 7 | AD157, AD163, AD164, AD167, AD169, AD212, AD221, AD236, AD287, AD296, AD314, AD320, AD340, AD355, AD360, AD31, AD5, AD7 | AD |
| 8 | AD111, AD115, AD120, AD185, AD208, AD239, AD252, AD269, AD277, AD301, AD367, AD368, AD379, AD2 | AD |
| 9 | AD187, AD218, AD249, AD258, AD302, AD304, AD332, AD335, AD374, AD383, AD10, AD15, AD3 | AD |
| 10 | AD119, AD226, AD232, AD243, AD247, AD250, AD330, AD347, AD366 | AD |
| 11 | AD158, AD173, AD225, AD255, AD308, AD315, AD317, AD356, AD361, AD19 | AD |
| 12 | AD118, AD127, AD253, AD259, AD346, AD18, AD1, NL2378 | AD, NL |
| 13 | AD136, AD260, AD261, AD262, AD294, AD323, AD350, AD16 | AD |
| 14 | AD131, AD186, AD230, AD351, SQ10, SQ6 | AD, SQ |

Table 27: The modules of lung cancer identified by $\mathcal{I}$

| Module | Samples | Cell type |
|---|---|---|
| 1 | AD043, AD131, SQ10, SQ6, AD186, AD351, AD179, AD230, AD285 | AD (but SQ10, SQ6) |
| 2 | AD111, AD115, AD120, AD277, AD379, AD185, AD252, AD367, AD301, AD2, AD368, AD269, AD208, AD239, AD177 | AD |
| 3 | AD114, AD122, AD259, AD370, AD375, AD172, AD241, AD210, AD304, AD335, AD311, AD283, AD384, AD352, AD238, AD123, AD337, AD334, AD276, AD266 | AD |
| 4 | AD118, AD127, AD1, NL2378, AD18, AD346, AD253 | AD |
| 5 | AD119, AD226, AD243, AD232, AD250, AD366, AD347, AD247, AD330 | AD |
| 6 | AD130, AD170, AD183, AD224, AD249, AD332, AD3, AD15, AD362, AD188, AD213, AD338, AD240, AD353, AD268, AD158, AD203 | AD |
| 7 | AD136, AD261, AD262, AD350, AD260, AD294, AD16, AD323 | AD |
| 8 | AD157, AD167, AD313, AD336, AD382, COID8, AD374, AD212, AD164, AD355, AD296, AD31, AD236, AD360, AD287, AD221, AD314, AD5, AD320, AD7, AD340, AD309 | AD (but COID8) |
| 9 | AD159, AD207, AD162, AD228, AD305, AD202, AD187, AD299, AD302, AD383 | AD |
| 10 | AD163, AD169, AD178, AD331, AD201, AD318, AD327, AD10, AD258, AD218 | AD |
| 11 | AD173, AD225, AD255, AD308, AD317, AD356, AD19, AD361, AD315 | AD |
| 12 | AD234, SQ1174, SQ4172, SQ2557, SQ2921, SQ3197, SQ3529, SQ3624, SQ8, SQ13, AD275, SQ7324, SQ1670, SQ5, SQ14, AD295, SQ4389, SQ20, AD341, SQ2572, SQ5897, SQ6147, SQ4 | SQ (but AD234, AD295, AD341) |
| 13 | AD267, AD363, NL1179, NL1698, NL3104, NL6853, NL6943, NL3681, NL279, NL268, NL2562, NL4353, NL6084, NL4083, NL1884, NL7530, NL1675, NL504, AD4 | NL (but AD267, AD363, AD4) |
| 14 | COID1429, COID4518, COID14, COID7, COID11, COID5, COID6, COID3, COID3580, COID4, COID16, COID18, COID9, COID4385, COID13, COID12, COID2260, COID9794, COID10 | COID |

Table 28: Modules of cell samples of lung cancer identified by $\mathcal{E}^2$.

| Modules identified by $\mathcal{E}^2$ | Top 10 genes of the modules of lung cancer identified by $\mathcal{E}^2$ | Top 10 genes of true types | Type |
|---|---|---|---|
| 1 | 31545_at, 709_at, 39677_at, 33656_at, 34592_at, 39173_at, 38679_g_at, 149_at, 38728_at, 33266_at | 37864_s_at, 33273_f_at, 33274_f_at, 38194_s_at, 33500_i_at, 33501_r_at, 33499_s_at, 41827_f_at, 32626_at, 33956_at | AD |
| 2 | 33274_f_at, 41827_f_at, 33273_f_at, 41164_at, 38194_s_at, 41165_g_at | 37864_s_at, 33273_f_at, 33274_f_at, 38194_s_at, 33500_i_at, 33501_r_at, 33499_s_at, 41827_f_at, 32626_at, 33956_at | AD |
| 3 | 37864_s_at, 34342_s_at, 39945_at, 39167_r_at, 37345_at, 38069_at, 36028_at, 38566_at, 33143_s_at, 37422_at | 37864_s_at, 33273_f_at, 33274_f_at, 38194_s_at, 33500_i_at, 33501_r_at, 33499_s_at, 41827_f_at, 32626_at, 33956_at | AD |
| 4 | 34105_f_at, 31586_f_at, 35566_f_at, 33500_i_at, 33499_s_at, 33501_r_at, 31319_at, 37542_at, 40830_at, 506_s_at | 37864_s_at, 33273_f_at, 33274_f_at, 38194_s_at, 33500_i_at, 33501_r_at, 33499_s_at, 41827_f_at, 32626_at, 33956_at | AD |
| 5 | 36771_at, 1729_at, 37486_f_at, 34796_at, 34726_at, 39670_at, 38950_r_at, 634_at, 37844_at, 32116_at | 37864_s_at, 33273_f_at, 33274_f_at, 38194_s_at, 33500_i_at, 33501_r_at, 33499_s_at, 41827_f_at, 32626_at, 33956_at | AD |
| 6 | 341491_s_at, 38047_at, 38582_at, 33331_at, 32081_at, 1467_at, 36066_at, 32787_at, 35151_at, 34892_at | 37864_s_at, 33273_f_at, 33274_f_at, 38194_s_at, 33500_i_at, 33501_r_at, 33499_s_at, 41827_f_at, 32626_at, 33956_at | AD |
| 7 | 38378_at, 33956_at, 1375_s_at, 884_at, 36105_at, 41106_at, 31888_s_at, 39712_at, 37459_at, 33354_at | 37864_s_at, 33273_f_at, 33274_f_at, 38194_s_at, 33500_i_at, 33501_r_at, 33499_s_at, 41827_f_at, 32626_at, 33956_at | AD |

Table 29: Top 10 genes of the modules of lung cancer identified by $\mathcal{E}^2$ and the true types-1

| Modules identified by $\mathcal{E}^2$ | Top 10 genes of the modules of lung cancer identified by $\mathcal{E}^2$ | Top 10 genes of true types | Type |
|---|---|---|---|
| 8 | 40237_at, 32028_at, 33674_at, 35272_at, 39021_at, 40364_at, 35714_at, 36412_s_at, 31886_at | 37864_s_at, 33273_f_at, 33274_f_at, 38194_s_at, 33500_i_at, 33501_r_at, 33499_s_at, 41827_f_at, 32626_at, 33956_at | AD |
| 9 | 1953_at, 40128_at, 33933_at, 36100_at, 36101_s_at, 2035_s_at, 35276_at, 1582_at, 37037_at, 32134_at | 37864_s_at, 33273_f_at, 33274_f_at, 38194_s_at, 33500_i_at, 33501_r_at, 33499_s_at, 41827_f_at, 32626_at, 33956_at | AD |
| 10 | 700_s_at, 38783_at, 38784_g_at, 1083_s_at, 927_s_at, 33396_at, 34319_at, 35214_at, 829_s_at, 41153_f_at | 37864_s_at, 33273_f_at, 33274_f_at, 38194_s_at, 33500_i_at, 33501_r_at, 33499_s_at, 41827_f_at, 32626_at, 33956_at | AD |
| 11 | 37218_at, 41177_at, 41176_at, 34256_at, 393_s_at, 33754_at, 35842_at, 38261_at, 37754_at, 32715_at | 37864_s_at, 33273_f_at, 33274_f_at, 38194_s_at, 33500_i_at, 33501_r_at, 33499_s_at, 41827_f_at, 32626_at, 33956_at | AD |
| 12 | 613_at, 39070_at, 39016_r_at, 41266_at, 33322_i_at, 2027_at, 34301_r_at, 39015_f_at, 36785_at, 33323_r_at | 613_at, 41266_at, 39016_r_at, 39070_at, 33322_i_at, 36133_at, 36785_at, 37347_at, 39015_f_at, 33323_r_at | SQ |
| 13 | 40419_at, 35261_at, 1815_g_at, 35282_r_at, 40456_at, 38691_s_at, 36119_at, 37039_at, 40331_at, 41198_at | 40419_at, 1815_g_at, 35282_r_at, 36119_at, 40841_at, 35261_at, 32562_at, 38119_at, 38691_s_at, 39775_at | NL |
| 14 | 34849_at, 41107_at, 39823_at, 37588_s_at, 41675_at, 32254_at, 36192_at, 40275_at, 37210_at, 40986_s_at | 41107_at, 34849_at, 34847_s_at, 37588_s_at, 36894_at, 39823_at, 40275_at, 32254_at, 40986_s_at, 41675_at | COID |

Table 30: Top 10 genes of the modules of lung cancer identified by $\mathcal{E}^2$ and the true types-2

| Module | Submodule | Cell type |
| --- | --- | --- |
| 1 | AD043, AD351, AD179 | AD |
| | AD131, SQ10, SQ6, AD186 | AD, SQ |
| | AD230, AD285 | AD |
| 2 | AD111, AD368, AD177 | AD |
| | AD115, AD367, AD301, AD2 | AD |
| | AD120, AD277, AD379, AD185 | AD |
| | AD252, AD208, AD239, AD269 | AD |
| 3 | AD114, AD283, AD384, AD334, AD352, AD337 | AD |
| | AD122, AD259, AD370, AD375 | AD |
| | AD172, AD238, AD276, AD210 | AD |
| | AD241, AD335, AD304 | AD |
| | AD311, AD266, AD123 | AD |
| 4 | AD118, AD346, AD18 | AD |
| | AD127, AD1, NL2378 | AD, NL |
| | AD253 | AD |
| 5 | AD119, AD347, AD247 | AD |
| | AD226, AD243, AD232 | AD |
| | AD250, AD366, AD330 | AD |
| 6 | AD130, AD362, AD183, AD170, AD224 | AD |
| | AD249, AD332, AD3, AD15 | AD |
| | AD188, AD353,AD158, AD268 | AD |
| | AD213, AD338, AD203, AD240 | AD |
| 7 | AD136, AD294, AD261, AD262 | AD |
| | AD350, AD260 | AD |
| | AD16, AD323 | AD |
| 8 | AD157, AD212, AD167, AD374 | AD |
| | AD313, AD336, AD382, COID8 | AD, COID |
| | AD164, AD236, AD221, AD5, AD287, AD314 | AD |
| | AD355, AD296, AD31, AD360, AD309 | AD |
| | AD320, AD7, AD340 | AD |

Table 31: Modules and submodules of cell samples of lung cancer identified by $\mathcal{E}^3$-1.

| Module | Submodule | Cell type |
|---|---|---|
| 9 | AD159, AD207, AD162 | AD |
| | AD228, AD305, AD202 | AD |
| | AD187, AD299 | AD |
| | AD302, AD383 | AD |
| 10 | AD163, AD169 | AD |
| | AD178, AD331, AD318 | AD |
| | AD201, AD258 | AD |
| | AD327, AD10, AD218 | AD |
| 11 | AD173, AD255, AD315, AD225 | AD |
| | AD308 | AD |
| | AD317, AD356, AD19, AD361 | AD |
| 12 | AD234, SQ1174, SQ4172, SQ2557, SQ2921, SQ3197, SQ3529 | SQ(but AD234) |
| | SQ3624, SQ8, SQ13,AD275, SQ7324 | SQ(but AD275) |
| | SQ1670, SQ14, AD295, SQ4389, SQ20, AD341, SQ5 | SQ, AD |
| 13 | AD267 | AD |
| | AD363, NL1675, NL4353, NL7530, NL1179, NL6084 | NL(but AD363) |
| | NL1698, NL3104, NL6853,NL6943, NL3681, NL268, NL279 | NL |
| | NL2562, NL1884, NL4083, NL504, AD4 | NL(but AD4) |
| 14 | SQ2572, SQ5897 | SQ |
| | SQ6147, SQ4 | SQ |
| 15 | COID1429, COID4518, COID14, COID7, COID11, COID16 | COID |
| | COID5, COID4385, COID13, COID2260, COID9794, COID10 | COID |
| | COID6, COID12 | COID |
| | COID3, COID3580, COID4, COID9, COID18 | COID |

Table 32: Modules and submodules of cell samples of lung cancer identified by $\mathcal{E}^3$-2.

| Submodule | Top 10 genes of the submodules of lung cancer identified by $\mathcal{E}^3$ |
|---|---|
| 1.1 | 1150_at, 41152_f_at, 31950_at, 37561_at, 35442_at, 40581_at, 35444_at, 38640_at, 1585_at, 2089_s_at |
| 1.2 | 38679_g_at, 709_at, 35312_at, 31545_at, 151_s_at, 37347_at, 40117_at, 32590_at, 36153_at, 38992_at |
| 1.3 | 34592_at, 35916_s_at, 223_at, 41278_at, 32151_at, 894_g_at, 1235_at, 33883_at, 39515_s_at, 32232_at |
| 2.1 | 37978_at, 35807_at, 32548_at, 40311_at, 35698_at, 38388_at, 35822_at, 40425_at, 31610_at |
| 2.2 | 33274_f_at, 33273_f_at, 442_at, 41403_at, 33714_at, 32081_at |
| 2.3 | 41294_at, 40079_at, 38276_at, 38582_at, 38131_at, 34970_r_at, 37625_at, 36676_at, 33808_at, 38129_at |
| 2.4 | 34105_f_at, 41827_f_at, 35566_f_at, 33499_s_at, 31319_at, 41165_g_at, 41064_at, 33304_at, 38578_at, 37467_at |
| 3.1 | 37864_s_at, 40830_at |
| 3.2 | 35146_at, 41191_at, 39167_r_at, 38775_at, 32847_at, 39945_at, 33891_at, 38566_at, 1597_at, 37345_at |
| 3.3 | 35350_at, 33070_at |
| 3.4 | 31888_s_at, 38708_at |
| 3.5 | 34342_s_at, 33956_at, 34091_s_at, 39134_at, 512_at, 38069_at, 33133_at, 40585_at, 38506_at |
| 4.1 | 38194_s_at |
| 4.2 | 31586_f_at, 33501_r_at, 39081_at, 506_s_at, 33862_at, 38786_at, 37630_at, 40766_at, 31698_at, 37394_at |
| 4.3 | 38404_at, 34892_at, 40074_at, 32028_at, 39008_at, 40960_at, 35214_at, 1450_g_at, 37032_at, 36028_at |
| 5.1 | 41164_at |
| 5.2 | 33500_i_at, 40941_at, 39670_at, 37844_at, 38966_at, 32551_at |
| 5.3 | 34726_at, 660_at, 39260_at |
| 6.1 | 32787_at, 36066_at, 33052_at, 634_at |
| 6.2 | 36972_at, 32626_at, 33213_g_at, 35805_at, 36666_at, 35151_at, 36615_at, 39113_at |
| 6.3 | 34256_at, 33674_at, 41491_s_at, 40762_g_at |
| 6.4 | 38064_at, 31598_s_at, 38047_at, 32870_g_at, 32034_at, 130_s_at |
| 7.1 | 38378_at, 37805_at, 39254_at, 1427_g_at, 677_s_at |
| 7.2 | 36105_at, 33774_at, 1368_at, 38379_at, 999_at, 37486_f_at, 37779_at, 41585_at |
| 7.3 | 884_at, 36100_at, 1953_at, 885_g_at, 33354_at, 1375_s_at, 943_at, 36036_at |

Table 33: Top 10 genes of the submodules of lung cancer identified by $\mathcal{E}^3$-1

| Submodule | Top 10 genes of the submodules of lung cancer identified by $\mathcal{E}^3$ |
|---|---|
| 8.1 | 40237_at, 577_at, 38124_at, 493_at, 33143_s_at, 38789_at, 37351_at, 32116_at, 35714_at, 33121_g_at |
| 8.2 | 36412_s_at, 37912_at |
| 8.3 | 31886_at |
| 8.4 | 38095_i_at, 1825_at |
| 8.5 | 34255_at, 33904_at, 39339_at, 40445_at, 35276_at, 37228_at, 1317_at, 39721_at, 31431_at, 35272_at |
| 9.1 | 34760_at, 41256_at, 36101_s_at, 41188_at, 36933_at, 34235_at, 40813_at |
| 9.2 | |
| 9.3 | 1582_at, 40364_at, 37640_at, 39432_at, 32134_at, 891_at, 1794_at, 33933_at, 40128_at, 1467_at |
| 9.4 | 40712_at, 32749_s_at, 34678_at, 2035_s_at, 359_at, 37842_at, 40504_at, 33305_at, 36336_s_at, 37103_at |
| 10.1 | 35666_at, 41153_f_at, 32980_f_at, 41156_g_at, 39714_at, 37037_at, 33799_at, 39351_at, 41155_at, 32527_at |
| 10.2 | 38783_at, 700_s_at, 38784_g_at, 927_s_at, 33383_f_at, 33396_at, 1083_s_at, 829_s_at, 239_at, 38066_at |
| 10.3 | 38750_at, 691_g_at, 35766_at, 33212_at, 34409_at |
| 10.4 | 34319_at, 33218_at, 38998_g_at, 39087_at, 1388_g_at, 38469_at, 40506_s_at |
| 11.1 | 41176_at, 41177_at, 34230_r_at |
| 11.2 | 38785_at, 32051_at, 37754_at, 36773_f_at, 195_s_at, 38261_at, 37039_at, 33894_at, 393_s_at, 38833_at |
| 11.3 | 31775_at, 33754_at, 1890_at |
| 12.1 | 613_at, 39016_r_at, 41266_at, 39015_f_at, 36133_at, 40879_at, 38608_at, 31791_at, 32123_at, 39795_at |
| 12.2 | 757_at, 769_s_at, 31444_s_at, 33322_i_at, 2027_at, 33323_r_at, 36785_at, 34301_r_at, 33929_at, 38840_s_at |
| 12.3 | 36883_at, 40365_at, 863_g_at, 39416_at, 39052_at, 35726_at, 35756_at, 862_at, 1898_at, 2047_s_at |

Table 34: Top 10 genes of the submodules of lung cancer identified by $\mathcal{E}^3$-2

| Submodule | Top 10 genes of the submodules of lung cancer identified by $\mathcal{E}^3$ |
|---|---|
| 13.1 | 40456_at, 36889_at, 37168_at, 36915_at, 36708_at, 37218_at, 33261_at, 1715_at, 32715_at, 38796_at |
| 13.2 | 1815_g_at, 40419_at, 41198_at, 35261_at, 37976_at, 39631_at, 39760_at, 38239_at, 38691_s_at, 37022_at |
| 13.3 | 32052_at, 37247_at, 34224_at, 1135_at, 36119_at, 32562_at, 38454_g_at, 31525_s_at, 40994_at, 35868_at |
| 13.4 | 40331_at, 925_at, 39728_at, 38685_at, 37684_at, 33283_at, 32811_at, 39775_at, 38406_f_at, 38110_at |
| 14.1 | 37333_at, 39070_at, 38689_at, 34003_at, 318_at, 37717_at, 32825_at, 319_g_at, 945_at, 37940_f_at |
| 14.2 | 39173_at, 32843_s_at, 40304_at, 1884_s_at, 38728_at, 38075_at, 39350_at, 41295_at, 33109_f_at, 33108_i_at |
| 15.1 | 33329_at, 41120_at, 39823_at, 40808_at, 37529_at, 38313_at, 916_at, 197_at, 41435_at, 39430_at |
| 15.2 | 39117_at, 39864_at, 39666_at, 37478_at, 36894_at, 40272_at, 37579_at, 39561_at, 40986_s_at, 37210_at |
| 15.3 | 38279_at, 36867_at, 37904_s_at, 32223_at, 39026_r_at, 35264_at, 32731_at, 41289_at, 41337_at, 40825_at |
| 15.4 | 32138_at, 40275_at, 35767_at, 34849_at, 37274_at, 185_at, 39427_at, 36192_at, 32836_at, 34773_at |

Table 35: Top 10 genes of the submodules of lung cancer identified by $\mathcal{E}^3$-3

# Normal Tissues

## True types

Table 36 describes the true types of the normal tissues.

In Figure 12, we depict the gene expression map of the 13 cell types of normal tissues, indexed as the same as that in Table 36, that is, the ordering of the true types in the figure is: Breast, Prostate, Lung, Colon, Germinal, Bladder, Uterus, Peripheral, Kidney, Pancreas, Ovary, Whole, and Cerebellum.

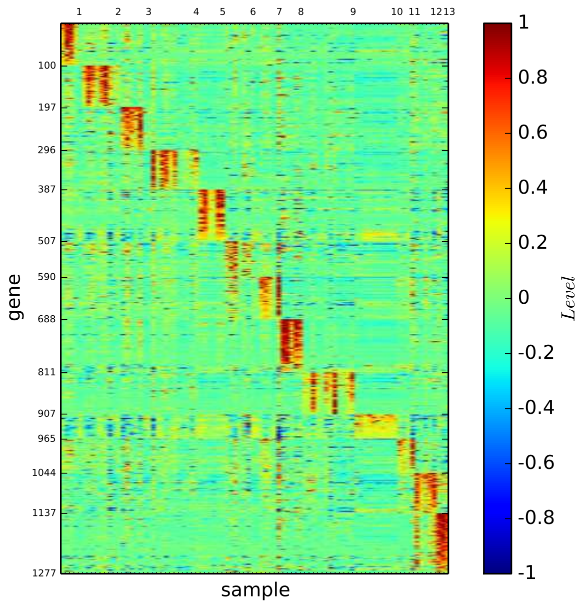


Figure 12: Gene map of true types of the normal tissues.

According to Figure 12, we observe the following results:

1) All the types are distinguishable by the gene expression map.
2) Bladder, Pancreas and Ovary are not well-expressed, and all others are highly expressed.

## Similarity

In Table 37, we give the similarities of the true types of the normal tissues identified by the algorithms $\mathcal{E}^2$, $\mathcal{M}$ and $\mathcal{I}$.

## Gene map of the normal tissues by $\mathcal{M}$

Table 38 describes the gene map of the modules of the normal tissues identified by $\mathcal{M}$.

Figure 13 is the gene map of the modules of the normal tissues identified by $\mathcal{M}$.

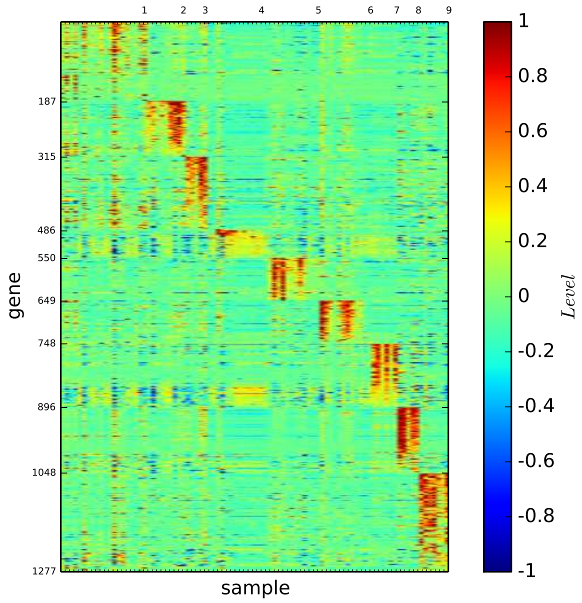


Figure 13: Gene map of the normal tissues identified by $\mathcal{M}$.

## Gene map of the modules of the normal tissues identified by $\mathcal{I}$

Table 39 describes the gene map of the modules of the normal tissues identified by $\mathcal{I}$.

Figure 14 is the gene map of the modules of the normal tissues identified by $\mathcal{I}$.

## Gene map of the modules of the normal tissues identified by $\mathcal{E}^2$

In Table 40, we give the classification of cell types of the normal tissues identified by our algorithm $\mathcal{E}^2$.

In Figure 15, we depict the gene expression map of the classification of normal tissues given by our algorithm $\mathcal{E}^2$.

By observing Table 40 and Figure 15, we have the following results:

(1) Modules 1, 2, 7 and 11 are exactly the Breast, Prostate, Germinal and Peripheral, respectively.

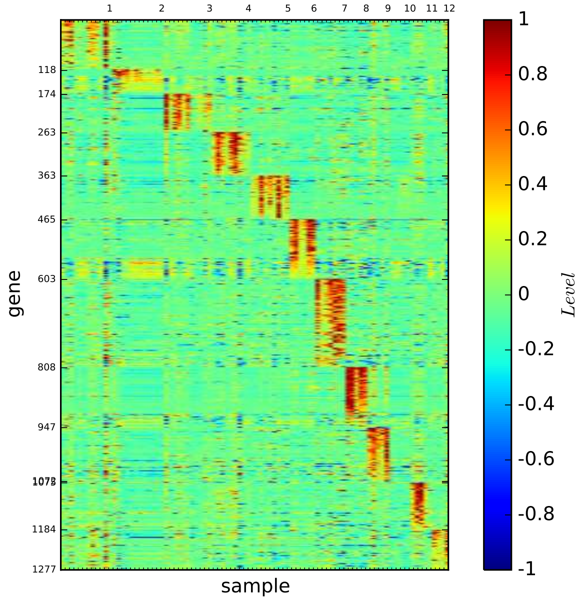


Figure 14: Gene map of the normal tissues identified by $\mathcal{I}$.

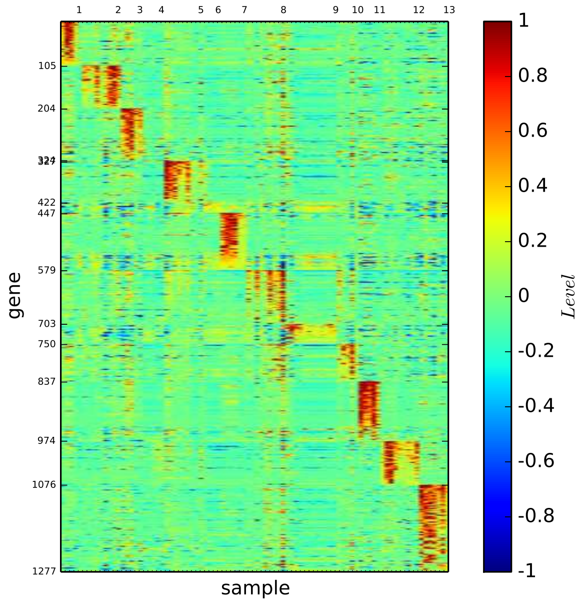


Figure 15: Gene map of the normal tissues identified by $\mathcal{E}^2$.

(2) Modules 3, 5, 9, 10 and 12 are essentially the types Lung, Colon, Pancreas, Ovary, and Kidney, which are highly expressed by the gene expression map.

(3) Module 4 consists of a few lung and kidney cells. This module is not well-expressed by the gene expression map.

(4) Module 6 is a combination of some colon and uterus cells, which is not well-expressed by the gene map.

(5) Module 8 is a combination of Bladder, Uterus, and Whole, which is highly expressed by the gene expression map.

(6) Module 13 is a combination of Whole and Cerebellum, which is highly expressed by the gene map.

In Tables 41, 42 and 43, we describe the top $10$ genes of the modules of the normal tissues identified by $\mathcal{E}^2$.

## $3$-Dimensional gene expression map of the normal tissues

In Table 44, we describe the modules and submodules of cell samples of the normal tissues identified by our algorithm $\mathcal{E}^3$.

In Figure 16, we depict the gene expression map of the refined classification of cell modules and submodules of the normal tissues identified by our algorithm $\mathcal{E}^3$ with ordering given in Table 44.

By observing Tables 40 and 44, and Figures 15 and 16, we have the following results:

(1) Modules 1, 2, 3, 5, 7, 8, 9, 10, 11, 12 are essentially the Breast, Prostate, Lung, Colon, Germinal, Bladder, Pancreas, Ovary, Peripheral, Kidney types, respectively.

(2) Module 4 is a combination of a few Lung and Kidney,

(3) Module 6 is the combination of a two Colon and Uterus,

(4) Module 13 is Cerebellum with three Whole cells.

(5) In Modules 4, 6, and 13, the submodules distinguish correctly the exact type of the cells.

(6) In all the cases, the submodules of a module are distinguishable.

(7) All the modules are distinguishable.

(8) Almost all the submodules are highly expressed by the gene expression map.

In Tables 45, 46 and 47, we describe the top $10$ genes of the submodules of the normal tissues identified by $\mathcal{E}^3$.

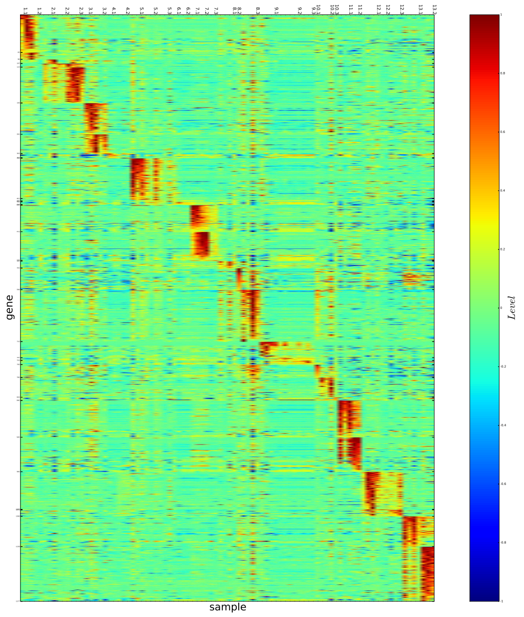


Figure 16: Three-dimensional gene map of normal tissues identified by $\mathcal{E}^3$.

| True type | Samples | Cell type |
|---|---|---|
| 1 | Normal__Breast_BR_1, Normal__Breast_BR_2, Normal__Breast_BR_3, Normal__Breast_BR_4, Normal__Breast_93_I_184 | Breast |
| 2 | Normal__Prostate_PR_2, Normal__Prostate_PR_3, Normal__Prostate_PR_4, Normal__Prostate_PR_5, Normal__Prostate_PR_6, Normal__Prostate_PR_7, Normal__Prostate_NLP1N, Normal__Prostate_NLP10N, Normal__Prostate_95_I_255 | Prostate |
| 3 | Normal__Lung_LU_1, Normal__Lung_LU_2, Normal__Lung_LU_3, Normal__Lung_LU_6, Normal__Lung_LU_7, Normal__Lung_93_I_028_(I) , Normal__Lung_HCTN_LUN2_(18763_A2A) | Lung |
| 4 | Normal__Colon_CR_1, Normal__Colon_CR_2, Normal__Colon_CR_3, Normal__Colon_CR_4, Normal__Colon_CR_5, Normal__Colon_CR_6, Normal__Colon_CR_7, Normal__Colon_CR_8, Normal__Colon_CR_9, Normal__Colon_CR_10, Normal__Colon_9912c071_CC | Colon |
| 5 | Normal__Germinal_Center_GC2, Normal__Germinal_Center_GC3, Normal__Germinal_Center_GC38, Normal__Germinal_Center_GC44, Normal__Germinal_Center_GC45, Normal__Germinal_Center_GC48 | Germinal |
| 6 | Normal__Bladder_BL_1, Normal__Bladder_BL_2, Normal__Bladder_BL_3, Normal__Bladder_BL_4, Normal__Bladder_BL_5, Normal__Bladder_BL_6, Normal__Bladder_95_I_285 | Bladder |
| 7 | Normal__Uterus_UT_1, Normal__Uterus_UT_2, Normal__Uterus_UT_3, Normal__Uterus_UT_4, Normal__Uterus_UT_5, Normal__Uterus_UT_8 | Uterus |
| 8 | Normal__Peripheral_Blood_Poly_POLY1, Normal__ Peripheral_Blood_Poly_POLY2, Normal__Peripheral_ Blood_Poly_POLY3, Normal__Peripheral_Blood_Mono_MONO5, Normal__Peripheral_Blood_Mono_MONO6 | Peripheral |
| 9 | Normal__Kidney_KT_1, Normal__Kidney_KT_2, Normal__Kidney_KT_3, Normal__Kidney_KT_4, Normal__Kidney_KT_5, Normal__Kidney_KT_6, Normal__Kidney_Norm_622NO, Normal__Kidney_Norm_627, Normal__Kidney_Norm_613NPS, Normal__Kidney_Norm_613NS, Normal__Kidney_Norm_613NO, Normal__Kidney_Norm_627NS | Kidney |
| 10 | Normal__Pancreas_PAN_1, Normal__Pancreas_PAN_2, Normal__Pancreas_Pan_8N, Normal__Pancreas_Pan_11N, Normal__Pancreas_Pan13N, Normal__Pancreas_Pan_14N, Normal__Pancreas_Pan_43N, Normal__Pancreas_Pan_42N, Normal__Pancreas_Pan_40N, Normal__Pancreas_Pan_41N | Pancreas |
| 11 | Normal__Ovary_OV_1, Normal__Ovary_OV_2, Normal__Ovary_OV_3, Normal__Ovary_OV_4 | Ovary |
| 12 | Normal__Whole_Brain_BRAIN_1, Normal__Whole_Brain_BRAIN_2, Normal__Whole_Brain_BRAIN_3, Normal__Whole_Brain_BRAIN_4, Normal__Whole_Brain_BRAIN_5 | Whole |
| 13 | Normal__Cerebellum_Ncer_NCB1, Normal__Cerebellum_Brain_Ncer_S-51, Normal__Cerebellum_Brain_Ncer_S-125 | Cerebellum |

Table 36: True types of normal tissues

| Similarity \ Algorithm / Type | $\mathcal{E}^2$ | $\mathcal{M}$ | $\mathcal{I}$ | $\mathcal{E}^3$ |
|---|---|---|---|---|
| Breast | 1.0 | 0.5 | 1.0 | 1.0 |
| Prostate | 1.0 | 1.0 | 1.0 | 1.0 |
| Lung | 0.845 | 0.845 | 0.845 | 0.845 |
| Colon | 0.905 | 0.957 | 1.0 | 0.905 |
| Germinal | 1.0 | 1.0 | 1.0 | 1.0 |
| Bladder | 0.630 | 0.423 | 0.546 | 0.630 |
| Uterus | 0.480 | 0.456 | 0.707 | 0.408 |
| Peripheral | 1.0 | 1.0 | 1.0 | 1.0 |
| Kidney | 0.866 | 0.961 | 0.866 | 0.866 |
| Pancreas | 0.913 | 0.877 | 0.913 | 0.913 |
| Ovary | 0.894 | 0.447 | 1.0 | 0.894 |
| Whole | 0.676 | 0.676 | 0.676 | 0.676 |
| Cerebellum | 0.655 | 0.655 | 0.655 | 0.655 |
| Weighted average | 0.851 | 0.801 | 0.876 | 0.846 |

Table 37: Similarity of the types of normal tissues identified by $\mathcal{E}^2$, $\mathcal{M}$ and $\mathcal{I}$, respectively.

| Module | Samples | Cell type |
| --- | --- | --- |
| 1 | Normal__Breast_BR_1, Normal__Breast_BR_2, Normal__Breast_BR_4, Normal__Breast_BR_3, Normal__Breast_93_I_184, Normal__Ovary_OV_4, Normal__Ovary_OV_2, Normal__Ovary_OV_3, Normal__Ovary_OV_1, Normal__Uterus_UT_4, Normal__Uterus_UT_1, Normal__Uterus_UT_2, Normal__Uterus_UT_8, Normal__Uterus_UT_3, Normal__Whole_Brain_BRAIN_3, Normal__Bladder_BL_1, Normal__Bladder_BL_4, Normal__Bladder_95_I_285, Normal__Bladder_BL_2, Normal__Bladder_BL_3 | Breast, Ovary, Uterus, Whole, Bladder |
| 2 | Normal__Prostate_PR_2, Normal__Prostate_NLP1N, Normal__Prostate_NLP10N, Normal__Prostate_PR_5, Normal__Prostate_95_I_255, Normal__Prostate_PR_6, Normal__Prostate_PR_3, Normal__Prostate_PR_7, Normal__Prostate_PR_4 | Prostate |
| 3 | Normal__Lung_LU_1, Normal__Lung_LU_3, Normal__Lung_LU_6, Normal__Lung_LU_7, Normal__Lung_LU_2 | Lung |
| 4 | Normal__Lung_93_I_028_(I), Normal__Pancreas_Pan_41N, Normal__Bladder_BL_5, Normal__Bladder_BL_6, Normal__Pancreas_PAN_1, Normal__Pancreas_PAN_2, Normal__Pancreas_Pan_11N, Normal__Pancreas_Pan_14N, Normal__Pancreas_Pan_40N, Normal__Pancreas_Pan_8N, Normal__Pancreas_Pan13N, Normal__Pancreas_Pan_43N, Normal__Pancreas_Pan_42N | Lung, Pancreas, Bladder |
| 5 | Normal__Lung_HCTN_LUN2_(18763_A2A), Normal__Kidney_KT_1, Normal__Kidney_KT_3, Normal__Kidney_KT_6, Normal__Kidney_Norm_627, Normal__Kidney_KT_4, Normal__Kidney_KT_2, Normal__Kidney_Norm_613NO, Normal__Kidney_Norm_627NS, Normal__Kidney_Norm_622NO, Normal__Kidney_KT_5, Normal__Kidney_Norm_613NS, Normal__Kidney_Norm_613NPS | Lung, Kidney |
| 6 | Normal__Colon_CR_1, Normal__Colon_CR_3, Normal__Colon_CR_5, Normal__Colon_CR_8, Normal__Colon_CR_10, Normal__Colon_CR_6, Normal__Colon_CR_4, Normal__Colon_9912c071_CC, Normal__Colon_CR_9, Normal__Colon_CR_2, Normal__Colon_CR_7, Normal__Uterus_UT_5 | Colon, Uterus |
| 7 | Normal__Germinal_Center_GC2, Normal__Germinal_Center_GC3, Normal__Germinal_Center_GC38, Normal__Germinal_Center_GC45, Normal__Germinal_Center_GC44, Normal__Germinal_Center_GC48 | Germinal |
| 8 | Normal__Peripheral_Blood_Poly_POLY1, Normal__Peripheral_Blood_Poly_POLY2, Normal__Peripheral_Blood_Poly_POLY3, Normal__Peripheral_Blood_Mono_MONO5, Normal__Peripheral_Blood_Mono_MONO6 | Peripheral |
| 9 | Normal__Whole_Brain_BRAIN_1, Normal__Cerebellum_Ncer_NCB1, Normal__Cerebellum_Brain_Ncer_S-125, Normal__Cerebellum_Brain_Ncer_S-51, Normal__Whole_Brain_BRAIN_4, Normal__Whole_Brain_BRAIN_2, Normal__Whole_Brain_BRAIN_5 | Whole, Cerebellum |

Table 38: The modules of the normal tissues identified by $\mathcal{M}$

| Module | Samples | Cell type |
|---|---|---|
| 1 | Normal__Bladder_BL_1, Normal__Bladder_BL_2, Normal__Bladder_BL_3, Normal__Bladder_BL_4, Normal__Bladder_95_I_285, Normal__Uterus_UT_1, Normal__Uterus_UT_2, Normal__Uterus_UT_3, Normal__Uterus_UT_4, Normal__Uterus_UT_5, Normal__Uterus_UT_8, Normal__Whole_Brain_BRAIN_3 | Bladder, Uterus, Whole |
| 2 | Normal__Bladder_BL_5, Normal__Bladder_BL_6, Normal__Pancreas_PAN_1, Normal__Pancreas_PAN_2, Normal__Pancreas_Pan_8N, Normal__Pancreas_Pan_11N, Normal__Pancreas_Pan13N, Normal__Pancreas_Pan_14N, Normal__Pancreas_Pan_43N, Normal__Pancreas_Pan_42N, Normal__Pancreas_Pan_40N, Normal__Pancreas_Pan_41N | Bladder, Pancreas |
| 3 | Normal__Colon_CR_1, Normal__Colon_CR_2, Normal__Colon_CR_3, Normal__Colon_CR_4, Normal__Colon_CR_5, Normal__Colon_CR_6, Normal__Colon_CR_7, Normal__Colon_CR_8, Normal__Colon_CR_9, Normal__Colon_CR_10, Normal__Colon_9912c071_CC | Colon |
| 4 | Normal__Prostate_PR_2, Normal__Prostate_PR_3, Normal__Prostate_PR_4, Normal__Prostate_PR_5, Normal__Prostate_PR_6, Normal__Prostate_PR_7, Normal__Prostate_NLP1N, Normal__Prostate_NLP10N, Normal__Prostate_95_I_255 | Prostate |
| 5 | Normal__Kidney_KT_1, Normal__Kidney_KT_2, Normal__Kidney_KT_3, Normal__Kidney_KT_4, Normal__Kidney_KT_6, Normal__Kidney_Norm_622NO, Normal__Kidney_Norm_627, Normal__Kidney_Norm_613NO, Normal__Kidney_Norm_627NS | Kidney |
| 6 | Normal__Germinal_Center_GC2, Normal__Germinal_Center_GC3, Normal__Germinal_Center_GC38, Normal__Germinal_Center_GC44, Normal__Germinal_Center_GC45, Normal__Germinal_Center_GC48 | Germinal |
| 7 | Normal__Whole_Brain_BRAIN_1, Normal__Whole_Brain_BRAIN_2, Normal__Whole_Brain_BRAIN_4, Normal__Whole_Brain_BRAIN_5, Normal__Cerebellum_Ncer_NCB1, Normal__Cerebellum_Brain_Ncer_S-51, Normal__Cerebellum_Brain_Ncer_S-125 | Whole, Cerebellum |
| 8 | Normal__Peripheral_Blood_Poly_POLY1, Normal__Peripheral_Blood_Poly_POLY2, Normal__Peripheral_Blood_Poly_ POLY3, Normal__Peripheral_Blood_Mono_MONO5, Normal__Peripheral_Blood_Mono_MONO6 | Peripheral |
| 9 | Normal__Lung_LU_1, Normal__Lung_LU_2, Normal__Lung_LU_3, Normal__Lung_LU_6, Normal__Lung_LU_7 | Lung |
| 10 | Normal__Lung_93_I_028_(I), Normal__Lung_HCTN_LUN2_(18763_A2A), Normal__Kidney_KT_5, Normal__Kidney_Norm_613NPS, Normal__Kidney_Norm_613NS | Lung, Kidney |
| 11 | Normal__Breast_BR_1, Normal__Breast_BR_2, Normal__Breast_BR_3, Normal__Breast_BR_4, Normal__Breast_93_I_184 | Breast |
| 12 | Normal__Ovary_OV_1, Normal__Ovary_OV_2, Normal__Ovary_OV_3, Normal__Ovary_OV_4 | Ovary |

Table 39: The modules of the normal tissues identified by $\mathcal{I}$

| Module | Samples | Cell type |
| --- | --- | --- |
| 1 | Breast_BR_1, Breast_BR_2, Breast_BR_3, Breast_BR_4, Breast_93_I_184 | Breast |
| 2 | Prostate_PR_2, Prostate_PR_5, Prostate_95_I_255, Prostate_PR_6, Prostate_NLP10N, Prostate_NLP1N, Prostate_PR_3, Prostate_PR_7, Prostate_PR_4 | Prostate |
| 3 | Lung_LU_1, Lung_LU_2, Lung_LU_7, Lung_LU_6, Lung_LU_3 | Lung (missing Lung_93_I_028_(I) Lung_HCTN_LUN2_(18763_A2A)) |
| 4 | Lung_93_I_028_(I), Lung_HCTN_LUN2_ (18763_A2A), Kidney_KT_5, Kidney_Norm_613NPS, Kidney_Norm_613NS | Lung and kidney |
| 5 | Colon_CR_1, Colon_CR_4, Colon_9912c071_CC, Colon_CR_5, Colon_CR_6, Colon_CR_9, Colon_CR_8, Colon_CR_10, Colon_CR_3, | Colon (missing Colon_CR_2, Colon_CR_7) |
| 6 | Colon_CR_2, Colon_CR_7, Uterus_UT_1, Uterus_UT_5 | Colon, Uterus |
| 7 | Germinal_Center_GC2, Germinal_Center_GC3, Germinal_Center_GC45, Germinal_Center_GC48, Germinal_Center_GC44, Germinal_Center_GC38 | Germinal |
| 8 | Bladder_BL_1, Bladder_BL_4, Bladder_BL_2, Bladder_95_I_285, Whole_Brain_BRAIN_3, Bladder_BL_3, Uterus_UT_2, Uterus_UT_3, Uterus_UT_8 | Bladder, Uterus, Whole |
| 9 | Bladder_BL_5, Bladder_BL_6, Pancreas_PAN_1, Pancreas_PAN_2, Pancreas_Pan_8N, Pancreas_Pan_14N, Pancreas_Pan_43N, Pancreas_Pan_42N, Pancreas_Pan_41N, Pancreas_Pan_40N, Pancreas_Pan_11N, Pancreas_Pan13N | Pancreas (but Bladder_BL_5, Bladder_BL_6) |
| 10 | Uterus_UT_4, Ovary_OV_1, Ovary_OV_2, Ovary_OV_4, Ovary_OV_3 | Ovary (but Uterus_UT_4) |
| 11 | Peripheral_Blood_Poly_POLY1, Peripheral_Blood _Mono_MONO5, Peripheral_Blood_Mono_MONO6, Peripheral_Blood_Poly_POLY2, Peripheral_Blood _Poly_POLY3 | Peripheral |
| 12 | Kidney_KT_1, Kidney_Norm_622NO, Kidney_Norm_627, Kidney_KT_6, Kidney_KT_4, Kidney_KT_2, Kidney_KT_3, Kidney_Norm_613NO, Kidney_Norm_627NS | Kidney (missing Kidney_KT_5, Kidney_Norm_613NPS, Kidney _Norm_613NS) |
| 13 | Whole_Brain_BRAIN_1, Cerebellum_Ncer_NCB1, Cerebellum_Brain_Ncer_S-51, Cerebellum_Brain_Ncer_S-125, Whole_Brain_BRAIN_4, Whole_Brain_BRAIN_5, Whole_Brain_BRAIN_2 | Whole, Cerebellum |

Table 40: Modules of normal tissues identified by $\mathcal{E}^2$.

| Modules identified by $\mathcal{E}^2$ | Top 10 genes, normal tissues identified by $\mathcal{E}^2$ | Top 10 genes of true types | Type |
|---|---|---|---|
| 1 | Z28407_at, L00058_at, U41060_at, X07696_at, J00124_at, J02611_at, C16161_s_at, U85658_at, HG2465-HT4871_at, X84707_rna1_at | Z28407_at, L00058_at, U41060_at, X07696_at, J00124_at, J02611_at, C16161_s_at, U85658_at, S71043_rna1_s_at, HG2465-HT4871_at | Breast |
| 2 | X07730_at, R-C_AA176975_s_at, M24902_at, M34376_s_at, RC_AA449455_at, HG2261-HT2351_s_at, J03241_s_at, U22178_s_at, D29954_at, R-C_AA418020_at | X07730_at, R-C_AA176975_s_at, M24902_at, M34376_s_at, RC_AA449455_at, HG2261-HT2351_s_at, J03241_s_at, U22178_s_at, D29954_at, R-C_AA418020_at | Prostate |
| 3 | J03890_rna1_at, U58496_s_at, M68519_rna1_at, U89336_cds3_at, Y09267_at, HG2809-HT2920_s_at, M24461_at, U20391_rna6_at, W25781_at, AA258463_at | J03890_rna1_at, M68519_rna1_at, AA258463_at, U58496_s_at, U89336_cds3_at, H02425_at, HG2809-HT2920_s_at, R-C_AA045775_at, M24461_at, Y09267_at | Lung |
| 4 | RC_AA304344_f_at, W25945_at, AA459160_at | RC_AA256668_at, D00632_at, U50743_at, RC_AA252209_at, RC_AA490232_at, RC_AA477252_at, H72388_at, H78628_at, RC_AA155763_at, R-C_AA293123_s_at | kidney |
| 5 | U22376_cds2_s_at, S71043_rna1_s_at, R-C_AA290679_at, R-C_AA253471_at, AB006781_s_at, M35252_at, D14520_at, M93036_at, R-C_AA404487_at, X83228_at | U22376_cds2_s_at, RC_AA290679_at, RC_AA253471_at, AB006781_s_at, D14520_at, RC_AA404487_at, U55853_at, U55853_at.dup1, X83228_at, M35252_at | Colon |

Table 41: Top 10 genes of the modules of the normal tissues identified by $\mathcal{E}^2$ and the true types -1

| Modules identified by $\mathcal{E}^2$ | Top 10 genes, normal tissues, identified by $\mathcal{E}^2$ | Top 10 genes of true types | Type |
|---|---|---|---|
| 6 | RC_AA011598_at, W07209_at, R-C_AA477541_at, R-C_AA251759_at, R-C_AA521073_at, U06711_s_at, R-C_AA234384_s_at, X81832_s_at, X16666_s_at, X16666_s_at.dup1 | X13839_at, R-C_AA133215_at, R-C_AA281295_at, R-C_AA424567_at, AA374109_at, R-C_AA287097_at, R-C_AA195660_at, R-C_AA495994_at, W27425_at, R-C_AA348466_s_at | Uterus |
| 7 | M63438_s_at, AFFX-HSAC07/X00351_M_at, S62696_s_at, R-C_AA045249_at, X12530_s_at, X64044_at, M87789_s_at, AA291334_at, U35835_s_at, M63928_at | M63438_s_at, AFFX-HSAC07/X00351_M_at, S62696_s_at, R-C_AA045249_at, X12530_s_at, X64044_at, M87789_s_at, AA291334_at, U35835_s_at, M63928_at | Germinal |
| 8 | X13839_at, AF001548_rna1_at, M12125_at, Z24727_at, RC_AA233257_at, D00654_at, H41842_at, U52969_at, R01783_at, RC_AA453437_at | X69090_at, J00073_at, RC_AA262351_f_at, D90209_at, M59979_at, U52969_at, R-C_AA448863_at, R-C_AA430036_at, Z24727_at, R-C_AA598397_at | Bladder |
| 9 | M22612_f_at, M54994_f_at, D83847_f_at, M27602_f_at, AA426304_r_at, X67318_at, X80026_at, S82198_at, M89473_at, M21056_at | D83847_f_at, M54994_f_at, D49742_at, M22612_f_at, M27602_f_at, AA426304_r_at, X80026_at, M89473_at, AA312078_at, X16666_s_at | Pancreas |
| 10 | M55998_s_at, L49169_at, AA292440_s_at, R-C_AA253390_s_at, AA393106_at, U32907_at, L27559_s_at, R-C_AA621485_at, L13740_at, AFFX-BioDn-3_at | AA292440_s_at, L49169_at, M55998_s_at, U32907_at, RC_AA621485_at, RC_AA412505_at, L13740_at, L27559_s_at, M11433_at, AA287815_at | Ovary |

Table 42: Top 10 genes of the modules of the normal tissues identified by $\mathcal{E}^2$ and the true types -2

| Modules identified by $\mathcal{E}^2$ | Top 10 genes, normal tissues, identified by $\mathcal{E}^2$ | Top 10 genes of true types | Type |
|---|---|---|---|
| 11 | T55959_s_at, RC_AA236013_at, U45285_at, RC_AA281074_at, L20941_at, RC_AA293436_s_at, D30930_s_at, U03754_f_at, U51240_at, RC_AA279294_at | T55959_s_at, RC_AA236013_at, U45285_at, RC_AA281074_at, L20941_at, RC_AA293436_s_at, D30930_s_at, U03754_f_at, U51240_at, RC_AA279294_at | Peripheral |
| 12 | D00632_at, U50743_at, RC_AA256668_at, D56411_at, X69699_at, W02027_s_at, AA253330_s_at, RC_AA460234_at, RC_AA253331_at, H78628_at | RC_AA256668_at, D00632_at, U50743_at, RC_AA252209_at, RC_AA490232_at, RC_AA477252_at, H72388_at, H78628_at, RC_AA155763_at, RC_AA293123_s_at | Kidney |
| 13 | RC_AA424140_at, H19063_at, RC_D53092_at, RC_AA430033_at, D54949_at, R52420_at, H91747_s_at, M63379_at, W26436_s_at, U61849_s_at | Whole: RC_AA001220_at, W26520_at, W26436_s_at, X90846_at, W26340_at, AA095119_at, RC_AA256268_at, RC_AA610082_at, L05512_at, U88898_r_at, Cerebellum: D78012_at, R14091_at, RC_AA620289_at, RC_AA461162_at, U63455_at, AA402288_at, D87458_at, AA448497_at, RC_AA400047_at, M63379_at, | Whole, Cerebellum |

Table 43: Top 10 genes of the modules of the normal tissues identified by $\mathcal{E}^2$ and true types -3

| Module | Submodule | Cell type |
| --- | --- | --- |
| 1 | Breast_BR_1, Breast_BR_3 | |
| | Breast_BR_2, Breast_BR_4, Breast_93_I_184 | |
| 2 | Prostate_PR_2, Prostate_NLP10N, Prostate_NLP1N | |
| | Prostate_PR_5, Prostate_95_I_255, Prostate_PR_6 | |
| | Prostate_PR_3, Prostate_PR_7, Prostate_PR_4 | |
| 3 | Lung_LU_1, Lung_LU_2 | |
| | Lung_LU_7, Lung_LU_6, Lung_LU_3 | |
| 4 | Lung_93_I_028_(I), Lung_HCTN_LUN2_(18763_A2A) | |
| | Kidney_KT_5, Kidney_Norm_613NPS, Kidney_Norm_613NS | |
| 5 | Colon_CR_1, Colon_CR_3, Colon_CR_4 | |
| | Colon_9912c071_CC, Colon_CR_9, Colon_CR_6 | |
| | Colon_CR_5, Colon_CR_8, Colon_CR_10 | |
| 6 | Colon_CR_2, Colon_CR_7 | |
| | Uterus_UT_1, Uterus_UT_5 | |
| 7 | Germinal_Center_GC2, Germinal_Center_GC3 | |
| | Germinal_Center_GC45, Germinal_Center_GC48 | |
| | Germinal_Center_GC44, Germinal_Center_GC38 | |
| 8 | Bladder_BL_1, Bladder_BL_4, Bladder_BL_2, Bladder_95_I_285 | |
| | Whole_Brain_BRAIN_3 | |
| | Bladder_BL_3, Uterus_UT_2, Uterus_UT_8, Uterus_UT_3 | |
| 9 | Bladder_BL_5, Bladder_BL_6, Pancreas_PAN_1, Pancreas_PAN_2 | |
| | Pancreas_Pan_8N, Pancreas_Pan_11N, Pancreas_Pan_14N, Pancreas_Pan_43N, Pancreas_Pan_40N | |
| | Pancreas_Pan_42N, Pancreas_Pan13N, Pancreas_Pan_41N | |
| 10 | Uterus_UT_4 | |
| | Ovary_OV_1, Ovary_OV_2, Ovary_OV_4 | |
| | Ovary_OV_3 | |
| 11 | Peripheral_Blood_Poly_POLY1, Peripheral_Blood_Poly_POLY3, Peripheral_Blood_Poly_POLY2 | |
| | Peripheral_Blood_Mono_MONO5, Peripheral_Blood_Mono_MONO6 | |
| 12 | Kidney_KT_1, Kidney_KT_3, Kidney_Norm_627, Kidney_KT_6 | |
| | Kidney_KT_4, Kidney_KT_2 | |
| | Kidney_Norm_622NO, Kidney_Norm_613NO, Kidney_Norm_627NS | |
| 13 | Whole_Brain_BRAIN_1, Whole_Brain_BRAIN_4, Whole_Brain_BRAIN_5, Whole_Brain_BRAIN_2 | |
| | Cerebellum_Ncer_NCB1, Cerebellum_Brain_Ncer_S-51, Cerebellum_Brain_Ncer_S-125 | |

Table 44: The modules and submodules of normal tissues identified by $\mathcal{E}^3$.

| Submodules | Top 10 genes, normal tissues, identified by $\mathcal{E}^3$ |
|---|---|
| 1.1 | L00058_at, X13766_s_at, AA428090_at, X78416_s_at, C16161_s_at, J00124_at, AA247685_at, S83198_at, S83198_at.dup1, U51899_rna1_s_at |
| 1.2 | U84487_at, X84707_rna1_at, U31201_cds2_s_at, HG3543-HT3739_at, M29971_at, S80437_s_at, J00116_s_at, X52520_at, M13955_at, Z48633_at |
| 2.1 | U78294_at, RC_AA411796_at, U28687_at, U28687_at.dup1, U82613_at, U82613_at.dup1, U00943_at, U00943_at.dup1, RC_AA007169_at |
| 2.2 | X07730_at, S39329_at, N40141_at, HG2261-HT2352_at, U35735_at, U02619_at, U42360_cds2_at, J00123_at |
| 2.3 | RC_AA176975_s_at, RC_AA010665_at, R-C_AA449455_at, U07559_at, U22178_s_at, M24902_at, RC_AA609053_at, RC_AA370353_at, D85181_at, M34376_s_at |
| 3.1 | M68519_rna1_at, Y09267_at, U58496_s_at, M24461_at, U20391_rna6_at, M30838_at, U52100_at, X04470_s_at, HG2809-HT2920_s_at, X57809_s_at |
| 3.2 | J03890_rna1_at, RC_AA011176_at, U59914_at, W25781_at, RC_AA412681_at, RC_AA035613_at, N56191_at, AA365742_s_at, H49440_at, R-C_AA453619_at |
| 4.1 | F15201_at, W25945_at, X16666_s_at, X16666_s_at.dup1, RC_AA280043_at, RC_D20846_at, R81768_at, AA203297_at, R80351_at |
| 4.2 | RC_AA304344_f_at |
| 5.1 | M93036_at, AA427468_s_at, RC_AA053660_at, U07969_s_at, Y00339_s_at, S71043_rna1_s_at, U29091_at, M35252_at, AB006781_s_at, R-C_AA253471_at |
| 5.2 | RC_AA404338_at, AA443499_f_at, RC_AA398423_at, AA306768_at, RC_AA478971_r_at |
| 5.3 | U77180_at, J04813_s_at |

Table 45: Top 10 genes of submodules of the normal tissues identified by $\mathcal{E}^3$-1

| Submodule | Top 10 genes, normal tissues, identified by $\mathcal{E}^3$ |
|---|---|
| 6.1 | RC_AA011598_at, W07209_at, RC_AA251759_at, R-C_AA477541_at, X81832_s_at, HG2171-HT2241_at |
| 6.2 | M29335_at |
| 7.1 | M63438_s_at, X66087_at, U49395_at, U31556_at, M37238_s_at, U07804_s_at, X14850_at, X63380_at, U39817_at, U10485_at |
| 7.2 | AA176115_at, M84371_rna1_s_at, M84371_rna1_s_at.dup1, RC_AA115979_at, R-C_AA045249_at, M89957_at, M89957_at.dup1, H81497_at, RC_AA283907_at, RC_AA608545_at |
| 7.3 | HG3264-HT3441_at, T08870_at |
| 8.1 | J00073_at, M59979_at, U52969_at, U70370_at, U70370_at.dup1, U06711_s_at, X06256_at, AA397610_at, N31013_at, U16997_at |
| 8.2 | X99268_at, Z48804_at, AFFX-HSAC07/X00351_3_st, AA095119_at, N24990_s_at, RC_AA487879_at, RC_AA521073_at, X13839_at, AFFX-HUMGAPDH/M33197_3_st, HG1699-HT1704_s_at |
| 8.3 | M55998_s_at, AF001548_rna1_at, L27559_s_at, R-C_AA448863_at, RC_AA233257_at, M12125_at, R-C_AA453437_at, RC_AA151333_at, RC_AA133215_at, RC_AA621440_at |
| 9.1 | M54994_f_at, M21056_at, X67318_at, X71345_f_at, M22612_f_at, M24400_at, M27602_f_at, S82198_at, D83847_f_at, RC_AA481862_at |
| 9.2 | AFFX-HUMRGE/M10098_5_at, RC_AA255487_at, AA312078_at, RC_AA192760_at, AA292466_at, M30625_s_at |
| 9.3 | U66061_cds3_at, D49742_at, M89473_at, HG4194-HT4464_at, X54457_s_at, AA397763_at, R-C_AA282986_at, AFFX-BioDn-5_at, T52988_s_at |

Table 46: Top 10 genes of the submodules of the normal tissues identified by $\mathcal{E}^3$-2

| Submodule | Top 10 genes, normal tissues, identified by $\mathcal{E}^3$ |
|---|---|
| 10.1 | RC_AA262351_f_at, RC_AA424567_at, RC_AA045775_at, RC_AA443667_at, RC_AA495994_at, RC_AA403296_at, RC_AA253390_s_at, W28151_at, X82209_at, HG3111-HT3287_at |
| 10.2 | U32907_at, L49169_at, RC_AA412505_at, RC_AA621485_at, L13740_at, M13981_at, AA287815_at, RC_AA084286_at, M11433_at, AA410325_at |
| 10.3 | AA399299_at, U90918_at, U90918_at.dup1, AFFX-CreX-3_at, R12538_at, X16869_s_at, RC_AA453514_at |
| 11.1 | RC_AA236013_at, RC_AA357189_at, R80083_at, M32315_at, M32315_at.dup1, RC_AA280630_at, AA479826_at, RC_AA281074_at, M95767_at, M95767_at.dup1 |
| 11.2 | X64072_s_at, M57710_at, AF006084_at, U45285_at, D30930_s_at, M19722_at, T55959_s_at, U51240_at, HG2059-HT2114_at, Y00636_at |
| 12.1 | M11437_cds1_at, M26901_s_at, U50743_at, U26726_at, X76223_s_at, RC_AA253331_at, RC_AA256668_at, X92744_at, X69699_at, X04571_at |
| 12.2 | W02027_s_at |
| 12.3 | D00632_at, RC_AA121123_at, H42262_at, L20492_s_at, W07723_at, U58130_at, H72388_at, M36653_s_at, U90545_at, AA504692_at |
| 13.1 | RC_AA001220_at, RC_AA610082_at, RC_D59675_i_at, H19063_at, W26520_at, W05585_at, D28113_s_at, H46831_at, W26436_s_at, T34896_s_at |
| 13.2 | D78012_at, R14091_at, RC_AA620289_at, RC_AA461162_at, U63455_at, AA402288_at, D87458_at, AA448497_at, RC_AA400047_at, M63379_at |

Table 47: Top 10 genes of the submodules of the normal tissues identified by $\mathcal{E}^3$-3

# DLBCL Submodules identified by $\mathcal{E}^2$ and $\mathcal{E}^3$ Define Prognostic Categories

We use the DLBCL clinic data in Alizadeh, A. et al [2] to analyse the overall survival time, alive ratio and the International Prognostic Index score of the submodules of the DLBCL type by the algorithms $\mathcal{E}^2$, $\mathcal{E}^3$, $\mathcal{I}$ and $\mathcal{M}$, respectively.

## Submodules identified by $\mathcal{E}^2$

Tables 48 and 49 describe the statistical survival times, survival ratios and IPI scores of the DLBCL submodules identified by $\mathcal{E}^2$.

## Submodules identified by $\mathcal{E}^3$

In tables 50 and 51, we describe the statistical survival times, survival ratios and IPI scores of the DLBCL submodules identified by $\mathcal{E}^3$.

## Submodules identified by $\mathcal{I}$

In Tables 52 and 53, we describe the statistical survival times, survival ratios and IPI scores of the DLBCL submodules identified by $\mathcal{I}$.

## Submodules identified by $\mathcal{M}$

Table 54 describes the statistical survival times, survival ratios and IPI scores of the DLBCL submodules identified by $\mathcal{M}$.

| Submodules | Sample | Survival months | Survival indicator 0=alive 1=dead | IPI NA=Not Available |
|---|---|---|---|---|
| 1 | DLCL-0041 | 31.47 | 1 | 2 |
| | Average | 31.47 | 0% | 2 |
| 2 | DLCL-0042 | 39.60 | 1 | 1 |
| | DLCL-0007 | 8.3 | 1 | 3 |
| | DLCL-0031 | 12.3 | 1 | 2 |
| | DLCL-0036;OCT | 12.67 | 1 | NA |
| | DLCL-0025 | 32.5 | 1 | NA |
| | DLCL-0040 | 53.73 | 0 | 2 |
| | DLCL-0017 | 2.4 | 1 | 3 |
| | DLCL-0028 | 90.2 | 0 | 2 |
| | DLCL-0012 | 4.1 | 1 | 2 |
| | DLCL-0021 | 4.6 | 1 | 2 |
| | Average | 26.04 | 20% | 2.13 |
| 3 | DLCL-0030 | 71.3 | 0 | 1 |
| | DLCL-0011 | 27.1 | 1 | 4 |
| | DLCL-0020 | 80.4 | 0 | 1 |
| | DLCL-0032 | 69.1 | 0 | 4 |
| | DLCL-0033 | 68.8 | 0 | 2 |
| | DLCL-0003 | 71.3 | 1 | 2 |
| | DLCL-0034 | 1.3 | 1 | 4 |
| | DLCL-0051 | NA | NA | NA |
| | DLCL-0001 | 77.4 | 0 | 2 |
| | DLCL-0018 | 2.9 | 1 | 3 |
| | DLCL-0037 | 72.03 | 0 | 1 |
| | DLCL-0010 | 4.1 | 1 | 2 |
| | Average | 49.61 | 55% | 2.5 |

Table 48: Overall survival times, survival ratios and IPI scores of the DLBCL subtypes identified by $\mathcal{E}^2$-1

| Submodule | Sample | Survival months | Survival indicator 0=alive 1=dead | IPI NA=Not Available |
|---|---|---|---|---|
| 4 | DLCL-0004 | 69.6 | 0 | 0 |
| | DLCL-0029 | 83.8 | 0 | 0 |
| | DLCL-0008 | 202.4 | 0 | 1 |
| | DLCL-0052 | NA | NA | NA |
| | Average | 85.27 | 100% | 0.33 |
| 5 | DLCL-0006 | 3.2 | 1 | 3 |
| | DLCL-0049 | 22.3 | 1 | 2 |
| | DLCL-0039 | 91.33 | 0 | 3 |
| | DLCL-0002 | 3.4 | 1 | 3 |
| | Average | 30.06 | 25% | 2.75 |
| 6 | DLCL-0015 | 56.6 | 0 | 0 |
| | DLCL-0026 | 11.8 | 1 | 3 |
| | DLCL-0005 | 51.2 | 0 | 1 |
| | DLCL-0023 | 8.2 | 1 | 0 |
| | DLCL-0027 | 5.1 | 1 | 3 |
| | DLCL-0024 | 129.9 | 0 | 1 |
| | DLCL-0013 | 23.7 | 1 | 4 |
| | DLCL-0016 | 15.5 | 1 | 4 |
| | DLCL-0014 | 59 | 0 | 1 |
| | DLCL-0048 | 9.45 | 1 | 0 |
| | Average | 37.04 | 40% | 1.7 |
| 9 | DLCL-0009 | 89.8 | 0 | 0 |
| | Average | 89.8 | 100% | 0 |

Table 49: Overall survival times, survival ratios and IPI scores of the DLBCL subtypes identified by $\mathcal{E}^2$-2

| Submodules | Sample | Survival months | Survival indicator 0=alive 1=dead | IPI NA=Not Available |
|---|---|---|---|---|
| 1.2 | DLCL-0041 | 31.47 | 1 | 2 |
| | Average | 31.47 | 0% | 2 |
| 2.1 | DLCL-0042 | 39.60 | 1 | 1 |
| | Average | 39.60 | 0% | 1 |
| 2.2 | DLCL-0007 | 8.3 | 1 | 3 |
| | DLCL-0031 | 12.3 | 1 | 2 |
| | Average | 10.3 | 0% | 2.5 |
| 3.1 | DLCL-0036;OCT | 12.67 | 1 | NA |
| | DLCL-0025 | 32.5 | 1 | NA |
| | DLCL-0028 | 90.2 | 0 | 2 |
| | Average | 45.12 | 33% | 2 |
| 3.2 | DLCL-0040 | 53.73 | 0 | 2 |
| | DLCL-0017 | 2.4 | 1 | 3 |
| | Average | 28.065 | 50% | 2.5 |
| 3.3 | DLCL-0012 | 4.1 | 1 | 2 |
| | DLCL-0021 | 4.6 | 1 | 2 |
| | Average | 4.35 | 0% | 2 |
| 4.1 | DLCL-0030 | 71.3 | 0 | 0 |
| | DLCL-0032 | 69.1 | 0 | 4 |
| | DLCL-0051 | NA | NA | NA |
| | Average | 70.2 | 100% | 2 |
| 4.2 | DLCL-0011 | 27.1 | 1 | 4 |
| | DLCL-0020 | 80.4 | 0 | 1 |
| | Average | 53.25 | 50% | 2.5 |
| 4.3 | DLCL-0033 | 68.8 | 0 | 2 |
| | DLCL-0034 | 1.3 | 1 | 4 |
| | DLCL-0003 | 71.3 | 1 | 2 |
| | Average | 47.13 | 33% | 2.67 |
| 5.1 | DLCL-0004 | 69.6 | 0 | 0 |
| | DLCL-0029 | 83.8 | 0 | 0 |
| | Average | 76.7 | 100% | 0 |
| 5.2 | DLCL-0008 | 102.4 | 0 | 1 |
| | DLCL-0052 | NA | NA | NA |
| | Average | 102.4 | 100% | 1 |

Table 50: Overall survival times, survival ratios and IPI scores of the DLBCL subtypes identified by $\mathcal{E}^3$-1

| Submodule | Sample | Survival months | Survival indicator 0=alive 1=dead | IPI NA=Not Available |
|---|---|---|---|---|
| 6.1 | DLCL-0006 | 3.2 | 1 | 3 |
| | DLCL-0002 | 3.4 | 1 | 3 |
| | Average | 3.3 | 0% | 3 |
| 6.2 | DLCL-0049 | 22.3 | 1 | 2 |
| | DLCL-0039 | 91.33 | 0 | 3 |
| | Average | 56.82 | 50% | 2.5 |
| 7.1 | DLCL-0001 | 77.4 | 0 | 2 |
| | DLCL-0018 | 2.9 | 1 | 3 |
| | Average | 40.15 | 50% | 2.5 |
| 7.2 | DLCL-0037 | 72.03 | 0 | 1 |
| | DLCL-0010 | 4.1 | 1 | 3 |
| | Average | 38.07 | 50% | 2 |
| 8.1 | DLCL-0015 | 56.6 | 0 | 0 |
| | DLCL-0005 | 51.2 | 0 | 1 |
| | DLCL-0013 | 23.7 | 1 | 4 |
| | Average | 43.83 | 67% | 1.67 |
| 8.2 | DLCL-0026 | 11.8 | 1 | 3 |
| | DLCL-0027 | 5.1 | 1 | 3 |
| | DLCL-0024 | 129.9 | 0 | 1 |
| | DLCL-0023 | 8.2 | 1 | 0 |
| | Average | 38.75 | 25% | 1.75 |
| 8.3 | DLCL-0016 | 15.5 | 1 | 4 |
| | DLCL-0014 | 59 | 0 | 1 |
| | DLCL-0048 | 9.45 | 1 | 0 |
| | Average | 27.98 | 33% | 1.67 |
| 11.3 | DLCL-0009 | 89.9 | 0 | 0 |
| | Average | 89.9 | 100% | 0 |

Table 51: Overall survival times, survival ratios and IPI scores of the DLBCL subtypes identified by $\mathcal{E}^3$-2

| Submodule | Sample | Survival months | Survival indicator 0=alive 1=dead | IPI NA=Not Available |
|---|---|---|---|---|
| 1 | DLCL-0030 | 71.3 | 0 | 0 |
| | DLCL-0004 | 69.6 | 0 | 0 |
| | DLCL-0029 | 83.3 | 0 | 0 |
| | DLCL-0008 | 102.4 | 0 | 1 |
| | DLCL-0052 | NA | NA | NA |
| | DLCL-0034 | 1.3 | 1 | 4 |
| | DLCL-0051 | NA | NA | NA |
| | DLCL-0011 | 27.1 | 1 | 4 |
| | DLCL-0032 | 69.1 | 0 | 4 |
| | DLCL-0006 | 3.2 | 1 | 3 |
| | DLCL-0049 | 22.3 | 1 | 2 |
| | DLCL-0039 | 91.33 | 0 | 3 |
| | DLCL-0001 | 77.4 | 0 | 2 |
| | DLCL-0018 | 2.9 | 1 | 3 |
| | DLCL-0037 | 72.03 | 0 | 1 |
| | DLCL-0010 | 88.1 | 0 | 3 |
| | DLCL-0015 | 56.6 | 0 | 0 |
| | DLCL-0026 | 11.8 | 1 | 3 |
| | DLCL-0005 | 51.2 | 0 | 1 |
| | DLCL-0023 | 8.2 | 1 | 0 |
| | DLCL-0027 | 5.1 | 1 | 3 |
| | DLCL-0024 | 129.9 | 0 | 1 |
| | DLCL-0013 | 23.7 | 1 | 4 |
| | DLCL-0002 | 3.4 | 1 | 3 |
| | DLCL-0016 | 15.5 | 1 | 4 |
| | DLCL-0020 | 80.4 | 0 | 1 |
| | DLCL-0003 | 71.3 | 1 | 1 |
| | DLCL-0014 | 59 | 0 | 1 |
| | DLCL-0048 | 9.45 | 1 | 0 |
| | DLCL-0033 | 68.8 | 0 | 2 |
| | DLCL-0012 | 4.1 | 1 | 2 |
| | Average | 47.58 | 52% | 1.97 |

Table 52: Overall survival times, survival ratios and IPI scores of the DLBCL subtypes identified by $\mathcal{I}$-1

| Submodule | Sample | Survival months | Survival indicator 0=alive 1=dead | IPI NA=Not Available |
|---|---|---|---|---|
| 3 | DLCL-0009 | 89.8 | 0 | 0 |
| | Average | 89.8 | 100% | 0 |
| 6 | DLCL-0041 | 31.47 | 1 | 2 |
| | Average | 31.47 | 0% | 2 |
| 7 | DLCL-0036:OCT | 12.67 | 1 | NA |
| | DLCL-0025 | 32.5 | 1 | NA |
| | DLCL-0040 | 53.73 | 0 | 2 |
| | DLCL-0017 | 2.4 | 1 | 3 |
| | DLCL-0028 | 90.2 | 0 | 2 |
| | DLCL-0021 | 4.6 | 1 | 2 |
| | Average | 32.68 | 33% | 2.25 |
| 8 | DLCL-0042 | 39.6 | 1 | 1 |
| | DLCL-0007 | 8.3 | 1 | 3 |
| | DLCL-0031 | 12.3 | 1 | 2 |
| | Average | 20.07 | 0% | 2 |

Table 53: Overall survival times, survival ratios and IPI scores of the DLBCL subtypes identified by $\mathcal{I}$-2

| Module | Samples | Average Survival Months | Alive ratio | Average IPI |
|---|---|---|---|---|
| 1 | DLCL-0041, DLCL-0042, DLCL-0007, DLCL-0031, DLCL-0036;OCT, DLCL-0017, DLCL-0025, DLCL-0028, DLCL-0040, DLCL-0021, DLCL-0012, DLCL-0014, DLCL-0003, DLCL-0010, DLCL-0018, DLCL-0030, DLCL-0015, DLCL-0037, DLCL-0052, DLCL-0051, DLCL-0004, DLCL-0008, DLCL-0001, DLCL-0034, DLCL-0032, DLCL-0029, DLCL-0020, DLCL-0033, DLCL-0048, DLCL-0016, DLCL-0023, DLCL-0011, DLCL-0006, DLCL-0002, DLCL-0005, DLCL-0013, DLCL-0027, DLCL-0026, DLCL-0024, DLCL-0049, DLCL-0039 | 42.77 | 44% | 2 |
| 2 | DLCL-0009 | 89.8 | 100% | 0 |

Table 54: Overall survival times, survival ratios and IPI scores of the DLBCL subtypes identified by $\mathcal{M}$

# New Test Leukemia Data

## True types

Tables 55 - 61 describe the true types of the new test leukemia data.

| Community | Samples | Cell type |
|---|---|---|
| 1 | GSM332115, GSM332268, GSM332305, GSM332418, GSM332611 | C1 |
| 2 | GSM331786, GSM331831, GSM331894, GSM331964, GSM331968, GSM331993, GSM332052, GSM332136, GSM332192, GSM332205, GSM332240, GSM332291, GSM332293, GSM332554, GSM332577, GSM332639, GSM332723, GSM332738, GSM332759, GSM332786, GSM332811, GSM332842, GSM332847 | C2 |
| 3 | GSM331736, GSM331760, GSM331784, GSM331792, GSM331798, GSM331822, GSM331842, GSM331847, GSM331849, GSM331872, GSM331900, GSM331907, GSM331916, GSM331942, GSM331956, GSM332005, GSM332012, GSM332031, GSM332039, GSM332079, GSM332153, GSM332173, GSM332177, GSM332193, GSM332195, GSM332222, GSM332231, GSM332236, GSM332239, GSM332252, GSM332307, GSM332353, GSM332357, GSM332368, GSM332376, GSM332406, GSM332454, GSM332499, GSM332500, GSM332513, GSM332523, GSM332540, GSM332545, GSM332553, GSM332555, GSM332557, GSM332573, GSM332583, GSM332592, GSM332604, GSM332667, GSM332678, GSM332681, GSM332687, GSM332694, GSM332706, GSM332712, GSM332730, GSM332790, GSM332837, GSM332846, GSM332858 | C3 |
| 4 | GSM331737, GSM331746, GSM331802, GSM331819, GSM331820, GSM331850, GSM331858, GSM331863, GSM331864, GSM331871, GSM331882, GSM331885, GSM331886, GSM331887, GSM331904, GSM331905, GSM331908, GSM331910, GSM331983, GSM331985, GSM332003, GSM332017, GSM332051, GSM332058, GSM332060, GSM332104, GSM332108, GSM332119, GSM332137, GSM332143, GSM332151, GSM332156, GSM332162, GSM332167, GSM332200, GSM332214, GSM332232, GSM332267, GSM332274, GSM332295, GSM332318, GSM332333, GSM332338, GSM332342, GSM332356, GSM332364, GSM332386, GSM332390, GSM332398, GSM332415, GSM332427, GSM332465, GSM332472, GSM332474, GSM332478, GSM332480, GSM332494, GSM332570, GSM332590, GSM332613, GSM332618, GSM332626, GSM332628, GSM332635, GSM332641, GSM332670, GSM332690, GSM332741, GSM332744, GSM332753, GSM332762, GSM332781, GSM332783, GSM332794, GSM332806, GSM332819, GSM332854, GSM332870, GSM332878 | C4 |

Table 55: Real modules of cell samples of Microarray Innovations in Leukemia-1

| Community | Samples | Cell type |
|---|---|---|
| 5 | GSM331739, GSM331741, GSM331757, GSM331774, GSM331821, GSM331870, GSM331891, GSM331927, GSM331939, GSM331951, GSM331959, GSM331969, GSM331996, GSM332025, GSM332029, GSM332036, GSM332059, GSM332094, GSM332109, GSM332133, GSM332135, GSM332141, GSM332152, GSM332168, GSM332216, GSM332266, GSM332288, GSM332299, GSM332308, GSM332321, GSM332365, GSM332370, GSM332388, GSM332426, GSM332438, GSM332442, GSM332447, GSM332449, GSM332462, GSM332466, GSM332476, GSM332479, GSM332487, GSM332493, GSM332522, GSM332528, GSM332538, GSM332569, GSM332580, GSM332582, GSM332644, GSM332680, GSM332745, GSM332746, GSM332747, GSM332768, GSM332772, GSM332776, GSM332785, GSM332801, GSM332815, GSM332834, GSM332839, GSM332881 | C5 |
| 6 | GSM331878, GSM332067, GSM332123, GSM332313, GSM332457, GSM332629, GSM332638, GSM332652, GSM332699, GSM332877, | C6 |
| 7 | GSM331733, GSM331754, GSM331756, GSM331766, GSM331826, GSM331965, GSM331966, GSM332004, GSM332008, GSM332145, GSM332157, GSM332198, GSM332210, GSM332218, GSM332224, GSM332262, GSM332263, GSM332281, GSM332312, GSM332335, GSM332337, GSM332340, GSM332458, GSM332504, GSM332568, GSM332591, GSM332646, GSM332654, GSM332697, GSM332713, GSM332714, GSM332716, GSM332797, GSM332817, GSM332818 | C7 |

Table 56: Real modules of cell samples of Microarray Innovations in Leukemia-2

| Community | Samples | Cell type |
|---|---|---|
| 8 | GSM331735, GSM331744, GSM331749, GSM331753, GSM331755, GSM331763, GSM331764, GSM331771, GSM331777, GSM331780, GSM331781, GSM331788, GSM331796, GSM331800, GSM331810, GSM331812, GSM331817, GSM331818, GSM331828, GSM331833, GSM331844, GSM331846, GSM331848, GSM331884, GSM331896, GSM331898, GSM331899, GSM331901, GSM331914, GSM331918, GSM331920, GSM331938, GSM331945, GSM331960, GSM331967, GSM331980, GSM331982, GSM331984, GSM331990, GSM331997, GSM331999, GSM332001, GSM332013, GSM332019, GSM332041, GSM332045, GSM332054, GSM332057, GSM332072, GSM332073, GSM332084, GSM332085, GSM332088, GSM332096, GSM332100, GSM332105, GSM332112, GSM332113, GSM332116, GSM332125, GSM332146, GSM332154, GSM332175, GSM332180, GSM332185, GSM332190, GSM332203, GSM332204, GSM332215, GSM332219, GSM332230, GSM332237, GSM332255, GSM332269, GSM332278, GSM332279, GSM332297, GSM332301, GSM332304, GSM332316, GSM332326, GSM332332, GSM332334, GSM332343, GSM332350, GSM332359, GSM332361, GSM332369, GSM332373, GSM332384, GSM332387, GSM332392, GSM332396, GSM332399, GSM332411, GSM332423, GSM332428, GSM332433, GSM332436, GSM332444, GSM332452, GSM332459, GSM332464, GSM332467, GSM332468, GSM332471, GSM332489, GSM332502, GSM332505, GSM332508, GSM332525, GSM332543, GSM332548, GSM332561, GSM332574, GSM332575, GSM332593, GSM332603, GSM332608, GSM332610, GSM332617, GSM332634, GSM332637, GSM332640, GSM332643, GSM332645, GSM332649, GSM332653, GSM332660, GSM332683, GSM332702, GSM332710, GSM332715, GSM332719, GSM332720, GSM332722, GSM332724, GSM332734, GSM332736, GSM332748, GSM332755, GSM332757, GSM332760, GSM332763, GSM332773, GSM332796, GSM332798, GSM332799, GSM332800, GSM332804, GSM332816, GSM332821, GSM332827, GSM332829, GSM332841, GSM332850, GSM332855, GSM332866 | C8 |
| 9 | GSM331751, GSM331790, GSM331995, GSM332027, GSM332097, GSM332140, GSM332243, GSM332298, GSM332302, GSM332380, GSM332542, GSM332571, GSM332587, GSM332662, GSM332805, GSM332874 | C9 |
| 10 | GSM331782, GSM331789, GSM331807, GSM331926, GSM331952, GSM331975, GSM332035, GSM332129, GSM332251, GSM332258, GSM332456, GSM332463, GSM332521, GSM332534, GSM332585, GSM332664, GSM332705, GSM332808, GSM332824, GSM332882 | C10 |

Table 57: Real modules of cell samples of Microarray Innovations in Leukemia-3

| Community | Samples | Cell type |
|---|---|---|
| 11 | GSM331830, GSM331866, GSM331974, GSM332032, GSM332053, GSM332055, GSM332124, GSM332139, GSM332182, GSM332249, GSM332264, GSM332282, GSM332371, GSM332412, GSM332422, GSM332431, GSM332441, GSM332460, GSM332516, GSM332787 | C11 |
| 12 | GSM331776, GSM331793, GSM331797, GSM331880, GSM331928, GSM332065, GSM332095, GSM332196, GSM332314, GSM332366, GSM332377, GSM332535, GSM332549, GSM332621, GSM332647, GSM332656, GSM332684 | C12 |
| 13 | GSM331745, GSM331747, GSM331765, GSM331769, GSM331770, GSM331787, GSM331791, GSM331795, GSM331804, GSM331832, GSM331834, GSM331839, GSM331851, GSM331856, GSM331865, GSM331875, GSM331881, GSM331888, GSM331892, GSM331895, GSM331897, GSM331921, GSM331931, GSM331932, GSM331941, GSM331948, GSM331954, GSM331955, GSM331961, GSM331971, GSM331981, GSM331988, GSM331989, GSM331998, GSM332010, GSM332018, GSM332038, GSM332047, GSM332071, GSM332075, GSM332083, GSM332086, GSM332089, GSM332098, GSM332101, GSM332110, GSM332111, GSM332120, GSM332128, GSM332130, GSM332138, GSM332144, GSM332147, GSM332149, GSM332150, GSM332155, GSM332163, GSM332174, GSM332179, GSM332187, GSM332188, GSM332202, GSM332212, GSM332213, GSM332229, GSM332238, GSM332241, GSM332244, GSM332254, GSM332260, GSM332261, GSM332265, GSM332272, GSM332275, GSM332289, GSM332317, GSM332319, GSM332328, GSM332345, GSM332347, GSM332348, GSM332351, GSM332355, GSM332358, GSM332362, GSM332363, GSM332379, GSM332382, GSM332385, GSM332389, GSM332393, GSM332395, GSM332397, GSM332401, GSM332409, GSM332416, GSM332424, GSM332435, GSM332437, GSM332440, GSM332443, GSM332450, GSM332461, GSM332482, GSM332484, GSM332488, GSM332492, GSM332495, GSM332515, GSM332517, GSM332530, GSM332537, GSM332546, GSM332576, GSM332588, GSM332598, GSM332601, GSM332609, GSM332616, GSM332620, GSM332624, GSM332630, GSM332650, GSM332651, GSM332665, GSM332668, GSM332672, GSM332677, GSM332685, GSM332688, GSM332691, GSM332696, GSM332703, GSM332704, GSM332707, GSM332709, GSM332726, GSM332731, GSM332743, GSM332754, GSM332767, GSM332774, GSM332777, GSM332780, GSM332782, GSM332792, GSM332802, GSM332803, GSM332809, GSM332814, GSM332833, GSM332843, GSM332848, GSM332851, GSM332852, GSM332862, GSM332863, GSM332871, GSM332873, GSM332880, | C13 |

Table 58: Real modules of cell samples of Microarray Innovations in Leukemia-4

| Community | Samples | Cell type |
|---|---|---|
| 14 | GSM331794, GSM331803, GSM331861, GSM331958, GSM332006, GSM332015, GSM332087, GSM332131, GSM332164, GSM332228, GSM332306, GSM332329, GSM332346, GSM332394, GSM332532, GSM332565, GSM332566, GSM332615, GSM332627, GSM332751, GSM332761, GSM332831, GSM332838, GSM332849 | C14 |
| 15 | GSM331740, GSM331752, GSM331759, GSM331761, GSM331762, GSM331767, GSM331768, GSM331773, GSM331778, GSM331783, GSM331805, GSM331806, GSM331808, GSM331809, GSM331811, GSM331814, GSM331816, GSM331825, GSM331827, GSM331843, GSM331854, GSM331860, GSM331867, GSM331868, GSM331873, GSM331874, GSM331876, GSM331889, GSM331893, GSM331902, GSM331906, GSM331913, GSM331915, GSM331917, GSM331929, GSM331930, GSM331934, GSM331937, GSM331944, GSM331946, GSM331947, GSM331950, GSM331957, GSM331963, GSM331970, GSM331972, GSM331973, GSM331976, GSM331977, GSM331978, GSM331987, GSM331994, GSM332002, GSM332007, GSM332011, GSM332014, GSM332016, GSM332020, GSM332021, GSM332022, GSM332023, GSM332026, GSM332028, GSM332037, GSM332040, GSM332043, GSM332046, GSM332048, GSM332049, GSM332064, GSM332069, GSM332070, GSM332074, GSM332076, GSM332077, GSM332078, GSM332081, GSM332082, GSM332090, GSM332091, GSM332092, GSM332093, GSM332114, GSM332117, GSM332121, GSM332122, GSM332158, GSM332159, GSM332161, GSM332165, GSM332166, GSM332169, GSM332170, GSM332171, GSM332172, GSM332176, GSM332178, GSM332181, GSM332183, GSM332184, GSM332186, GSM332189, GSM332191, GSM332194, GSM332201, GSM332206, GSM332208, GSM332217, GSM332221, GSM332223, GSM332226, GSM332233, GSM332234, GSM332250, GSM332253, GSM332257, GSM332273, GSM332276, GSM332287, GSM332290, GSM332300, GSM332303, GSM332309, GSM332315, GSM332320, GSM332322, GSM332324, GSM332327, GSM332331, GSM332336, GSM332341, GSM332352, GSM332354, GSM332367, GSM332375, GSM332381, GSM332391, GSM332405, GSM332408, GSM332413, | C15 |

Table 59: Real modules of cell samples of Microarray Innovations in Leukemia-5

| Community | Samples | Cell type |
|---|---|---|
| 15 | GSM332414, GSM332420, GSM332421, GSM332425, GSM332430, GSM332432, GSM332434, GSM332451, GSM332453, GSM332470, GSM332473, GSM332475, GSM332497, GSM332503, GSM332507, GSM332510, GSM332512, GSM332514, GSM332520, GSM332524, GSM332526, GSM332527, GSM332531, GSM332533, GSM332539, GSM332541, GSM332547, GSM332551, GSM332552, GSM332556, GSM332558, GSM332559, GSM332562, GSM332563, GSM332564, GSM332567, GSM332578, GSM332581, GSM332584, GSM332586, GSM332589, GSM332594, GSM332595, GSM332596, GSM332597, GSM332600, GSM332602, GSM332606, GSM332607, GSM332612, GSM332622, GSM332625, GSM332636, GSM332642, GSM332648, GSM332655, GSM332657, GSM332659, GSM332661, GSM332671, GSM332673, GSM332674, GSM332689, GSM332692, GSM332701, GSM332708, GSM332711, GSM332718, GSM332721, GSM332727, GSM332729, GSM332735, GSM332749, GSM332752, GSM332756, GSM332764, GSM332765, GSM332766, GSM332769, GSM332770, GSM332779, GSM332784, GSM332793, GSM332795, GSM332810, GSM332820, GSM332825, GSM332840, GSM332845, GSM332856, GSM332857, GSM332864, GSM332865, GSM332869, GSM332875, GSM332879, GSM332883 | C15 |
| 16 | GSM331836, GSM331838, GSM331869, GSM331877, GSM331883, GSM331943, GSM331986, GSM332062, GSM332099, GSM332102, GSM332106, GSM332132, GSM332134, GSM332142, GSM332160, GSM332199, GSM332242, GSM332256, GSM332280, GSM332286, GSM332383, GSM332404, GSM332419, GSM332483, GSM332518, GSM332599, GSM332623, GSM332632, GSM332663, GSM332666, GSM332669, GSM332675, GSM332698, GSM332725, GSM332737, GSM332758, GSM332789, GSM332812, GSM332828, GSM332830, GSM332860, GSM332867, GSM332868 | C16 |

Table 60: Real modules of cell samples of Microarray Innovations in Leukemia-6

| Community | Samples | Cell type |
|---|---|---|
| 17 | GSM331734, GSM331738, GSM331742, GSM331743, GSM331748, GSM331750, GSM331758, GSM331772, GSM331775, GSM331779, GSM331785, GSM331799, GSM331801, GSM331813, GSM331815, GSM331823, GSM331824, GSM331829, GSM331837, GSM331841, GSM331845, GSM331852, GSM331853, GSM331855, GSM331857, GSM331862, GSM331911, GSM331912, GSM331919, GSM331922, GSM331923, GSM331925, GSM331933, GSM331935, GSM331940, GSM331949, GSM331979, GSM331991, GSM331992, GSM332000, GSM332024, GSM332030, GSM332033, GSM332050, GSM332056, GSM332066, GSM332068, GSM332107, GSM332197, GSM332207, GSM332211, GSM332220, GSM332227, GSM332235, GSM332245, GSM332246, GSM332247, GSM332248, GSM332259, GSM332270, GSM332271, GSM332277, GSM332284, GSM332285, GSM332292, GSM332294, GSM332296, GSM332311, GSM332325, GSM332330, GSM332339, GSM332344, GSM332378, GSM332400, GSM332403, GSM332407, GSM332410, GSM332417, GSM332429, GSM332439, GSM332445, GSM332469, GSM332477, GSM332485, GSM332486, GSM332490, GSM332498, GSM332501, GSM332511, GSM332519, GSM332550, GSM332579, GSM332605, GSM332619, GSM332631, GSM332633, GSM332658, GSM332676, GSM332679, GSM332693, GSM332695, GSM332717, GSM332728, GSM332732, GSM332733, GSM332739, GSM332740, GSM332750, GSM332771, GSM332775, GSM332778, GSM332788, GSM332791, GSM332813, GSM332822, GSM332835, GSM332844, GSM332853, GSM332859, GSM332872, GSM332884 | C17 |
| 18 | GSM331835, GSM331840, GSM331859, GSM331879, GSM331890, GSM331903, GSM331909, GSM331924, GSM331936, GSM331953, GSM331962, GSM332009, GSM332034, GSM332042, GSM332044, GSM332061, GSM332063, GSM332080, GSM332103, GSM332118, GSM332126, GSM332127, GSM332148, GSM332209, GSM332225, GSM332283, GSM332310, GSM332323, GSM332349, GSM332360, GSM332372, GSM332374, GSM332402, GSM332446, GSM332448, GSM332455, GSM332481, GSM332491, GSM332496, GSM332506, GSM332509, GSM332529, GSM332536, GSM332544, GSM332560, GSM332572, GSM332614, GSM332682, GSM332686, GSM332700, GSM332742, GSM332807, GSM332823, GSM332826, GSM332832, GSM332836, GSM332861, GSM332876 | C18 |

Table 61: Real modules of cell samples of Microarray Innovations in Leukemia-7

## Types found by $\mathcal{M}$

Tables 62 - 68 describe the modules found by $\mathcal{M}$.

| Community | Samples | Cell type |
|---|---|---|
| 1 | GSM331733, GSM331735, GSM331736, GSM331788, GSM331818, GSM332548, GSM332617, GSM332634, GSM332297, GSM332230, GSM332269, GSM332796, GSM331920, GSM331878, GSM332123, GSM332067, GSM332638, GSM332652, GSM332313, GSM332457, GSM332629, GSM332699, GSM332877, GSM331846, GSM332154, GSM332710, GSM332847, GSM331872, GSM332072, GSM332057, GSM332255, GSM332683, GSM332702, GSM332146, GSM331980, GSM332332, GSM332373, GSM332583, GSM332722, GSM332720, GSM332343, GSM332561, GSM331828, GSM332706, GSM332307, GSM331907, GSM332039, GSM331956, GSM331849, GSM332005, GSM332543, GSM332454, GSM332734, GSM332804, GSM332436, GSM332100, GSM332850, GSM331960, GSM332575, GSM332113, GSM332712, GSM332459, GSM332175, GSM331771, GSM332222, GSM332215, GSM332502, GSM332239, GSM332858, GSM332204, GSM331792, GSM332088, GSM332116, GSM332760, GSM332748, GSM332084, GSM331763, GSM332334, GSM332724, GSM332645, GSM332580, GSM332444, GSM331844, GSM332557, GSM332045, GSM332464, GSM332185, GSM332237, GSM332369, GSM332468, GSM332219, GSM332041, GSM332467, GSM332335, GSM332610, GSM332406, GSM332513, GSM331777, GSM332153, GSM332827, GSM331899, GSM332800, GSM332190, GSM332553, GSM332231, GSM331900, GSM332653, GSM332523, GSM332837, GSM332687, GSM332112, GSM332730, GSM332694, GSM332326, GSM332757, GSM332073, GSM331800, GSM331914, GSM332433, GSM332471, GSM332855, GSM331898, GSM332263, GSM332085, GSM332012, GSM331916, GSM332500, GSM332545, GSM332357, GSM332821, GSM332763, GSM332031, GSM331939, GSM332799, GSM331842, GSM332376, GSM332340, GSM331822, GSM332681, GSM332195, GSM332173, GSM332573, GSM332790, GSM332846, GSM331942, GSM332604, GSM332592, GSM332236, GSM332079, GSM332252, GSM332667, GSM332353, GSM331847, GSM332678, GSM331760, GSM332499, GSM332555, GSM331784, GSM332177, GSM331798, GSM332525, GSM332368, GSM332608, GSM332001, GSM332714, GSM332755, GSM332841, GSM332719, GSM332654, GSM331848, | C2, C3, C4, C5, C6, C7, C8, C13 |

Table 62: Modules of cell samples of Microarray Innovations in Leukemia identified by $\mathcal{M}$-1

| Community | Samples | Cell type |
|---|---|---|
| 1 | GSM332603, GSM331896, GSM332649, GSM331990, GSM332715, GSM332428, GSM332218, GSM332180, GSM332697, GSM331892, GSM332817, GSM332337, GSM332054, GSM331826, GSM332004, GSM332716, GSM331781, GSM331967, GSM331999, GSM331965, GSM331766, GSM331754, GSM331901, GSM332593, GSM332312, GSM331796, GSM331966, GSM332411, GSM332143, GSM331884, GSM332458, GSM332773, GSM331780, GSM332504, GSM332713, GSM332797, GSM331891, GSM332145, GSM332157, GSM332262, GSM332818, GSM332224, GSM332193, GSM332798, GSM331756, GSM332210, GSM332281, GSM332359, GSM332646, GSM332198, GSM332591, GSM332568, GSM332489, GSM332301, GSM332361, GSM331753, GSM331810, GSM331945, GSM332384, GSM332316, GSM331755, GSM331764, GSM332399, GSM332350, GSM332392, GSM332096, GSM332637, GSM332829, GSM332279, GSM332452, GSM332203, GSM331938, GSM332019, GSM331833, GSM332660, GSM331984, GSM331744, GSM332423, GSM332574, GSM332508, GSM332866, GSM331749 | C2, C3, C4, C5, C6, C7, C8, C13 |
| 2 | GSM331734, GSM331738, GSM331750, GSM331954, GSM332358, GSM332389, GSM332849, GSM332397, GSM332627, GSM332609, GSM332187, GSM332698, GSM331835, GSM332292, GSM331861, GSM331841, GSM332378, GSM332861, GSM331979, GSM332220, GSM332615, GSM332807, GSM332544, GSM332080, GSM331801, GSM332496, GSM332225, GSM332876, GSM332509, GSM332550, GSM332619, GSM332429, GSM331940, GSM332832, GSM332536, GSM331953, GSM332044, GSM332788, GSM332448, GSM332271, GSM332349, GSM331857, GSM332682, GSM331769, GSM331903, GSM332446, GSM332501, GSM332602, GSM332823, GSM332042, GSM332491, GSM332323, GSM332310, GSM332836, GSM332826, GSM332700, GSM332372, GSM331962, GSM332009, GSM332259, GSM332658, GSM331772, GSM332211, GSM332529, GSM332830, GSM332111, GSM332439, GSM332791, GSM332410, GSM332402, GSM332506, GSM332455, GSM332686, GSM332103, GSM331924, GSM332481, GSM332261, GSM332285, GSM332268, GSM332115, GSM331779, GSM332731, GSM332485, GSM332164, GSM332624, GSM331923, GSM332532, GSM332284, GSM332851, GSM332348, | C1, C2, C3, C4, C5, C8, C11, C13, C14, C15, C16, C17, C18 |

Table 63: Modules of cell samples of Microarray Innovations in Leukemia identified by $\mathcal{M}$-2

| Community | Samples | Cell type |
|---|---|---|
| 2 | GSM332418, GSM331813, GSM332209, GSM331919, GSM332519, GSM332611, GSM332614, GSM332396, GSM331812, GSM332248, GSM331879, GSM332311, GSM332812, GSM332676, GSM332148, GSM332560, GSM332360, GSM331936, GSM332325, GSM331840, GSM332417, GSM332775, GSM332631, GSM332050, GSM331855, GSM331912, GSM332511, GSM331890, GSM332066, GSM332822, GSM332197, GSM331949, GSM332278, GSM332505, GSM332283, GSM332813, GSM332445, GSM331925, GSM332294, GSM332695, GSM331758, GSM332572, GSM332227, GSM332330, GSM332693, GSM331922, GSM332295, GSM332490, GSM332247, GSM331935, GSM332771, GSM331992, GSM332246, GSM331824, GSM332679, GSM332477, GSM332599, GSM331865, GSM332068, GSM332732, GSM332844, GSM332056, GSM332848, GSM332566, GSM332024, GSM332061, GSM332742, GSM331909, GSM332063, GSM332605, GSM332272, GSM332126, GSM332400, GSM332296, GSM332740, GSM331823, GSM332498, GSM332289, GSM331742, GSM331911, GSM332030, GSM332270, GSM332835, GSM332344, GSM332431, GSM332884, GSM332033, GSM332107, GSM332685, GSM332229, GSM331829, GSM332601, GSM332853, GSM332306, GSM331794, GSM332859, GSM332347, GSM331888, GSM332537, GSM332329, GSM331815, GSM332665, GSM332087, GSM332831, GSM332394, GSM332083, GSM332677, GSM332089, GSM332728, GSM332778, GSM332630, GSM332346, GSM332319, GSM331961, GSM331856, GSM331851, GSM331775, GSM332034, GSM331834, GSM332736, GSM332753, GSM332403, GSM331748, GSM331991, GSM332207, GSM332579, GSM332733, GSM332739, GSM332633, GSM332469, GSM331845, GSM332125, GSM332339, GSM332872, GSM331799, GSM332245, GSM331862, GSM332486, GSM332235, GSM332750, GSM332717, GSM332118, GSM332787, GSM332476, GSM331747, GSM332374, GSM332407, GSM332842, GSM332305, GSM332265, GSM331825, GSM332540, GSM332483 | C1, C2, C3, C4, C5, C8, C11, C13, C14, C15, C16, C17, C18 |

Table 64: Modules of cell samples of Microarray Innovations in Leukemia identified by $\mathcal{M}$-3

| Community | Samples | Cell type |
|---|---|---|
| 3 | GSM331737, GSM331850, GSM332119, GSM332318, GSM332628, GSM332549, GSM332137, GSM332179, GSM332761, GSM332130, GSM332870, GSM332762, GSM332806, GSM332162, GSM332641, GSM332214, GSM332274, GSM332690, GSM332672, GSM332260, GSM332450, GSM332576, GSM332213, GSM331804, GSM332440, GSM332416, GSM331908, GSM332333, GSM331905, GSM332480, GSM331886, GSM332741, GSM332474, GSM332613, GSM331820, GSM332478, GSM331802, GSM332515, GSM331998, GSM332155, GSM332362, GSM332707, GSM332862, GSM332363, GSM332156, GSM332167, GSM332783, GSM332781, GSM332819, GSM332104, GSM331746, GSM332108, GSM332670, GSM332232, GSM332342, GSM331858, GSM332003, GSM332618, GSM332060, GSM332390, GSM332465, GSM332854, GSM332472, GSM332338, GSM332398, GSM332635, GSM332794, GSM331871, GSM331910, GSM332386, GSM331887, GSM331904, GSM332626, GSM332200, GSM332494, GSM332427, GSM331985, GSM332356, GSM332058, GSM331882, GSM331885, GSM332878, GSM331864, GSM332744, GSM331819, GSM331863, GSM332364, GSM332570, GSM332415, GSM332590, GSM332051, GSM332267, GSM332017, GSM331895, GSM332027, GSM332571, GSM332243, GSM332874, GSM332380, GSM331995, GSM332298, GSM332587, GSM332662, GSM332140, GSM332542, GSM331751, GSM332302, GSM331790, GSM332097, GSM332805, GSM332355, GSM332754, GSM332488, GSM332780, GSM332814, GSM332843, GSM332517, GSM332075, GSM332751, GSM332065, GSM332015, GSM332006, GSM332588, GSM332495, GSM332147, GSM331932, GSM332018, GSM331933, GSM332482, GSM332144, GSM332803, GSM332484, GSM332435, GSM332328, GSM331958, GSM332228, GSM332691, GSM331971, GSM332254, GSM332393, GSM332131, GSM331803, GSM332101, GSM332709, GSM332174, GSM332128, GSM332238, GSM332086, GSM332565, GSM332620, GSM332124, GSM331941, GSM332010, GSM332792, GSM332777, GSM332150, GSM331989, GSM331770, GSM332461, GSM331981, GSM331931, GSM332546, GSM331791, GSM332098, GSM332668, GSM332149, GSM332351, GSM332382, GSM332138, GSM332852, GSM332833, GSM332530, GSM332244, GSM331881, GSM332647, GSM332767, GSM331875, GSM332071, GSM332802, GSM331832, GSM332703, GSM332774, GSM332688, GSM331745, GSM332120, GSM332873, GSM332047, GSM332275, GSM331839, GSM332401, GSM332880, GSM332650, GSM332317, GSM331853, GSM332838, GSM332110, GSM332704, GSM332492, GSM332443, GSM331785, | C4, C9, C11, C12, C13, C14, C17 |

Table 65: Modules of cell samples of Microarray Innovations in Leukemia identified by $\mathcal{M}$-4

| Community | Samples | Cell type |
|---|---|---|
| 3 | GSM331837, GSM332188, GSM331787, GSM331955, GSM332782, GSM332696, GSM332163, GSM332379, GSM332385, GSM331897, GSM332726, GSM332409, GSM332863, GSM332241, GSM332598, GSM331880, GSM332651, GSM331797, GSM332395, GSM332377, GSM332202, GSM332345, GSM331765, GSM332314, GSM332616, GSM332684, GSM332366, GSM332535, GSM331948, GSM331921, GSM331988, GSM332277, GSM332871, GSM331795, GSM332743, GSM332038, GSM332437, GSM332212, GSM332424, GSM332621, GSM332095, GSM332196, GSM331793, GSM331928, GSM332656, GSM331776 | C4, C9, C11, C12, C13, C14, C17 |
| 4 | GSM331739, GSM331741, GSM331951, GSM332008, GSM332466, GSM332094, GSM332644, GSM332304, GSM332680, GSM332388, GSM332288, GSM332025, GSM332881, GSM332582, GSM332569, GSM332815, GSM331996, GSM332299, GSM331997, GSM332522, GSM332493, GSM332029, GSM332449, GSM332745, GSM332839, GSM332834, GSM332772, GSM332365, GSM332438, GSM332133, GSM332152, GSM331821, GSM332059, GSM332528, GSM331969, GSM332747, GSM332462, GSM332426, GSM331927, GSM331870, GSM332479, GSM332036, GSM331817, GSM331757, GSM332746, GSM331774, GSM332776, GSM332105, GSM332266, GSM332816, GSM332447, GSM332640, GSM332801, GSM332151, GSM332168, GSM332308, GSM332109, GSM332442, GSM332370, GSM332141, GSM332487, GSM332768, GSM332135, GSM332785, GSM332321, GSM332538, GSM332387, GSM332216, GSM331959, GSM332643, GSM331982, | C4, C5, C7, C8 |

Table 66: Modules of cell samples of Microarray Innovations in Leukemia identified by $\mathcal{M}$-5

| Community | Samples | Cell type |
|---|---|---|
| 5 | GSM331740, GSM331743, GSM331759, GSM331773, GSM331783, GSM331854, GSM331983, GSM332597, GSM332421, GSM332475, GSM332091, GSM332181, GSM332453, GSM332612, GSM331809, GSM332420, GSM332875, GSM332622, GSM332158, GSM332865, GSM332014, GSM332764, GSM332539, GSM332273, GSM332341, GSM331978, GSM331972, GSM332856, GSM332558, GSM331814, GSM331876, GSM332473, GSM332191, GSM332586, GSM332171, GSM332234, GSM332077, GSM332166, GSM332606, GSM332081, GSM332165, GSM332879, GSM331917, GSM332820, GSM331906, GSM332223, GSM332276, GSM332331, GSM332303, GSM332541, GSM331761, GSM332253, GSM332655, GSM332756, GSM332250, GSM332414, GSM332595, GSM332766, GSM332563, GSM332327, GSM332016, GSM332793, GSM332257, GSM332026, GSM331889, GSM332531, GSM332381, GSM332076, GSM332825, GSM331805, GSM331973, GSM332659, GSM332845, GSM332594, GSM332049, GSM332708, GSM332503, GSM331976, GSM332178, GSM332625, GSM331970, GSM332309, GSM331808, GSM332069, GSM332564, GSM331806, GSM331930, GSM332092, GSM332172, GSM332636, GSM331987, GSM332405, GSM332840, GSM331913, GSM332769, GSM332093, GSM332727, GSM331947, GSM331873, GSM332221, GSM332661, GSM331843, GSM332470, GSM332194, GSM331768, GSM332336, GSM332721, GSM332352, GSM332082, GSM331767, GSM332208, GSM332300, GSM332391, GSM332514, GSM331929, GSM332070, GSM332648, GSM332127, GSM332048, GSM332869, GSM332023, GSM332354, GSM332021, GSM332596, GSM332425, GSM332857, GSM331915, GSM332507, GSM332674, GSM331902, GSM332673, GSM332784, GSM332883, GSM332559, GSM332176, GSM332320, GSM331937, GSM332074, GSM332718, GSM332159, GSM332567, GSM332556, GSM332189, GSM332002, GSM332043, GSM332810, GSM332657, GSM332226, GSM331957, GSM332512, GSM332287, GSM332290, GSM332779, GSM332864, GSM332589, GSM332520, GSM332201, GSM332607, GSM332169, GSM332562, GSM332526, GSM332510, GSM332752, GSM331811, GSM332765, GSM332064, GSM332186, GSM332161, GSM331762, GSM332434, GSM332117, GSM332114, GSM332121, GSM332037, GSM332689, GSM332524, GSM331874, GSM331816, GSM332078, GSM332600, GSM331893, GSM331977, GSM332770, GSM332692, GSM332795, GSM332432, GSM332578, GSM332090, GSM331778, GSM332547, | C4, C15, C17, C18 |

Table 67: Modules of cell samples of Microarray Innovations in Leukemia identified by $\mathcal{M}$-6

| Community | Samples | Cell type |
|---|---|---|
| 5 | GSM332671, GSM332315, GSM332735, GSM332584, GSM332233, GSM331868, GSM332022, GSM332430, GSM332007, GSM331752, GSM331994, GSM331860, GSM332040, GSM332122, GSM331867, GSM331944, GSM332451, GSM331934, GSM332642, GSM331963, GSM332020, GSM332217, GSM332729, GSM332375, GSM332533, GSM331946, GSM331950, GSM331827, GSM332408, GSM332527, GSM332581, GSM332184, GSM332170, GSM332552, GSM332011, GSM332711, GSM332046, GSM332324, GSM332367, GSM332322, GSM332028, GSM332413, GSM332551, GSM332497, GSM332749, GSM332701, GSM332206, GSM332183 | C4, C15, C17, C18 |
| 6 | GSM331782, GSM332035, GSM332463, GSM332534, GSM332129, GSM332521, GSM332258, GSM331926, GSM332251, GSM332664, GSM332882, GSM332808, GSM332705, GSM331952, GSM331975, GSM331789, GSM332824, GSM331807, GSM332809, GSM332585, GSM332456 | C10, C13 |
| 7 | GSM331786, GSM331918, GSM332013, GSM331993, GSM332639, GSM332554, GSM332136, GSM332759, GSM331964, GSM332723, GSM332786, GSM332293, GSM332577, GSM331968, GSM332192, GSM331894, GSM332205, GSM332811, GSM332738, GSM332291, GSM332240, GSM332052, GSM331831 | C2, C8 |
| 8 | GSM331830, GSM332053, GSM332055, GSM332249, GSM332282, GSM332441, GSM332422, GSM332371, GSM332460, GSM332139, GSM332264, GSM331974, GSM332032, GSM331866, GSM332516, GSM332182, GSM332412 | C11 |
| 9 | GSM331836, GSM331869, GSM331883, GSM332062, GSM332828, GSM332160, GSM332102, GSM332280, GSM332868, GSM332142, GSM332134, GSM332663, GSM332725, GSM332518, GSM332242, GSM332256, GSM332132, GSM332789, GSM332623, GSM332860, GSM332199, GSM332106, GSM332404, GSM332675, GSM332867, GSM332383, GSM332419, GSM332286, GSM332632, GSM331943, GSM332669, GSM332737, GSM331877, GSM331838, GSM332666, GSM332099, GSM332758, GSM332000, GSM331859, GSM331986, GSM331852 | C16, C17, C18 |

Table 68: Modules of cell samples of Microarray Innovations in Leukemia identified by $\mathcal{M}$-7

### Types found by $\mathcal{I}$

Tables 69 - 75 describe the modules found by $\mathcal{I}$.

| Community | Samples | Cell type |
|---|---|---|
| 1 | GSM332044, GSM332511, GSM332225, GSM332509, GSM332271, GSM332682, GSM331940, GSM332496, GSM332148, GSM332832, GSM332080, GSM332248, GSM332536, GSM332360, GSM332572, GSM332325, GSM332294, GSM331857, GSM331923, GSM331949, GSM331922, GSM332247, GSM332009, GSM332876, GSM331890, GSM332270, GSM331911, GSM331912, GSM332775, GSM332619, GSM331879, GSM332481, GSM332107, GSM332246, GSM332693, GSM332063, GSM331758, GSM332066, GSM332477, GSM332311, GSM332529, GSM332429, GSM332835, GSM332349, GSM332813, GSM332844, GSM332197, GSM331813, GSM331992, GSM332448, GSM332679, GSM331909, GSM331855, GSM331738, GSM332344, GSM332056, GSM332417, GSM332330, GSM332676, GSM331924, GSM332788, GSM332030, GSM332742, GSM332506, GSM331953, GSM332209, GSM332560, GSM332732, GSM332227, GSM331824, GSM332068, GSM331936, GSM331772, GSM332402, GSM331919, GSM332822, GSM331925, GSM331812, GSM331840, GSM332599, GSM332812, GSM331815, GSM332431, GSM332771, GSM332410, GSM332791, GSM331935, GSM332695, GSM332490, GSM332050, GSM332283, GSM332211, GSM332519, GSM332445, GSM332295, GSM332631, GSM332061, GSM332284, GSM332848, GSM332685, GSM331865 | C4, C8, C11, C13, C16, C17, C18 |
| 2 | GSM332352, GSM331947, GSM331843, GSM332470, GSM332093, GSM331767, GSM332166, GSM331761, GSM332354, GSM332661, GSM332276, GSM332531, GSM332300, GSM332514, GSM332857, GSM332879, GSM332208, GSM332793, GSM332023, GSM332596, GSM332405, GSM332016, GSM331913, GSM332026, GSM332327, GSM332673, GSM332648, GSM331915, GSM332082, GSM332563, GSM332336, GSM332657, GSM331906, GSM332043, GSM332512, GSM332507, GSM331873, GSM332226, GSM332766, GSM332414, GSM332756, GSM332076, GSM332320, GSM332021, GSM332194, GSM332721, GSM331929, GSM332784, GSM332655, GSM332287, GSM332718, GSM332223, GSM331957, GSM332425, GSM332221, GSM331902, GSM332869, GSM332810, GSM332559, GSM332381, GSM332257, GSM331768, GSM332074, GSM332595, GSM332250, GSM331889, GSM332674, GSM332391, GSM331937, GSM332883, GSM332048, GSM332159, GSM332290, GSM332779, GSM332176, GSM332864 | C15 |

Table 69: Modules of cell samples of Microarray Innovations in Leukemia identified by $\mathcal{I}$-1

| Community | Samples | Cell type |
|---|---|---|
| 3 | GSM332770, GSM331893, GSM332434, GSM332735, GSM331946, GSM332547, GSM332375, GSM331874, GSM332011, GSM332007, GSM332122, GSM332527, GSM332184, GSM332432, GSM332114, GSM332524, GSM331740, GSM332552, GSM332408, GSM332578, GSM332117, GSM331867, GSM332040, GSM332037, GSM332584, GSM332233, GSM331752, GSM332217, GSM332367, GSM331977, GSM332020, GSM332451, GSM332642, GSM332078, GSM331778, GSM331944, GSM332692, GSM332795, GSM332022, GSM332689, GSM332413, GSM331860, GSM332749, GSM332497, GSM332028, GSM331994, GSM332581, GSM331816, GSM332711, GSM332315, GSM332206, GSM332121, GSM332170, GSM331963, GSM332324, GSM331950, GSM332046, GSM331827, GSM332701, GSM332183, GSM332600, GSM332551, GSM332322, GSM331934, GSM332430, GSM331868, GSM332533, GSM332671, GSM332090, GSM332729 | C15 |
| 4 | GSM332710, GSM332722, GSM332683, GSM332720, GSM332255, GSM332543, GSM331872, GSM332239, GSM332796, GSM332561, GSM332154, GSM331956, GSM332307, GSM332583, GSM332343, GSM332702, GSM331844, GSM332084, GSM332146, GSM332113, GSM332858, GSM331792, GSM332760, GSM332734, GSM332100, GSM332116, GSM331788, GSM331849, GSM331960, GSM332005, GSM332057, GSM332557, GSM331920, GSM331907, GSM332436, GSM332088, GSM332326, GSM331828, GSM331980, GSM332712, GSM332175, GSM332575, GSM332332, GSM332748, GSM332468, GSM332215, GSM332039, GSM332369, GSM332471, GSM332850, GSM332204, GSM332804, GSM332045, GSM332730, GSM332464, GSM332433, GSM332153, GSM332454, GSM332222, GSM331771, GSM331800, GSM332373, GSM332706, GSM332724, GSM331736, GSM332610, GSM332237, GSM332263, GSM332334, GSM332185, GSM332112, GSM332502, GSM332513, GSM332072, GSM332459, GSM332694, GSM331763, GSM332847, GSM332444 | C2, C3, C7, C8 |

Table 70: Modules of cell samples of Microarray Innovations in Leukemia identified by $\mathcal{I}$-2

| Community | Samples | Cell type |
|---|---|---|
| 5 | GSM332388, GSM332304, GSM331996, GSM332522, GSM332438, GSM331951, GSM332815, GSM332299, GSM332449, GSM332569, GSM332493, GSM332029, GSM332881, GSM332308, GSM332801, GSM332152, GSM332644, GSM332133, GSM332768, GSM332680, GSM332834, GSM332582, GSM332487, GSM332426, GSM332447, GSM332288, GSM332151, GSM332839, GSM332109, GSM332745, GSM332370, GSM331821, GSM332772, GSM331969, GSM331959, GSM332387, GSM332442, GSM332776, GSM332528, GSM332036, GSM331757, GSM332538, GSM332059, GSM332462, GSM332135, GSM331997, GSM332479, GSM332321, GSM332105, GSM332266, GSM332168, GSM331739, GSM332747, GSM331774, GSM332141, GSM332746, GSM332094, GSM331982, GSM332025, GSM331870, GSM331741, GSM332216, GSM332466, GSM332643, GSM331817, GSM332640, GSM332365, GSM332008, GSM332816, GSM332785, GSM331927 | C4, C5, C7, C8 |
| 6 | GSM332716, GSM332218, GSM332335, GSM332715, GSM332312, GSM331965, GSM332714, GSM332190, GSM331891, GSM332719, GSM332262, GSM332337, GSM332085, GSM332855, GSM332798, GSM332428, GSM331990, GSM332001, GSM332757, GSM332697, GSM331896, GSM332180, GSM332411, GSM332797, GSM332817, GSM332593, GSM332157, GSM332281, GSM331999, GSM331781, GSM332054, GSM332340, GSM332458, GSM331780, GSM332359, GSM332504, GSM332654, GSM331826, GSM332224, GSM331766, GSM332821, GSM332608, GSM332143, GSM332145, GSM331884, GSM331966, GSM332818, GSM332198, GSM332755, GSM331796, GSM332210, GSM332841, GSM332591, GSM332646, GSM332713, GSM331754, GSM331967, GSM331901, GSM332568, GSM332773, GSM332193, GSM331892, GSM331756, GSM332004, GSM331898, GSM331733 | C3, C4, C5, C7, C8, C13 |
| 7 | GSM332861, GSM331835, GSM332544, GSM332378, GSM332446, GSM331903, GSM332807, GSM331841, GSM332323, GSM332372, GSM331962, GSM332485, GSM332220, GSM332823, GSM332164, GSM331801, GSM332686, GSM331861, GSM331979, GSM332501, GSM332836, GSM332615, GSM332491, GSM332310, GSM332259, GSM332103, GSM332700, GSM332439, GSM332042, GSM331742, GSM332418, GSM332851, GSM332394, GSM331769, GSM332731, GSM332348, GSM332609, GSM331779, GSM332126, GSM332296, GSM332532, GSM332498, GSM332455, GSM332268, GSM332285, GSM332400, GSM332740, GSM332602, GSM332658, GSM332611, GSM331823, GSM332115, GSM332396, GSM331825, GSM332111, GSM332261, GSM332624, GSM332278, GSM332289, GSM331983 | C1, C4, C8, C13, C14, C15, C17, C18 |

Table 71: Modules of cell samples of Microarray Innovations in Leukemia identified by $\mathcal{I}$-3

| Community | Samples | Cell type |
|---|---|---|
| 8 | GSM332618, GSM331871, GSM331820, GSM332472, GSM332167, GSM332156, GSM332613, GSM332338, GSM331885, GSM331910, GSM332214, GSM332137, GSM332480, GSM332267, GSM332878, GSM332427, GSM332794, GSM332058, GSM331882, GSM332628, GSM332232, GSM332670, GSM332819, GSM332741, GSM331737, GSM332386, GSM332494, GSM332626, GSM331858, GSM332104, GSM332003, GSM332333, GSM331985, GSM331904, GSM332781, GSM331905, GSM331908, GSM331863, GSM331819, GSM331887, GSM332635, GSM332854, GSM332342, GSM332274, GSM332762, GSM331864, GSM332744, GSM332398, GSM332474, GSM332783, GSM331886, GSM332162, GSM332570, GSM332641, GSM332549, GSM332017, GSM332364, GSM332590, GSM332060, GSM332415, GSM332318, GSM332806, GSM332356, GSM332108, GSM332200, GSM332051, GSM331802, GSM332390, GSM332465, GSM331746, GSM332478, GSM331850, GSM332416, GSM332870 | C4, C12, C13 |
| 9 | GSM332802, GSM332843, GSM331989, GSM332149, GSM332530, GSM332726, GSM332651, GSM331832, GSM332393, GSM332833, GSM331875, GSM332482, GSM332244, GSM332138, GSM332668, GSM332852, GSM332098, GSM332792, GSM332688, GSM332163, GSM331981, GSM332780, GSM331791, GSM331897, GSM332461, GSM332385, GSM332777, GSM332703, GSM332546, GSM331745, GSM332650, GSM332241, GSM332120, GSM332774, GSM332150, GSM332188, GSM332696, GSM332484, GSM332863, GSM332409, GSM332071, GSM331787, GSM332620, GSM331770, GSM331955, GSM332873, GSM332047, GSM331880, GSM332492, GSM332379, GSM332351, GSM331839, GSM332275, GSM331797, GSM332782, GSM332616, GSM332767, GSM332382, GSM332443, GSM332144, GSM332880, GSM331931, GSM332401, GSM332647, | C12, C13 |
| 10 | GSM332357, GSM332231, GSM332406, GSM331777, GSM332545, GSM332219, GSM332653, GSM332079, GSM332592, GSM332604, GSM332573, GSM332681, GSM332031, GSM332678, GSM332353, GSM332500, GSM332553, GSM331939, GSM332540, GSM332376, GSM331916, GSM332173, GSM332195, GSM331847, GSM332467, GSM332041, GSM331842, GSM332236, GSM332499, GSM332667, GSM331735, GSM332799, GSM331900, GSM332687, GSM332837, GSM331822, GSM332555, GSM332800, GSM332012, GSM331899, GSM331942, GSM332846, GSM332790, GSM331760, GSM332763, GSM332252, GSM332368, GSM331784, GSM332827, GSM332177, GSM332523, GSM331914, GSM332073, GSM332525, GSM331798, | C3, C5, C8 |

Table 72: Modules of cell samples of Microarray Innovations in Leukemia identified by $\mathcal{I}$-4

| Community | Samples | Cell type |
|---|---|---|
| 11 | GSM332256, GSM332134, GSM331836, GSM331883, GSM332725, GSM332518, GSM332868, GSM332280, GSM332623, GSM331943, GSM332669, GSM332242, GSM332132, GSM331869, GSM332663, GSM331852, GSM332102, GSM332142, GSM332632, GSM332737, GSM332860, GSM332305, GSM332355, GSM331877, GSM332867, GSM332099, GSM331838, GSM332106, GSM332419, GSM332404, GSM332160, GSM332666, GSM332034, GSM331986, GSM332286, GSM332675, GSM332828, GSM332383, GSM332000, GSM332789, GSM332265, GSM331859, GSM332199, GSM332062, GSM332758, GSM332483 | C1, C13, C16, C17, C18 |
| 12 | GSM332539, GSM332453, GSM331809, GSM332475, GSM332191, GSM332181, GSM332091, GSM332558, GSM332273, GSM332077, GSM331814, GSM332612, GSM332421, GSM332856, GSM332473, GSM332420, GSM332014, GSM332764, GSM332165, GSM332303, GSM331773, GSM332171, GSM332865, GSM332606, GSM332820, GSM332597, GSM332234, GSM331876, GSM332586, GSM332875, GSM332158, GSM332253, GSM331972, GSM332081, GSM331759, GSM332341, GSM332622, GSM331743, GSM331854, | C15 C17 |
| 13 | GSM332826, GSM331748, GSM332739, GSM331845, GSM332736, GSM332614, GSM332469, GSM331799, GSM332339, GSM332733, GSM331750, GSM332033, GSM332550, GSM332872, GSM332579, GSM332717, GSM331834, GSM332245, GSM332407, GSM332235, GSM332486, GSM331862, GSM332505, GSM331991, GSM332633, GSM332187, GSM332753, GSM331747, GSM332207, GSM331734, GSM332750, GSM332125, GSM332787, GSM332842, GSM332403, GSM332374, GSM332118, GSM332830, GSM332476 | C2, C4, C5, C8, C11, C13, C16, C17, C18 |
| 14 | GSM332659, GSM331978, GSM332049, GSM332309, GSM332331, GSM331917, GSM332092, GSM331973, GSM331930, GSM332825, GSM331806, GSM332708, GSM332625, GSM332541, GSM332594, GSM331783, GSM332845, GSM332727, GSM331805, GSM331970, GSM332636, GSM332172, GSM332564, GSM332769, GSM332178, GSM332840, GSM332064, GSM332503, GSM332567, GSM331976, GSM331987, GSM332069, GSM331808 | C15 |
| 15 | GSM332229, GSM332228, GSM332292, GSM332665, GSM332089, GSM332435, GSM332174, GSM332566, GSM331933, GSM332831, GSM332346, GSM332306, GSM332328, GSM332086, GSM332853, GSM332329, GSM331837, GSM332601, GSM332677, GSM332101, GSM332565, GSM332238, GSM331958, GSM331794, GSM332110, GSM332838, GSM332083, GSM332709, GSM332537, GSM332691, GSM331829, GSM332087, GSM331888, GSM332131, GSM332254, GSM331803 | C13, C14, C17, |

Table 73: Modules of cell samples of Microarray Innovations in Leukemia identified by $\mathcal{I}$-5

| Community | Samples | Cell type |
|---|---|---|
| 16 | GSM332580, GSM331755, GSM332452, GSM332279, GSM331833, GSM332301, GSM331945, GSM332096, GSM331848, GSM332019, GSM331810, GSM332574, GSM332423, GSM332361, GSM332384, GSM331749, GSM332603, GSM331744, GSM332350, GSM331938, GSM331984, GSM332866, GSM332392, GSM331753, GSM331764, GSM332399, GSM332637, GSM332829, GSM332203, GSM332660, GSM332489, GSM332649, GSM332508, GSM332316 | C5, C8 |
| 17 | GSM332202, GSM331765, GSM332684, GSM332395, GSM331948, GSM332038, GSM331881, GSM331775, GSM332345, GSM331988, GSM332212, GSM332437, GSM332314, GSM332535, GSM331921, GSM332743, GSM332196, GSM332366, GSM332095, GSM331795, GSM332277, GSM331776, GSM332656, GSM331928, GSM331793, GSM332621, GSM332424, GSM332377 | C12, C13, C17 |
| 18 | GSM331961, GSM332884, GSM332859, GSM332630, GSM331853, GSM332704, GSM331941, GSM332803, GSM332605, GSM332024, GSM332347, GSM332778, GSM332728, GSM332319, GSM332010, GSM331785, GSM332598, GSM332272, GSM331856, GSM332317, GSM331851 | C13, C17 |
| 19 | GSM332786, GSM332136, GSM332554, GSM332723, GSM331964, GSM332639, GSM331993, GSM331786, GSM332240, GSM332811, GSM332759, GSM332205, GSM332577, GSM332291, GSM331894, GSM332052, GSM332293, GSM332738, GSM332192, GSM331968, GSM331831, GSM332013, GSM331918 | C2, C8 |
| 20 | GSM332124, GSM332139, GSM332055, GSM332460, GSM332371, GSM332249, GSM332422, GSM332264, GSM332412, GSM331974, GSM331866, GSM332128, GSM331971, GSM332871, GSM331830, GSM332182, GSM332282, GSM332516, GSM332053, GSM332032, GSM332441 | C11, C13 |
| 21 | GSM332169, GSM332607, GSM332556, GSM332070, GSM332520, GSM332201, GSM332002, GSM332189, GSM332562, GSM332161, GSM332526, GSM332752, GSM331762, GSM332510, GSM332765, GSM332589, GSM332186, GSM332127, GSM331811, | C15, C18 |
| 22 | GSM332251, GSM331926, GSM332258, GSM332521, GSM332808, GSM332129, GSM331789, GSM331975, GSM332882, GSM332664, GSM331952, GSM332705, GSM332824, GSM332534, GSM332456, GSM332463, GSM332809, GSM331807, GSM332585, GSM331782, GSM332035, | C10, C13 |
| 23 | GSM332450, GSM332707, GSM332515, GSM331804, GSM332260, GSM332363, GSM332862, GSM332155, GSM332690, GSM332362, GSM332213, GSM332576, GSM332130, GSM332440, GSM332761, GSM331998, GSM332672, GSM332179, GSM332119, | C4, C13, C14 |

Table 74: Modules of cell samples of Microarray Innovations in Leukemia identified by $\mathcal{I}$-6

| Community | Samples | Cell type |
|---|---|---|
| 24 | GSM332123, GSM331878, GSM332699, GSM332629, GSM332230, GSM332457, GSM332067, GSM332297, GSM331818, GSM332638, GSM332269, GSM332313, GSM332652, GSM332634, GSM331846, GSM332877, GSM332645, GSM332548, GSM332617, | C6, C8 |
| 25 | GSM332243, GSM332380, GSM332662, GSM332874, GSM332542, GSM332587, GSM332027, GSM332805, GSM331751, GSM332571, GSM332302, GSM331790, GSM332298, GSM332754, GSM332140, GSM331995, GSM332097, | C9, C13 |
| 26 | GSM332517, GSM332495, GSM332015, GSM332018, GSM332488, GSM332065, GSM332751, GSM332814, GSM331932, GSM331895, GSM332006, GSM332588, GSM332147, GSM332075, | C12, C13, C14 |
| 27 | GSM332627, GSM332698, GSM332397, GSM331954, GSM332358, GSM332849, GSM332389, | C13, C14, C16 |

Table 75: Modules of cell samples of Microarray Innovations in Leukemia identified by $\mathcal{I}$-7

## Types found by $\mathcal{E}^2$

Tables 76 to 83 describe the modules of the new test leukemia data found by $\mathcal{E}^2$.

| Community | Samples | Cell type |
|---|---|---|
| 1 | GSM331733, GSM331891, GSM332198, GSM332568, GSM332591, GSM332359, GSM332798, GSM332281, GSM332646, GSM332262, GSM332210, GSM332716, GSM332818, GSM332157, GSM332312, GSM331965, GSM332218, GSM332143, GSM332145, GSM332224, GSM332797, GSM331780, GSM332504, GSM331756, GSM332085, GSM332715, GSM332593, GSM332180, GSM332193, GSM331967, GSM331999, GSM331781, GSM332411, GSM331884, GSM331990, GSM332337, GSM332054, GSM332458, GSM332719, GSM332713, GSM332697, GSM332428, GSM332817, GSM332773, GSM331966, GSM332714, GSM331796, GSM331896, GSM331826, GSM332001, GSM332004, GSM331766, GSM332755, GSM332841, GSM331892, GSM331754, GSM331901, GSM332654, GSM332608 | C3, C4, C5, C7, C8, C13 |
| 2 | GSM331734, GSM331799, GSM331835, GSM332403, GSM332633, GSM332778, GSM332739, GSM332733, GSM332872, GSM332469, GSM331845, GSM332339, GSM332717, GSM332245, GSM332235, GSM332579, GSM332486, GSM332750, GSM331862, GSM332033, GSM331991, GSM332787, GSM331742, GSM332372, GSM331962, GSM331750, GSM332187, GSM332476, GSM332550, GSM332836, GSM332310, GSM332378, GSM332125, GSM332700, GSM332830, GSM331747, GSM332296, GSM331823, GSM332740, GSM332289, GSM332400, GSM332498, GSM332126, GSM332544, GSM332285, GSM332272, GSM332118, GSM332848, GSM332115, GSM332611, GSM332418, GSM332396, GSM332278, GSM332698, GSM331825, GSM332397, GSM331861, GSM331954, GSM332849, GSM332627, GSM332358, GSM332389, GSM332609, GSM332292, GSM331841, GSM331979, GSM332615, GSM332532, GSM332111, GSM332602, GSM332164, GSM331779, GSM332731, GSM332485, GSM332624, GSM331983 | C1, C4, C5, C8, C11, C13, C14, C15, C16, C17, C18 |

Table 76: Modules of cell samples of Microarray Innovations in Leukemia identified by $\mathcal{E}^2$-1

| Community | Samples | Cell type |
|---|---|---|
| 3 | GSM331735, GSM331736, GSM331763, GSM331828, GSM332185, GSM332237, GSM332464, GSM332039, GSM332710, GSM332116, GSM332468, GSM332100, GSM331956, GSM332804, GSM332436, GSM332702, GSM332255, GSM332722, GSM331907, GSM332146, GSM332583, GSM332543, GSM331872, GSM331980, GSM332307, GSM332454, GSM332720, GSM332332, GSM331788, GSM332343, GSM332373, GSM332706, GSM332561, GSM331849, GSM332734, GSM332575, GSM331960, GSM332113, GSM332850, GSM332683, GSM332005, GSM332712, GSM332215, GSM332796, GSM331920, GSM332502, GSM332239, GSM332858, GSM332204, GSM331792, GSM332057, GSM332847, GSM332154, GSM332088, GSM332459, GSM332072, GSM332175, GSM332269, GSM332230, GSM332222, GSM332760, GSM332748, GSM331771, GSM332724, GSM332084, GSM332369, GSM332334, GSM331844, GSM332557, GSM332444, GSM332045, GSM332297, GSM332827, GSM331777, GSM332513, GSM332153, GSM331800, GSM332326, GSM332730, GSM332112, GSM332694, GSM331900, GSM332687, GSM332837, GSM332231, GSM332653, GSM332553, GSM332523, GSM332800, GSM332406, GSM331899, GSM332467, GSM331914, GSM332681, GSM332012, GSM332790, GSM331942, GSM332236, GSM332545, GSM332173, GSM332195, GSM332573, GSM332604, GSM331842, GSM332357, GSM332846, GSM332799, GSM331822, GSM332592, GSM332376, GSM332500, GSM332079, GSM332252, GSM332667, GSM332353, GSM331847, GSM331784, GSM332499, GSM332177, GSM332555, GSM332678, GSM331760, GSM332031, GSM332219, GSM331916, GSM332335, GSM332041, GSM332763, GSM331939, GSM332073, GSM332821, GSM332190, GSM332610, GSM332340, GSM332757, GSM332525, GSM332471, GSM332433, GSM332263, GSM332855, GSM331898, GSM331798, GSM332368, GSM332540 | C2, C3, C5, C7, C8 |

Table 77: Modules of cell samples of Microarray Innovations in Leukemia identified by $\mathcal{E}^2$-2

| Community | Samples | Cell type |
|---|---|---|
| 4 | GSM331737, GSM331746, GSM331819, GSM331864, GSM332415, GSM332878, GSM331910, GSM331871, GSM332338, GSM332472, GSM331863, GSM332570, GSM331887, GSM332386, GSM332618, GSM331882, GSM332794, GSM331885, GSM332364, GSM332744, GSM331820, GSM332058, GSM332635, GSM332854, GSM332274, GSM332398, GSM332427, GSM331904, GSM332356, GSM332003, GSM331858, GSM332060, GSM332670, GSM332167, GSM332342, GSM332232, GSM332156, GSM332783, GSM332390, GSM332741, GSM332465, GSM332819, GSM332781, GSM332104, GSM332613, GSM332626, GSM332200, GSM332108, GSM331985, GSM332494, GSM332480, GSM331802, GSM332051, GSM332590, GSM332267, GSM332017, GSM332214, GSM332478, GSM331886, GSM332474, GSM331905, GSM332333, GSM331908, GSM332137, GSM332162, GSM332628, GSM332416, GSM332762, GSM332318, GSM332870, GSM332806, GSM332549, GSM332641, GSM331850, GSM332761, GSM332119 | C4, C12, C13, C14 |
| 5 | GSM331738, GSM331748, GSM331772, GSM331790, GSM331836, GSM331869, GSM331883, GSM332286, GSM332383, GSM332419, GSM332867, GSM332623, GSM332134, GSM332242, GSM332518, GSM332789, GSM332860, GSM332725, GSM332675, GSM332132, GSM332868, GSM332280, GSM332256, GSM332828, GSM332102, GSM332632, GSM331943, GSM332669, GSM332199, GSM332663, GSM332106, GSM332404, GSM332737, GSM331877, GSM332062, GSM332160, GSM332142, GSM331838, GSM332666, GSM332099, GSM332805, GSM332000, GSM332758, GSM331859, GSM331986, GSM332572, GSM331909, GSM332483, GSM332305, GSM332063, GSM332034, GSM332407, GSM332374, GSM332265, GSM331852, GSM332355, GSM331857, GSM332044, GSM332537, GSM332529, GSM332536, GSM332227, GSM332330, GSM331940, GSM332813, GSM332511, GSM332225, GSM332496, GSM332742, GSM332736, GSM332207, GSM332753, GSM331834, GSM332080, GSM332211, GSM332448, GSM332445, GSM332009, GSM332259, GSM332614, GSM332283, GSM332826, GSM332693, GSM332107, GSM332505, | C1, C4, C8, C9, C11, C13, C16, C17, C18 |

Table 78: Modules of cell samples of Microarray Innovations in Leukemia identified by $\mathcal{E}^2$-3

| Community | Samples | Cell type |
|---|---|---|
| 5 | GSM332506, GSM332402, GSM331953, GSM332832, GSM332042, GSM332861, GSM332519, GSM332349, GSM331801, GSM332344, GSM332270, GSM332812, GSM332220, GSM332807, GSM332148, GSM332676, GSM332271, GSM331840, GSM332311, GSM332050, GSM331924, GSM332061, GSM331949, GSM332446, GSM332509, GSM332791, GSM332876, GSM332619, GSM332410, GSM332788, GSM332360, GSM331879, GSM331919, GSM332481, GSM332248, GSM332209, GSM331936, GSM331890, GSM331855, GSM332682, GSM332429, GSM331912, GSM331813, GSM332560, GSM332775, GSM332325, GSM331923, GSM332066, GSM332822, GSM332294, GSM332835, GSM331911, GSM332695, GSM332417, GSM332631, GSM331925, GSM332197, GSM331922, GSM332030, GSM332771, GSM332247, GSM331935, GSM331992, GSM332295, GSM332246, GSM331824, GSM332490, GSM332599, GSM332477, GSM332679, GSM331758, GSM332732, GSM332844, GSM332068, GSM331865, GSM332056, GSM332431, GSM332601, GSM332491, GSM332261, GSM332686, GSM331812, GSM332103, GSM332455, GSM332323, GSM331903, GSM332439, GSM332658, GSM332823, GSM332501, GSM332268, GSM331769, GSM332284, GSM332685 | C1, C4, C8, C9, C11, C13, C16, C17, C18 |
| 6 | GSM331739, GSM331741, GSM331951, GSM332094, GSM332466, GSM332321, GSM332538, GSM332487, GSM332288, GSM331959, GSM332308, GSM332582, GSM332569, GSM332815, GSM332801, GSM332442, GSM332772, GSM332387, GSM332216, GSM332135, GSM332109, GSM332141, GSM332370, GSM332151, GSM332785, GSM332768, GSM332449, GSM332447, GSM332388, GSM332304, GSM332834, GSM332881, GSM331996, GSM332493, GSM332299, GSM332680, GSM332168, GSM332029, GSM332640, GSM331997, GSM332839, GSM332365, GSM332059, GSM332745, GSM332025, GSM332528, GSM332522, GSM332438, GSM331821, GSM332644, GSM331969, GSM332747, GSM332152, GSM332426, GSM332462, GSM332133, GSM331927, GSM331870, GSM332479, GSM332036, GSM332266, GSM331817, GSM332105, GSM332776, GSM331757, GSM331774, GSM332746, GSM332816, GSM332008, GSM332643 | C4, C5, C7, C8 |

Table 79: Modules of cell samples of Microarray Innovations in Leukemia identified by $\mathcal{E}^2$-4

| Community | Samples | Cell type |
|---|---|---|
| 7 | GSM331740, GSM331762, GSM331767, GSM331843, GSM331957, GSM332567, GSM332226, GSM332043, GSM332661, GSM332512, GSM332657, GSM332287, GSM331977, GSM332300, GSM332405, GSM331947, GSM332082, GSM332470, GSM332336, GSM332208, GSM332352, GSM332721, GSM331913, GSM332659, GSM332093, GSM332194, GSM331768, GSM332810, GSM331929, GSM332514, GSM332718, GSM332648, GSM332769, GSM331930, GSM332092, GSM332172, GSM332049, GSM332221, GSM331978, GSM332309, GSM331987, GSM331806, GSM332636, GSM331873, GSM332564, GSM331917, GSM332391, GSM332825, GSM332840, GSM332727, GSM332048, GSM332070, GSM332879, GSM332556, GSM331970, GSM331973, GSM331805, GSM332625, GSM332069, GSM331808, GSM332594, GSM332331, GSM332708, GSM332845, GSM332178, GSM332503, GSM331976, GSM331783, GSM332541, GSM332189, GSM332869, GSM332002, GSM332558, GSM332159, GSM332290, GSM332779, GSM332169, GSM332127, GSM332607, GSM332114, GSM332064, GSM332117, GSM332520, GSM332201, GSM332589, GSM332562, GSM331809, GSM332752, GSM332510, GSM332526, GSM332434, GSM332765, GSM331811, GSM332303, GSM332181, GSM332121, GSM332037, GSM332689, GSM332078, GSM331874, GSM332524, GSM332186, GSM332161, GSM331893, GSM331816, GSM332600, GSM332770, GSM332692, GSM332432, GSM332547, GSM332735, GSM332578, GSM331778, GSM332233, GSM332584, GSM331868, GSM332022, GSM332430, GSM332007, GSM332671, GSM332315, GSM332795, GSM331867, GSM331860, GSM331994, GSM331752, GSM332122, GSM332040, GSM332864, GSM332021, GSM332375, GSM332642, GSM332020, GSM332217, GSM331944, GSM331963, GSM332729, GSM332451, GSM331934, GSM332533, GSM331946, GSM331950, GSM332322, GSM332090, GSM332367, GSM332527, GSM332581, GSM332011, GSM332552, GSM332046, GSM332184, GSM332170, GSM331827, GSM332711, GSM332408, GSM332324, GSM332413, GSM332028, GSM332701, GSM332206, GSM332749, GSM332497, GSM332183, GSM332551 | C15, C18 |

Table 80: Modules of cell samples of Microarray Innovations in Leukemia identified by $\mathcal{E}^2$-5

| Community | Samples | Cell type |
|---|---|---|
| 8 | GSM331743, GSM331759, GSM332764, GSM332865, GSM332014, GSM332091, GSM332453, GSM332597, GSM332612, GSM332622, GSM332475, GSM332420, GSM332875, GSM332421, GSM332539, GSM332273, GSM332341, GSM332586, GSM331814, GSM332856, GSM331972, GSM331876, GSM332473, GSM332191, GSM332234, GSM332077, GSM332171, GSM332606, GSM332166, GSM332081, GSM332165, GSM332820, GSM331906, GSM332276, GSM332223, GSM331854, GSM331773, GSM332158, GSM331761, GSM332253, GSM332655, GSM332756, GSM332250, GSM332414, GSM332595, GSM332766, GSM332563, GSM332327, GSM332016, GSM332257, GSM332793, GSM332026, GSM331889, GSM332531, GSM332381, GSM332076, GSM332673, GSM331902, GSM332425, GSM332320, GSM332023, GSM332354, GSM331937, GSM332784, GSM332857, GSM332596, GSM331915, GSM332074, GSM332507, GSM332674, GSM332559, GSM332883, GSM332176 | C15, C17 |
| 9 | GSM331744, GSM331749, GSM332866, GSM332423, GSM332574, GSM331833, GSM332019, GSM332508, GSM332452, GSM332279, GSM331984, GSM331938, GSM332580, GSM332203, GSM332392, GSM331755, GSM332096, GSM332829, GSM332637, GSM332660, GSM332350, GSM332301, GSM331982, GSM332399, GSM331945, GSM332384, GSM332316, GSM331810, GSM331753, GSM332361, GSM331764, GSM331848, GSM332603, GSM332489, GSM332649 | C5, C8 |

Table 81: Modules of cell samples of Microarray Innovations in Leukemia identified by $\mathcal{E}^2$-6

| Community | Samples | Cell type |
|---|---|---|
| 10 | GSM331745, GSM331770, GSM331803, GSM331853, GSM331895, GSM331932, GSM332147, GSM332751, GSM332018, GSM332065, GSM332495, GSM332588, GSM332517, GSM332488, GSM332412, GSM332015, GSM332006, GSM332450, GSM332843, GSM332814, GSM332075, GSM332707, GSM332803, GSM332482, GSM332435, GSM332144, GSM331958, GSM332328, GSM332228, GSM331933, GSM331971, GSM332691, GSM332851, GSM332838, GSM332128, GSM332440, GSM332124, GSM332690, GSM332348, GSM332394, GSM332672, GSM332862, GSM331941, GSM332010, GSM332703, GSM332317, GSM332101, GSM332131, GSM332393, GSM332254, GSM332709, GSM332086, GSM332174, GSM332238, GSM332565, GSM332089, GSM332677, GSM332831, GSM332083, GSM332346, GSM332665, GSM332362, GSM332260, GSM332576, GSM332515, GSM332213, GSM332155, GSM331804, GSM332363, GSM331998, GSM332130, GSM332179, GSM332780, GSM332620, GSM331832, GSM332792, GSM331989, GSM332777, GSM332461, GSM332546, GSM331981, GSM332150, GSM332852, GSM331791, GSM331931, GSM332149, GSM332098, GSM332668, GSM332138, GSM332382, GSM332351, GSM332530, GSM332244, GSM332802, GSM332833, GSM331881, GSM331875, GSM332767, GSM332071, GSM332484, GSM332688, GSM332774, GSM332647, GSM332120, GSM332873, GSM332047, GSM331839, GSM332275, GSM332880, GSM332401, GSM332650, GSM332110, GSM332704, GSM331961, GSM331785, GSM332188, GSM331837, GSM332598, GSM332863, GSM331787, GSM332492, GSM332726, GSM332409, GSM332696, GSM332443, GSM331955, GSM332163, GSM332385, GSM331897, GSM332379, GSM332782, GSM332395, GSM332651, GSM331797, GSM331880, GSM332241, GSM332202, GSM332345, GSM331765, GSM332377, GSM332314, GSM332684, GSM332616, GSM331921, GSM331948, GSM332277, GSM331988, GSM332366, GSM332535, GSM332871, GSM332038, GSM331795, GSM332743, GSM332212, GSM332437, GSM331775, GSM332630, GSM332424, GSM331851 | C4, C11, C12, C13, C14, C17 |
| 11 | GSM331751, GSM332542, GSM332587, GSM332874, GSM332380, GSM332662, GSM332243, GSM332298, GSM331995, GSM332571, GSM332027, GSM332140, GSM332302, GSM332097, GSM332754 | C9, C13 |
| 12 | GSM331776, GSM331793, GSM332196, GSM332095, GSM332656, GSM331928, GSM332621 | C12 |

Table 82: Modules of cell samples of Microarray Innovations in Leukemia identified by $\mathcal{E}^2$-7

| Community | Samples | Cell type |
|---|---|---|
| 13 | GSM331782, GSM332463, GSM332534, GSM332035, GSM331926, GSM332521, GSM332251, GSM332129, GSM332258, GSM332882, GSM332664, GSM332808, GSM331952, GSM332705, GSM331975, GSM331789, GSM332824, GSM332809, GSM331807, GSM332585, GSM332456 | C10, C13 |
| 14 | GSM331786, GSM331964, GSM331968, GSM332192, GSM332759, GSM332577, GSM332723, GSM332639, GSM331993, GSM332136, GSM332554, GSM332786, GSM331894, GSM332205, GSM332738, GSM332811, GSM331831, GSM332291, GSM331918, GSM332052, GSM332293, GSM332240, GSM332013, GSM332842 | C2, C8 |
| 15 | GSM331794, GSM331815, GSM332087, GSM332329, GSM332859, GSM332728, GSM332229, GSM332319, GSM332347, GSM331856, GSM332884, GSM332605, GSM332306, GSM331829, GSM331888, GSM332853, GSM332024, GSM332566 | C13, C14, C17 |
| 16 | GSM331818, GSM331846, GSM332638, GSM332313, GSM332877, GSM331878, GSM332067, GSM332652, GSM332699, GSM332123, GSM332629, GSM332457, GSM332634, GSM332617, GSM332548, GSM332645 | C6, C8 |
| 17 | GSM331830, GSM331866, GSM331974, GSM332032, GSM332516, GSM332422, GSM332460, GSM332055, GSM332139, GSM332371, GSM332249, GSM332441, GSM332182, GSM332264, GSM332282, GSM332053 | C11 |

Table 83: Modules of cell samples of Microarray Innovations in Leukemia identified by $\mathcal{E}^2$-8

### Types found by $\mathcal{E}^3$

Tables 84 to 91 describe the modules of the new test leukemia data found by $\mathcal{E}^3$.

| First level | Second level | Cell type |
|---|---|---|
| 1 | GSM331733, GSM332716, GSM332312, GSM332145, GSM331780, GSM332504, GSM332411, GSM332337, GSM331781, GSM332143, GSM332593, GSM331990, GSM331966, GSM331796, GSM331896, GSM332714, GSM332004 | |
| | GSM331891, GSM332198, GSM332568, GSM332591, GSM332359, GSM332281, GSM332798, GSM332646, GSM332210, GSM332262, GSM331756, GSM332818, GSM332157, GSM332224, GSM332085, GSM332193 | |
| | GSM331965, GSM331826, GSM331766, GSM331754, GSM332654, GSM331901 | |
| | GSM332218, GSM332715, GSM331884, GSM332458, GSM332054, GSM332428, GSM332773, GSM332697, GSM332817, GSM332719, GSM331999, GSM332180, GSM331892, GSM332713, GSM332797, GSM331967 | |
| | GSM332001, GSM332755, GSM332841, GSM332608 | |
| 2 | GSM331734, GSM331747, GSM332550 | |
| | GSM331799, GSM332403, GSM332633, GSM332778, GSM332739, GSM332733, GSM331835, GSM332787, GSM332717, GSM332235, GSM332872, GSM331845, GSM332339, GSM332469, GSM332245, GSM332486, GSM332579, GSM332750, GSM331862, GSM331742, GSM332118 | |
| | GSM332033, GSM331991, GSM331750, GSM332830, GSM332698, GSM332848 | |
| | GSM332372, GSM332296, GSM331823, GSM332498, GSM332740, GSM332400, GSM332126, GSM332289, GSM331962, GSM332285, GSM332544, GSM332272, GSM332700, GSM332310, GSM331825 | |
| | GSM332187, GSM332397, GSM331954, GSM332358, GSM332849, GSM332389, GSM332627, GSM332609 | |
| | GSM332476, GSM332125, GSM332836, GSM332292, GSM332602 | |
| | GSM332378, GSM331861, GSM332615, GSM332532, GSM331979, GSM331841, GSM332111, GSM332164 | |
| | GSM332115, GSM332611, GSM332396, GSM332278, GSM332418 | |

Table 84: Modules and submodules of cell samples of Microarray Innovations in Leukemia identified by $\mathcal{E}^3$-1

| First level | Second level | Cell type |
|---|---|---|
| 3 | GSM331735, GSM332459, GSM332072, GSM332041, GSM332073, GSM332763, GSM332821, GSM332467, GSM332406, GSM331939, GSM332335, GSM332190, GSM332340, GSM332219, GSM331899 | |
| | GSM331736, GSM331956, GSM332454, GSM332222, GSM331960, GSM332543, GSM332720, GSM332113, GSM332575, GSM332760, GSM332722, GSM332734, GSM332084, GSM332255, GSM332583, GSM332561, GSM332112, GSM332175, GSM332712, GSM332730, GSM331907, GSM332307, GSM331849, GSM331771, GSM332146, GSM331788, GSM332332, GSM332373, GSM331980, GSM332343, GSM332706, GSM332702 | |
| | GSM331763, GSM331828, GSM332185, GSM332237, GSM332464, GSM332039, GSM332116, GSM332710, GSM332468, GSM332100, GSM331800, GSM332369, GSM332263, GSM332471, GSM332326, GSM332433, GSM332757, GSM332855, GSM331898 | |
| | GSM332804, GSM332850, GSM332436, GSM332683, GSM332239, GSM332858, GSM3 31792, GSM332513, GSM331914, GSM332540, GSM332204, GSM332088, GSM332502, GSM332215, GSM331844, GSM332557, GSM332610, GSM332045, GSM332444 | |
| | GSM331872, GSM332796, GSM331920, GSM332269, GSM332230, GSM332297, GSM332847, GSM332057, GSM332154, GSM332724, GSM332748, GSM332005, GSM332334, GSM332525 | |
| | GSM332827, GSM332800, GSM331777, GSM331900, GSM332837, GSM332523, GSM332012, GSM332553, GSM332653, GSM332500, GSM332545, GSM332687, GSM331916, GSM332357, GSM332031, GSM332376, GSM332799, GSM332231, GSM331842, GSM332173, GSM332573, GSM332604, GSM332195, GSM332846, GSM332681, GSM332592, GSM331822, GSM332079, GSM332667, GSM332236, GSM332353, GSM331784, GSM332177, GSM332555, GSM332678, GSM332499, GSM331847, GSM331760, GSM332252, GSM331942, GSM332790 | |
| | GSM332153, GSM332694 | |
| | GSM331798, GSM332368 | |

Table 85: Modules and submodules of cell samples of Microarray Innovations in Leukemia identified by $\mathcal{E}^3$-2

| First level | Second level | Cell type |
|---|---|---|
| 4 | GSM331737, GSM332274, GSM332137, GSM332628, GSM332318, GSM331850, GSM332119, GSM332549, GSM332761, GSM332870, GSM332162, GSM332762, GSM332806, GSM332641, GSM331908, GSM332416, GSM332333, GSM331905, GSM332474, GSM331886 | |
| | GSM331746, GSM332670, GSM332167, GSM332156, GSM332819, GSM332104, GSM332108, GSM331802, GSM332465, GSM332781, GSM332232, GSM332480, GSM332342, GSM332783, GSM332003, GSM332390, GSM332741, GSM331858, GSM332478, GSM332060, GSM332854 | |
| | GSM331819, GSM331864, GSM332415, GSM332878, GSM331910, GSM332570, GSM331887, GSM332338, GSM332386, GSM332472, GSM331863, GSM331871, GSM331882, GSM332618, GSM332364, GSM332744, GSM331885, GSM332794, GSM332058, GSM332635, GSM332398 | |
| | GSM331820, GSM332427, GSM331904, GSM332356, GSM332200, GSM331985, GSM332626, GSM332613, GSM332494, GSM332214, GSM332590, GSM332051, GSM332267, GSM332017 | |
| 5 | GSM331738, GSM331949, GSM332509, GSM331919, GSM332209, GSM332481, GSM331813, GSM332876, GSM332631, GSM332417, GSM331923, GSM332284, GSM332775, GSM332682, GSM332560, GSM332066, GSM332030, GSM332197, GSM331911, GSM332835, GSM332695, GSM332061, GSM332344, GSM332270, GSM332431, GSM332107, GSM332601, GSM331812, GSM332429 | |
| | GSM331748, GSM332034, GSM332374, GSM332614, GSM332407, GSM332736, GSM332753, GSM332826, GSM332207, GSM332505, GSM332259, GSM331834, GSM332483, GSM332305, GSM332265 | |
| | GSM331772, GSM332572, GSM331909, GSM332044, GSM332536, GSM332506, GSM332402, GSM331940, GSM332271, GSM332330, GSM332511, GSM332227, GSM332813, GSM332225, GSM332742, GSM332283, GSM332693, GSM332063, GSM332496, GSM331857, GSM332211, GSM332445, GSM332448, GSM332080, GSM332529, GSM332832, GSM331953, GSM332009, GSM332519, GSM332349, GSM332788, GSM331801, GSM332861, GSM332619, GSM332410, GSM331769 | |

Table 86: Modules and submodules of cell samples of Microarray Innovations in Leukemia identified by $\mathcal{E}^3$-3

| First level | Second level | Cell type |
|---|---|---|
| 5 | GSM331790, GSM331836, GSM331869, GSM331883, GSM332286, GSM332383, GSM332419, GSM332867, GSM332623, GSM332134, GSM332242, GSM332789, GSM332518, GSM332860, GSM332725, GSM332675, GSM332132, GSM332868, GSM332280, GSM332828, GSM332102, GSM332256, GSM332632, GSM331943, GSM332669, GSM332199, GSM332663, GSM332106, GSM332404, GSM332737, GSM331877, GSM332062, GSM332160, GSM332142, GSM331838, GSM332666, GSM332099, GSM332805, GSM332758, GSM332000, GSM332537 | |
| | GSM331859, GSM332812, GSM331840, GSM332311, GSM332791, GSM332050, GSM332220, GSM332261 | |
| | GSM331986, GSM332355, GSM331852 | |
| | GSM332042, GSM331924, GSM332455, GSM332103, GSM332686, GSM332446, GSM332823, GSM332323, GSM332807, GSM331903, GSM332658, GSM332268, GSM332501, GSM332491, GSM332439 | |
| | GSM332148, GSM332676, GSM332771, GSM331879, GSM332360, GSM331935, GSM331890, GSM331922, GSM332246, GSM332247, GSM331992, GSM332295, GSM331824, GSM332490, GSM331912, GSM332732, GSM332294, GSM332068, GSM332844, GSM332477, GSM331865, GSM332679, GSM331925, GSM332599, GSM331855, GSM332056, GSM332248, GSM331936, GSM332325, GSM331758, GSM332685, GSM332822 | |
| 6 | GSM331739, GSM332168, GSM332365, GSM332493, GSM332029, GSM332449, GSM332834, GSM331996, GSM332640, GSM332151, GSM332447, GSM332801, GSM332388, GSM331741, GSM332299, GSM332839, GSM331997 | |
| | GSM331951, GSM332094, GSM332466, GSM332008, GSM332644, GSM332304, GSM332680, GSM332288, GSM332025, GSM332881, GSM332582, GSM332538, GSM332321, GSM332569, GSM332487, GSM332815, GSM331959, GSM332308, GSM332772, GSM332442, GSM332643 | |
| | GSM332387, GSM332216, GSM332135, GSM332109, GSM332141, GSM332785, GSM332370, GSM332768 | |
| | GSM332059, GSM332528, GSM332745 | |
| | GSM332522, GSM332438, GSM332747, GSM332266, GSM332776, GSM332426, GSM331969, GSM332462, GSM332105, GSM331774, GSM332816, GSM331757, GSM332746, GSM332479, GSM332133, GSM332036, GSM332152, GSM331870, GSM331927, GSM331821, GSM331817 | |

Table 87: Modules and submodules of cell samples of Microarray Innovations in Leukemia identified by $\mathcal{E}^3$-4

| First level | Second level | Cell type |
|---|---|---|
| 7 | GSM331740, GSM332037, GSM332046, GSM332711, GSM332527, GSM332770, GSM332552, GSM332184, GSM332011, GSM332170, GSM332408, GSM332028, GSM332701, GSM332749, GSM331946, GSM332413, GSM332367, GSM332324, GSM332375, GSM331827, GSM332735, GSM332322, GSM332122, GSM331950, GSM332206, GSM332581, GSM332040, GSM332551, GSM332183, GSM332497, GSM332578, GSM331752, GSM331893, GSM332432, GSM332007, GSM331860, GSM331994, GSM332795, GSM332315, GSM331867, GSM332217 | |
| | GSM331762, GSM332169, GSM332114, GSM332434, GSM331778, GSM331868, GSM332430, GSM332233, GSM331874, GSM332600, GSM332078, GSM332584, GSM332524, GSM332547, GSM332022, GSM332671, GSM331816, GSM332020, GSM332117, GSM332689, GSM332121, GSM332607, GSM332161, GSM332186, GSM331944, GSM331963, GSM332729, GSM332642, GSM332451, GSM332533, GSM332692, GSM331934, GSM332090 | |
| | GSM331767, GSM331843, GSM331957, GSM332567, GSM332226, GSM332043, GSM332661, GSM332512, GSM332657, GSM332287, GSM331977, GSM332300, GSM332405, GSM331947, GSM332082, GSM332336, GSM332470, GSM332208, GSM332721, GSM331913, GSM332352, GSM331768, GSM332194, GSM332391, GSM332869, GSM332810, GSM332093, GSM332718, GSM332514, GSM331929, GSM332648, GSM332159, GSM332290, GSM332779, GSM332021, GSM331873, GSM332048, GSM332221, GSM332864 | |
| | GSM332659, GSM332049, GSM331973, GSM332331, GSM332708, GSM332845, GSM332178, GSM331976, GSM332503, GSM332541, GSM332625, GSM332594, GSM331978, GSM331805, GSM332825, GSM331783, GSM332309, GSM332069, GSM331808, GSM331970, GSM332172, GSM332727, GSM331930, GSM331987, GSM332840, GSM331917, GSM332564, GSM331806, GSM332092, GSM332636, GSM332064, GSM332769, GSM332127 | |
| | GSM332070, GSM332556, GSM332520, GSM332589, GSM332562, GSM332201, GSM332002, GSM332189, GSM331811, GSM332510, GSM332765, GSM332752, GSM332526, GSM332303, GSM332181 | |
| | GSM332879, GSM332558, GSM331809 | |

Table 88: Modules and submodules of cell samples of Microarray Innovations in Leukemia identified by $\mathcal{E}^3$-5

| First level | Second level | Cell type |
|---|---|---|
| 8 | GSM331743, GSM331773, GSM332158, GSM332421, GSM332091, GSM332475, GSM332875, GSM331854, GSM332597, GSM332612, GSM332622, GSM332420, GSM332014, GSM331759, GSM332865, GSM332764, GSM332341 | |
| | GSM332453, GSM332539, GSM332273, GSM331814, GSM332234, GSM332081, GSM332165, GSM332077, GSM332191, GSM332586, GSM332171, GSM332166, GSM332606, GSM332856, GSM332473, GSM332820, GSM331906, GSM331876, GSM331972 | |
| | GSM332276, GSM332223, GSM332250, GSM332655, GSM332756, GSM331761, GSM332327, GSM332563, GSM332257, GSM331889, GSM332595, GSM332414, GSM332253, GSM332076, GSM332766, GSM332381 | |
| | GSM332016, GSM332026, GSM332531, GSM332023, GSM332354, GSM332857, GSM331915, GSM332507, GSM332674, GSM332883, GSM332559, GSM332596, GSM332074, GSM332673, GSM332425, GSM331937, GSM331902, GSM332784, GSM332320, GSM332793, GSM332176 | |
| 9 | GSM331744, GSM331749, GSM332866, GSM332423, GSM331982, GSM332574, GSM331833, GSM332508, GSM332019, GSM331984, GSM332452, GSM332279 | |
| | GSM331938, GSM332203, GSM332392, GSM332580, GSM331755, GSM332096, GSM332637, GSM332660, GSM332350, GSM332829 | |
| | GSM332301, GSM332489, GSM332649 | |
| | GSM332399, GSM331753, GSM332384, GSM332316, GSM331810, GSM331945, GSM332361, GSM331848, GSM332603, GSM331764 | |

Table 89: Modules and submodules of cell samples of Microarray Innovations in Leukemia identified by $\mathcal{E}^3$-6

| First level | Second level | Cell type |
|---|---|---|
| 10 | GSM331745, GSM332120, GSM332873, GSM331839, GSM332401, GSM332880, GSM332047, GSM332275, GSM332484, GSM332774, GSM331832, GSM332650, GSM332703, GSM332688 | |
| | GSM331770, GSM332393, GSM332780, GSM332620, GSM332767, GSM332241, GSM332792, GSM332852, GSM332461, GSM331791, GSM332098, GSM332149, GSM332351, GSM332244, GSM332668, GSM331931, GSM331981, GSM332150, GSM332777, GSM331989, GSM332382, GSM332138, GSM332546, GSM332530, GSM332647 | |
| | GSM331803, GSM332131, GSM332565, GSM332174, GSM332346, GSM332630, GSM332089, GSM332086, GSM332831, GSM332083, GSM332665, GSM332677, GSM332238, GSM332709 | |
| | GSM331853, GSM332010, GSM332317, GSM331941, GSM331785, GSM332254, GSM331837 | |
| | GSM331895, GSM331932, GSM332147, GSM332751, GSM332018, GSM332065, GSM332495, GSM332588, GSM332517, GSM332488, GSM332412, GSM332015, GSM332006, GSM332450, GSM332814, GSM332843, GSM332075, GSM332707, GSM332803, GSM332482, GSM332144, GSM332435, GSM331958, GSM332328, GSM332228, GSM332691, GSM331971, GSM331933, GSM332838, GSM332101 | |
| | GSM332851, GSM332348, GSM332394, GSM332690, GSM332179 | |
| | GSM332128, GSM332440, GSM332124, GSM332871 | |
| | GSM332672, GSM332862, GSM332362, GSM332260, GSM332576, GSM332213, GSM331804, GSM331998, GSM332155, GSM332515, GSM332363, GSM332130 | |
| | GSM332802, GSM332704, GSM332188, GSM332863, GSM331787, GSM332726, GSM332598, GSM332071, GSM332110, GSM331961, GSM331880, GSM332651, GSM332616, GSM332696, GSM331897, GSM332409, GSM331797, GSM332385, GSM332163, GSM331955, GSM331875, GSM332492, GSM332379, GSM332782, GSM332833, GSM332443, GSM332377, GSM331851 | |
| | GSM331881, GSM332395, GSM332202, GSM331765, GSM331948, GSM331988, GSM331795, GSM332743, GSM332437, GSM332212, GSM331775, GSM332038, GSM332684, GSM332535, GSM332366, GSM332345, GSM332277, GSM331921, GSM332314, GSM332424 | |

Table 90: Modules and submodules of cell samples of Microarray Innovations in Leukemia identified by $\mathcal{E}^3$-7

| First level | Second level | Cell type |
|---|---|---|
| 11 | GSM331751, GSM332662, GSM332097 | |
| | GSM332542, GSM332302, GSM332754, GSM332380, GSM332243 | |
| | GSM332587, GSM332298, GSM332571, GSM332140, GSM332027, GSM332874, GSM331995 | |
| 12 | GSM331776, GSM331793, GSM331928, GSM332196 | |
| | GSM332095, GSM332656, GSM332621 | |
| 13 | GSM331779, GSM331983 | |
| | GSM332731, GSM332624, GSM332485 | |
| 14 | GSM331782, GSM332463, GSM332534, GSM332035, GSM332129, GSM332521, GSM331926, GSM332258 | |
| | GSM332251, GSM332882, GSM331952, GSM332705, GSM332824, GSM332809, GSM331807, GSM331975, GSM332664 | |
| | GSM332808, GSM331789, GSM332585, GSM332456 | |
| 15 | GSM331786, GSM331918, GSM332013, GSM332639, GSM331993, GSM332554, GSM332811, GSM332738, GSM332136, GSM331831, GSM332205 | |
| | GSM331964, GSM331968, GSM332192, GSM332842, GSM332723, GSM332577, GSM332759, GSM331894 | |
| | GSM332786, GSM332291, GSM332052, GSM332293, GSM332240 | |
| 16 | GSM331794, GSM332229, GSM332306, GSM331829, GSM331888, GSM332853 | |
| | GSM331815, GSM332087, GSM332329 | |
| | GSM332859, GSM332347, GSM331856, GSM332884 | |
| | GSM332728, GSM332319, GSM332605 | |
| | GSM332024, GSM332566 | |
| 17 | GSM331818, GSM332634, GSM332617, GSM332548 | |
| | GSM331846, GSM332638, GSM332877, GSM332313, GSM332652, GSM332067, GSM332457 | |
| | GSM331878, GSM332699, GSM332629, GSM332645, GSM332123 | |
| 18 | GSM331830, GSM332282, GSM332053, GSM332264, GSM332371, GSM332055 | |
| | GSM331866, GSM331974, GSM332032, GSM332516, GSM332422, GSM332460, GSM332182 | |
| | GSM332139, GSM332249, GSM332441 | |

Table 91: Modules and submodules of cell samples of Microarray Innovations in Leukemia identified by $\mathcal{E}^3$-8

# References


[1] Golub, T. R. et al. Molecular classification of cancer: Class discovery and class prediction by gene expression. *Science*, **286**(5439), 531-537 (1999).

[2] Alizadeh, A. et al. Distinct types of diffuse large b-cell lymphoma identified by gene expres sion profiling. *Nature*, **403**, 503-511, (2000).

[3] Ramaswamy, S. et al. Multi-class cancer diagnosis using tumor gene expression signatures. *Proc. Nat. Acad. Sci.*, **98**(26), 15149 (2001).

[4] Yeoh, E.-J. et al. Classification, subtype discovery, and prediction of outcome in pediatric acute lymphoblastic leukemia by gene expression profiling. *Cancer Cell*, **1**(2), (2002).

[5] Bhattacharjee, A. et al. Classification of human lung carcinomas by mRNA expression profiling reveals distinct adenocarcinomas sub-classes. *Proc. Nat. Acad. Sci.*, **98**(24), 13790-13795 (2001).

[6] Su, A. I. et al. Large-scale analysis of the human and mouse transcriptomes. *Proc. Nat. Acad. Sci.*, **99**(7), 4465 (2002).

[7] Pomeroy, S. L. et al. prediction of central nervous system embryonal tumour outcome based on gene expression. *Nature*, **415**, 436 - 442, (2002).

[8] Haferlach, T. et al. Clinical utility of microarry-based gene expression profiling in the diagnosis and subclassification of leukemia: Report from the international microarry innovations in leukemia study group. *J. of Clin. Oncology*, **28** (15), 2529 - 2537 (2010).

[9] Monti, S., Tamayo, P., Mesirov, J. & Golub, T. Consensus Clustering: A Resampling-Based Method for Class Discovery and Visualization of Gene Expression Microarray Data. *Machine Learning* , **52**(1-2), 91-118 (2003).
